# Circulation of low pathogenic avian influenza (LPAI) viruses in wild birds and poultry in the Netherlands, 2006–2016

**DOI:** 10.1038/s41598-019-50170-8

**Published:** 2019-09-23

**Authors:** Saskia A. Bergervoet, Sylvia B. E. Pritz-Verschuren, Jose L. Gonzales, Alex Bossers, Marjolein J. Poen, Jayeeta Dutta, Zenab Khan, Divya Kriti, Harm van Bakel, Ruth Bouwstra, Ron A. M. Fouchier, Nancy Beerens

**Affiliations:** 1 Department of Virology, Wageningen Bioveterinary Research, Lelystad, The Netherlands; 2 Department of Viroscience, Erasmus MC, Rotterdam, The Netherlands; 3 Department of Epidemiology, Wageningen Bioveterinary Research, Lelystad, The Netherlands; 4 Department of Infection Biology, Wageningen Bioveterinary Research, Lelystad, The Netherlands; 5 Department of Genetics and Genomic Sciences, Icahn School of Medicine at Mount Sinai, New York, USA; 6 Icahn Institute for Genomics and Multiscale Biology, Icahn School of Medicine at Mount Sinai, New York, USA; 7 GD Animal Health Service, Deventer, The Netherlands

**Keywords:** Viral genetics, Next-generation sequencing, Pathogens, Influenza virus

## Abstract

In this study, we explore the circulation of low pathogenic avian influenza (LPAI) viruses in wild birds and poultry in the Netherlands. Surveillance data collected between 2006 and 2016 was used to evaluate subtype diversity, spatiotemporal distribution and genetic relationships between wild bird and poultry viruses. We observed close species-dependent associations among hemagglutinin and neuraminidase subtypes. Not all subtypes detected in wild birds were found in poultry, suggesting transmission to poultry is selective and likely depends on viral factors that determine host range restriction. Subtypes commonly detected in poultry were in wild birds most frequently detected in mallards and geese. Different temporal patterns in virus prevalence were observed between wild bird species. Virus detections in domestic ducks coincided with the prevalence peak in wild ducks, whereas virus detections in other poultry types were made throughout the year. Genetic analysis of the surface genes demonstrated that most poultry viruses were related to locally circulating wild bird viruses, but no direct spatiotemporal link was observed. Results indicate prolonged undetected virus circulation and frequent reassortment events with local and newly introduced viruses within the wild bird population. Increased knowledge on LPAI virus circulation can be used to improve surveillance strategies.

## Introduction

Avian influenza (AI) is an infectious disease of birds caused by influenza A viruses. Wild aquatic birds of the orders *Anseriformes* (ducks, geese and swans) and *Charadriiformes* (gulls and waders) are the natural reservoirs of AI viruses^1^. The prevalence of AI viruses in wild birds varies by species, age, season and geographical location^1^. During wild bird migration, AI viruses can be carried over large geographical distances, enabling virus transmission to susceptible host populations across the globe^2^. AI viruses can be transmitted from wild birds to poultry when breeding, stopover and wintering regions overlap with areas of commercial poultry production.

AI viruses are classified into subtypes based on the antigenic structures present on the surface of the virus^3^. Currently, 16 hemagglutinin (HA) and 9 neuraminidase (NA) antigenic subtypes have been identified in birds, which can be found in numerous combinations^2,4^. Most AI viruses are low pathogenic avian influenza (LPAI) viruses that remain subclinical or cause mild infection of the intestinal or respiratory tract^5^. LPAI viruses of subtypes H5 and H7 can evolve into highly pathogenic avian influenza (HPAI) virus variants that are associated with multi-organ systemic infection, which can cause severe disease and high mortality in birds^5^.

Outbreaks of AI virus infections can have serious consequences for animal health and may result in major economic losses for the poultry industry. In addition, human cases of AI virus infections have been reported upon direct or indirect exposure to infected poultry^6^. The rapid and unpredictable evolution of AI viruses leads to the emergence of new influenza virus strains and subtype combinations^7–9^. Alterations in the genetic material of a virus can lead to changes in the virus characteristics, such as increased virulence or expanded host range, and may give rise to virus variants that are more prone to infect poultry. The recurrence of AI outbreaks in poultry highlights the importance of global surveillance efforts for early detection and rapid response.

In the Netherlands, the circulation of AI viruses in wild birds and poultry has been monitored for more than a decade^10,11^. The collection of wild bird swab specimens enables virological detection of AI viruses within the wild bird population. AI virus detection and monitoring in commercial poultry includes both active and passive surveillance methods. Active surveillance is performed by serological screening for AI viruses. The sampling frequency depends on poultry type, housing system and estimated risk for virus introduction^11,12^. Farms holding indoor layer chickens, broiler chickens or ducks are tested once a year for the presence of influenza virus-specific antibodies, while outdoor layer chicken and turkey farms are tested four times a year and each production cycle, respectively. Passive surveillance consists of virological testing of poultry upon notification of AI suspicions based on clinical signs or to confirm positive serology. AI virus surveillance in poultry focuses mainly on the early detection of viruses of subtypes H5 and H7, because of their potential to become highly pathogenic. However, samples collected in these programs are also used to monitor introductions of LPAI viruses of other subtypes.

Although a close relationship between AI viruses originating from wild birds and poultry has been described^13–16^, wild bird species that act as sources of infection for poultry and the actual virus transmission route has not yet been identified. In this study, surveillance data collected in the Netherlands between 2006 and 2016 was analysed to obtain more insight in the circulation of LPAI viruses in wild birds and poultry. We analysed the subtype diversity among LPAI viruses from wild birds and poultry to identify potential hosts for viruses that infect poultry. In addition, spatiotemporal patterns of LPAI virus detections in wild birds and poultry were inferred to identify potential geographical locations or periods in a calendar year associated with infection of poultry. Finally, the genetic relationship between LPAI viruses isolated from wild birds and poultry was determined by phylogenetic analysis of the HA and NA sequences. Expanded knowledge on the circulation of LPAI viruses in wild birds and poultry can be used to improve surveillance strategies and control virus spread in the Netherlands.

## Methods

### Ethical statement

The capture of live wild birds was approved by the Dutch Ministry of Economic Affairs (Flora and Fauna permit FF/75A/2009/067). Wild bird handling and sampling methods were approved by the Animal Experiment Committee of the Erasmus MC (permit numbers 122-07-09, 122-08-12, 122-09-20, 122-10-20 and 122-11-31). Sampling of poultry was carried out in accordance with the European Union Council Directive 2005/94/EC^17^.

### Collection of wild bird and poultry samples

Active virological surveillance of AI virus infections in live wild birds was conducted by Erasmus MC. Individual faecal, cloacal, oropharyngeal or tracheal swabs from wild birds were collected, transported and stored as described previously^18^. Samples collected from wild birds found dead were not included in this study. Serological monitoring of AI virus infections in commercial poultry was conducted by the Dutch Animal Health Service (GD). Blood samples were collected from all poultry farms one or more times a year, depending on the type of farm. Seropositive samples were forwarded to the national reference laboratory Wageningen Bioveterinary Research (WBVR) for confirmatory testing and stored at −20 °C. Virological surveillance of AI virus infections in commercial poultry was conducted when clinical signs were notified or antibodies against virus subtypes H5 or H7 were detected. Individual cloacal, oropharyngeal or tracheal swabs from poultry were collected by a specialist team of the Netherlands Food and Consumer Product Safety Authority (NVWA). Swabs were tested for the presence of influenza virus at WBVR and stored at −80 °C. Information on species, location and date was provided for all samples collected.

### Antibody detection

Antibody detection in poultry serum samples was performed using the FlockChek AI MultiS-Screen Ab Test Kit (IDEXX) according to the manufacturer’s protocol. Serum samples identified as influenza virus-positive were subsequently tested in a H5 and H7 subtype-specific hemagglutination inhibition (HI) test according to the OIE Manual of Standards for Diagnostic Tests and Vaccines^19^. Further antibody characterization was done using a multiplex serological assay based on HA and NA antigens^20^. The results were confirmed using HI tests, neuraminidase inhibition (NI) tests and NA-specific ELISAs^19^.

### Virus detection and isolation

Wild bird virus detection and isolation were performed as described previously^18^. To detect poultry viruses, RNA was extracted from swab specimens or allantoic fluids using the MagNA Pure 96 instrument (Roche) with the MagNA Pure 96 DNA and Viral NA Small Volume Kit (Roche). Influenza virus was detected by the real-time reverse transcription polymerase chain reaction method targeting the matrix gene (M-PCR)^21^. M-PCR positive poultry samples were subsequently tested for the presence of virus subtypes H5 and H7 by the subtype-specific PCRs as recommended by the European Union reference laboratory^22,23^. The pathogenicity of the virus was determined by amplification of a gene fragment spanning the HA proteolytic cleavage site^24^. Subtyping was done by using universal primer sets for amplification of HA and NA gene fragments of all influenza A viruses, as previously described^24,25^. PCR fragments were sequenced by standard Sanger sequencing and compared to publicly available sequences using the BLAST algorithm for subtype identification. To isolate viruses, M-PCR positive samples were inoculated into the allantoic cavity of specific-pathogen-free (SPF) embryonated chicken eggs (ECEs)^19^. Allantoic fluid was collected and tested for hemagglutination activity by standard procedures^19^. Virus isolates were characterized in a HI test using in-house prepared antisera. A second passage in eggs was performed in case no virus was detected in the first passage.

### Sequencing

The HA and NA sequences of LPAI viruses were generated by next-generation sequencing (NGS). Wild bird viruses were selected for NGS based on surveillance data. We selected 129 wild bird viruses of subtypes that were also detected in poultry and 33 wild bird viruses of subtypes that were not detected in poultry to a maximum of two viruses per subtype, species, year and geographical region. Consensus sequences of wild bird viruses were generated as described previously^26^. For sequencing of poultry viruses, 42 LPAI viruses obtained from 58 virus-positive field samples were included. RNA was isolated from swab specimens or allantoic fluid using the High Pure Viral RNA Kit (Roche). The SuperScript III One-Step RT-PCR System with the Platinum Taq DNA Polymerase kit (Invitrogen) and purified universal primers were used for multi-segment amplification of influenza viruses^27^. The PCR products were visualized on agarose gel and purified using the High Pure PCR Product Purification Kit (Roche). Purified amplicons were prepared for sequencing using the Illumina Nextera DNA Sample Preparation kit. Sequencing was performed with a minimum sequence coverage of 1,000x using the paired-end 200 Illumina MiSeq platform. To determine the consensus sequence for each HA and NA gene segment, reads were mapped using the ViralProfiler-Workflow, an extension of the CLC Genomics Workbench (Qiagen, Germany), as described previously^28^. Consensus sequences were generated by a reference-based method using a set of Eurasian AI virus reference sequences. Sequences of wild bird and poultry viruses generated in this study were submitted to Genbank (https://www.ncbi.nlm.nih.gov) (Supplementary Table S1) and GISAID’s EpiFlu Database^29^ (http://www.gisaid.org) (Supplementary Table S2), respectively.

### Phylogenetic analysis

To construct phylogenetic trees of HA and NA gene segments, cluster representatives for each virus subtype were selected from around 21,000 HA and 17,000 NA sequences of AI viruses available in GISAID’s EpiFlu Database^29^ as of July 2016. Sequences outside the 75-125% range of the cluster median sequence length, containing sequencing errors or gaps were excluded for analysis. Remaining sequences were clustered at 90% sequence identity using CD-HIT version 4.6.6 per gene segment^30^. Each cluster was represented by one sequence, known as the centroid sequence or cluster representative. The BLAST algorithm was used to select the top 50 sequence matches from publicly available HA and NA sequences for each poultry virus. Nucleotide sequences of cluster representatives, poultry viruses and BLAST hits were aligned using CLC Genomics Workbench version 8.5. Alignments were edited manually for frameshifts, sequence duplicates and length. A phylogenetic tree was constructed for each HA and NA gene segment using the Neighbour-Joining method^31^ within the MEGA7 software package^32^ using the Tamura-Nei substitution model with a gamma distribution (shape parameter = 1) for rate variation. Bootstrap support values (1,000 replicates) of more than 70 are shown at the branches.

### Data analysis

Cases were defined as subtyped if the HA or NA subtype of the virus or the subtype-specificity of the influenza virus-specific antibodies was determined. The number of virus detections mentioned in this study may differ from previous studies that have also included non-subtyped M-PCR positive samples^12,16,33^. The association between bird species and virus subtype was assessed performing corresponding analysis where host-virus subtype dependencies where graphically explored in a two dimensional plot. To estimate the temporal prevalence of LPAI viruses circulating in the wild bird population, cases were treated as epidemiological units defined as sampling clusters (groups of birds of same species sampled at one time and one place) where subtyped viruses were detected. Cluster prevalence was quantified at a monthly level for each year of the study for each wild bird species monitored. Data analysis was done using the statistical software package R version 3.4.0^34^. The geographical distribution of LPAI viruses in wild birds and poultry was explored by mapping the sampling efforts (total number of wild birds or poultry farms sampled) and the number of subtyped cases during the study period. Geographical maps were plotted using the QGIS desktop application version 2.18.2.

## Results

### Collection and subtyping of wild bird and poultry samples

**Figure 1:**
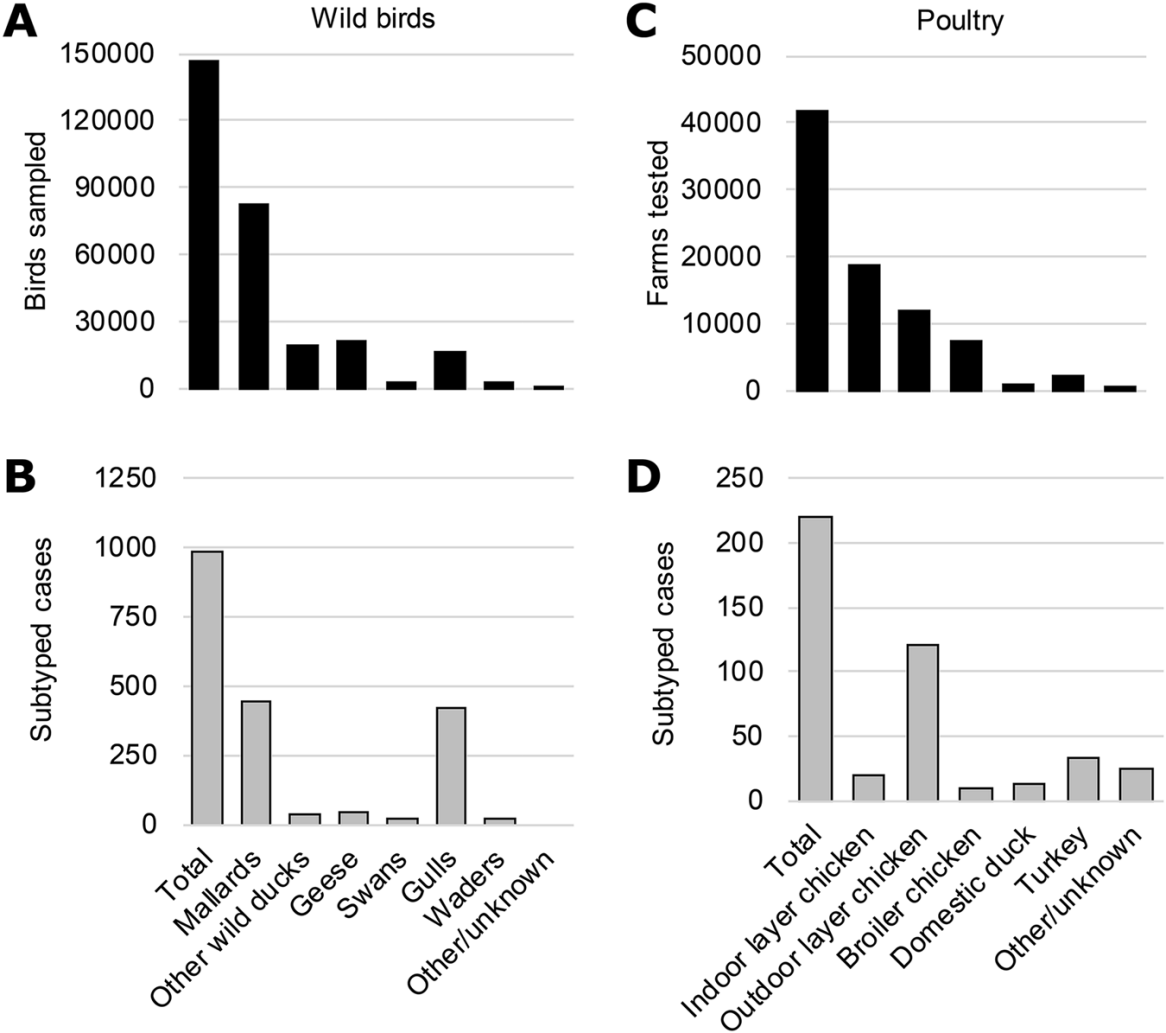
Collection and subtyping of wild bird and poultry samples. (**A**) Number of wild birds sampled and (**B**) number of subtyped cases of low pathogenic avian influenza (LPAI) virus detections in wild birds per wild bird species. (**C**) Number of poultry farms tested and (**D**) number of subtyped cases of LPAI virus detections in poultry farms per poultry type. All samples were collected as part of the national avian influenza (AI) surveillance program in the Netherlands, January 2006-September 2016. A case is considered subtyped if the hemagglutinin (HA) or neuraminidase (NA) subtype of the virus or the subtype-specificity of the influenza virus-specific antibodies is determined.

During the surveillance period, in total 111,114 wild birds (9,281 sampling clusters) belonging to 148 species of 17 orders were sampled for virological testing (Supplementary Table S3). Most birds belonged to species of the order *Anseriformes* (77%), of which the majority were mallards (55%), followed by geese (26%), other wild duck species (16%), and swans (3%) (Fig. 1A). Fewer birds belonged to species of the order *Charadriiformes* (19%), of which 86% were gulls, 12% waders and 2% other *Charadriiformes* species. The HA or NA subtype was characterized for 981 swab samples collected from 21 wild bird species. Most subtyped samples were obtained from mallards (45%) and gulls (43%) (Fig. 1B).

In contrast to the wild bird monitoring program, surveillance in poultry was performed by both serological and virological testing. As part of serological monitoring in poultry, in total 41,769 farms were tested, including farms holding indoor layer chickens (45%), outdoor layer chickens (28%), broiler chickens (17%), turkeys (6%) and ducks (2%) (Fig. 1C; Supplementary Table S4). For virological monitoring in poultry, swab samples from 980 farms were tested to confirm positive serology or suspicions raised by clinical surveillance. The HA or NA subtype was characterized for 220 LPAI virus detections in 152 poultry farms. Subtyped cases were most often detected in chicken farms (76%), in particular layer farms with a free-ranging facility, followed by turkey farms (15%) and duck farms (6%) (Fig. 1D). Most infections in poultry were detected through antibody detection (162 subtyped cases), whereas a quarter of the cases were subtyped based on virology (58 subtyped cases).

### Analysis of LPAI virus subtypes circulating in wild birds and poultry

**Figure 2:**
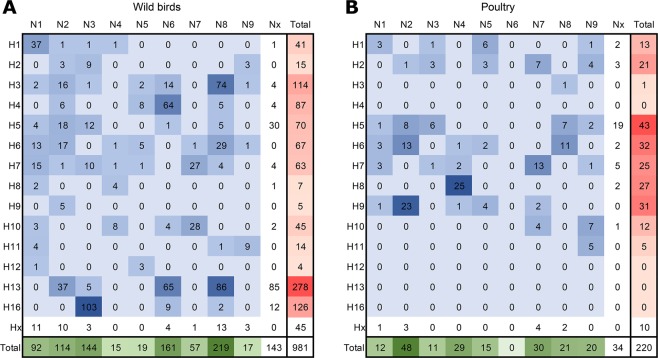
Virus subtypes and subtype combinations detected in wild birds and poultry. Number of hemagglutinin (HA) subtypes, neuraminidase (NA) subtypes, and HA/NA subtype combinations of low pathogenic avian influenza (LPAI) viruses detected in (**A**) wild birds and (**B**) poultry, as part of virological and serological surveillance for avian influenza (AI) virus infections in the Netherlands, January 2006-September 2016. HA subtypes (red), NA subtypes (green), HA/NA subtype combinations (blue) were coloured according to the frequencies of detection.

**Figure 3:**
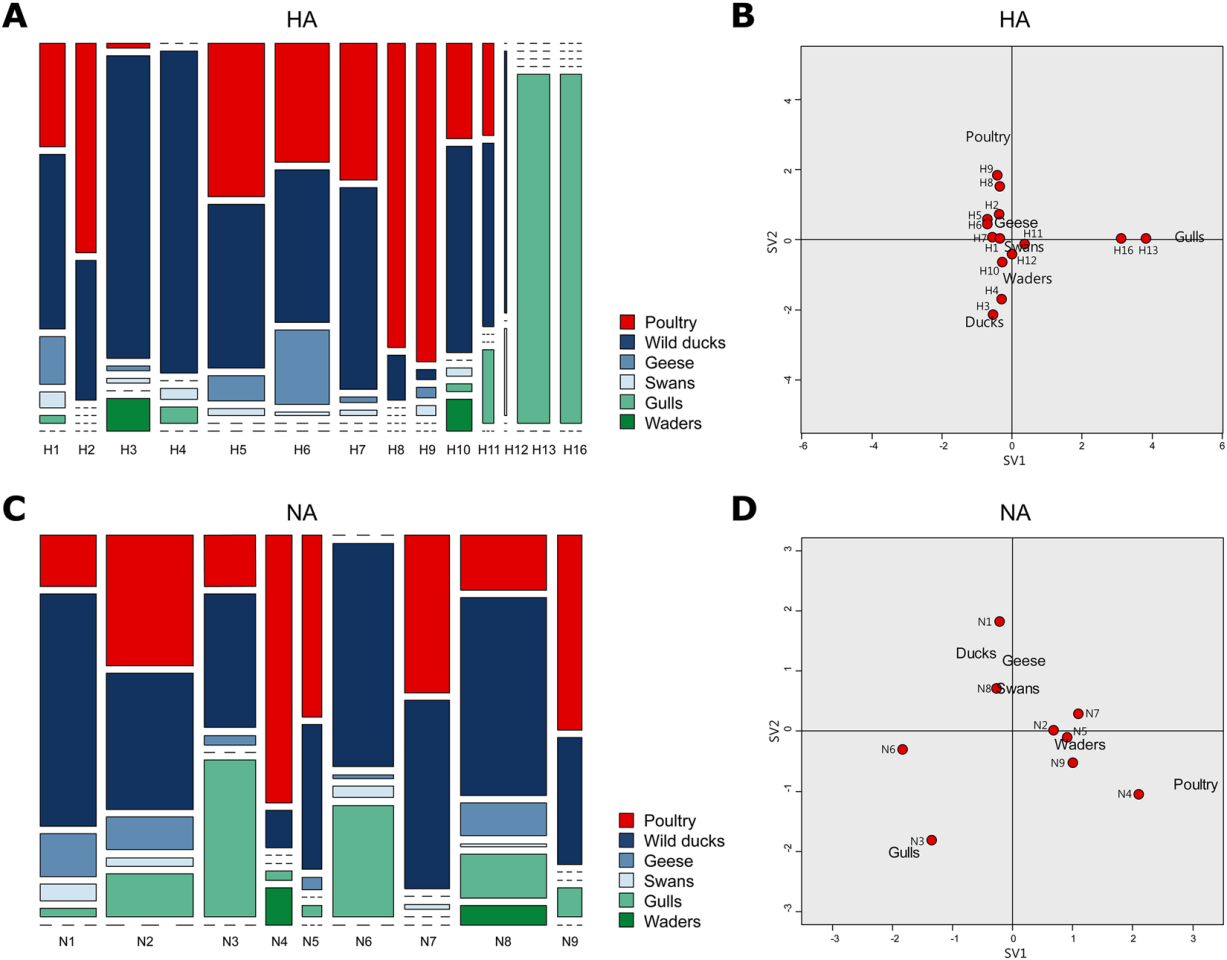
Virus subtype distribution among wild bird species and poultry. (**A**) Relative hemagglutinin (HA) subtype distribution among wild bird species and poultry. The bar width represents the number of cases within each HA subtype. (**B**) Correspondence plot showing the association between bird species and HA subtype in two dimensions (singular value (SV)1 and SV2). (**C**) Relative neuraminidase (NA) subtype distribution among wild bird species and poultry. The bar width represents the number of cases within each NA subtype. (**D**) Correspondence plot showing the association between bird species and NA subtype in two dimensions (SV1 and SV2). All subtyped cases were detected as part of the national avian influenza (AI) surveillance program in the Netherlands, January 2006-September 2016.

To obtain more insight into the circulation of LPAI virus subtypes in the Netherlands, we analysed the HA and NA subtypes and subtype combinations that were detected in wild birds (Fig. 2A) and poultry (Fig. 2B). The HA subtype was identified for 937 wild bird viruses and 211 virus detections in poultry. All 16 HA subtypes except H14 and H15 were detected during surveillance in live wild birds. Of the most frequently identified HA subtypes in wild birds, H13 (30%) and H16 (13%) were exclusively detected in gulls, whereas H3 (12%) and H4 (9%) were primarily detected in wild ducks (Fig. 3A). H8, H9 and H12 were detected in wild birds only sporadically (frequency of <1%). In poultry, the most frequently detected HA subtypes were H5 (20%), H6 (15%), H9 (14%), H8 (12%) and H7 (11%). HA subtypes H4 and H12-H16 were not detected in poultry, and H3 was detected only once in domestic ducks. A two dimensional correspondence analysis plot combining HA subtypes and bird species shows that H13 and H16 viruses are closely associated with gulls, and indicates an association of H3 and H4 viruses with wild ducks, and H8 and H9 viruses with poultry (Fig. 3B). Other HA subtypes fell around the centre of the correspondence plot, indicating their occurrence is host-independent.

The NA subtype was identified for 838 wild bird viruses and 186 virus detections in poultry. The most frequently detected NA subtypes in wild birds were N8 (26%), N6 (19%), and N3 (17%). In poultry, the most frequently identified NA subtypes were N2 (26%), N7 (16%) and N4 (16%). These NA subtypes were found in all wild bird species except waders (Fig. 3C). Correspondence analysis of NA subtypes and bird species combined indicates an association of N3 viruses with gulls and N4 viruses with poultry (Fig. 3D). Due to the absence of detection, N1 was negatively associated with gulls and N6 was negatively associated with poultry. For other NA subtypes, no clear association between bird species and virus subtype was observed.

We identified 55 HA/NA subtype combinations for 796 wild bird viruses and 35 HA/NA subtype combinations for 177 virus detections in poultry. The most frequently detected HA/NA subtype combinations in wild birds were H16N3 (13%), H13N8 (11%), H13N6 (8%) in gulls, and H3N8 (9%) and H4N6 (8%) in other wild bird species. The most frequently detected HA/NA subtype combinations in poultry were H8N4 (14%) and H9N2 (13%), followed by H7N7 (6%), H6N2 (6%) and H6N8 (5%). Of these subtype combinations, H8N4 and H9N2 were rarely detected in wild birds (frequency of <1%). In contrast, H6N2, H6N8 and H7N7 were frequently isolated from wild birds, in particular from mallards and geese.

### Spatiotemporal analysis of LPAI viruses in the Netherlands

**Figure 4:**
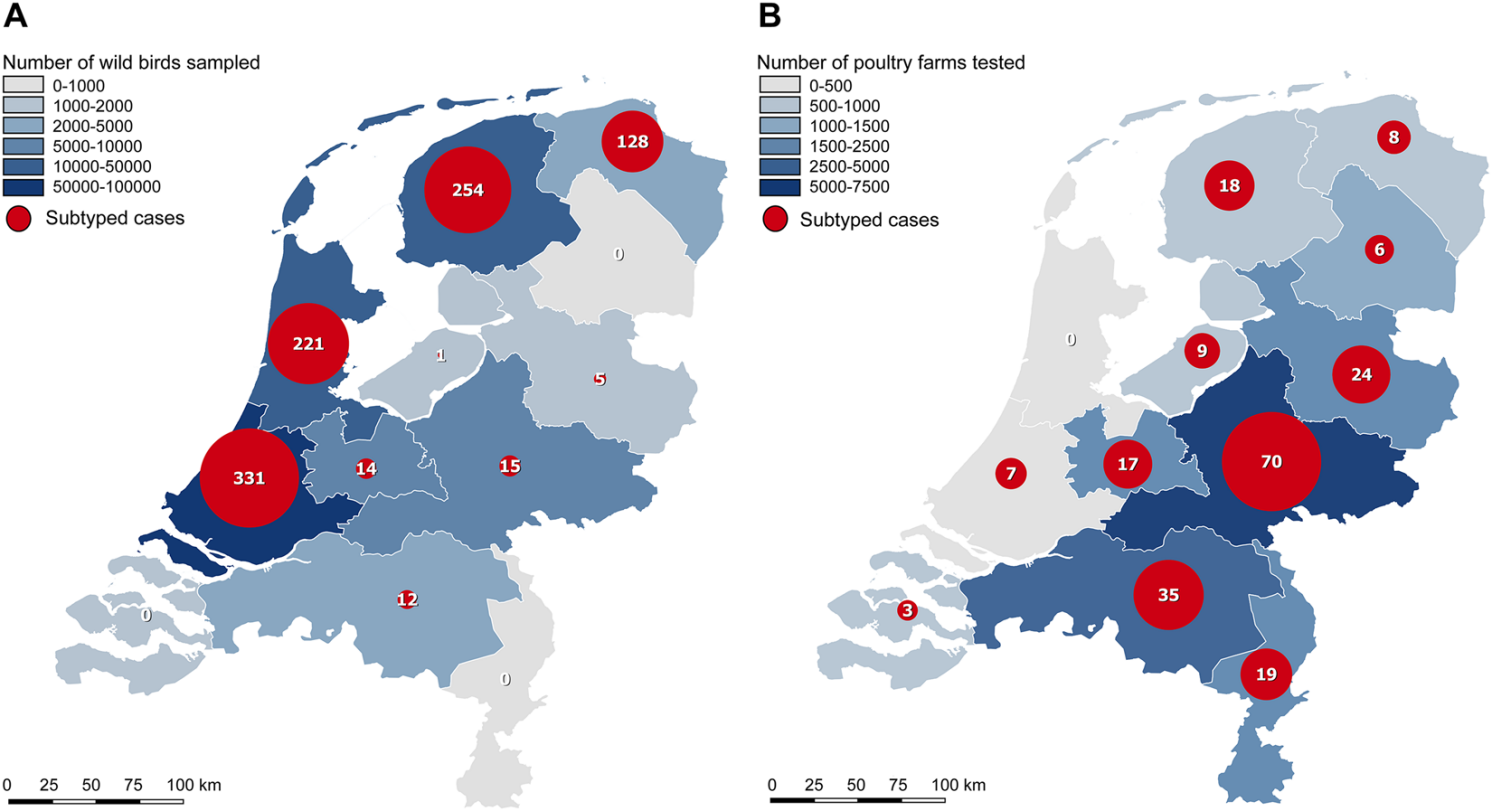
Geographical distribution of sampling efforts and subtyped cases in wild birds and poultry. Geographical distribution of (**A**) wild bird samples collected (blue) and the number of subtyped cases of low pathogenic avian influenza (LPAI) virus detections in wild birds (red), and (**B**) poultry farms tested (blue) and the number of subtyped cases of LPAI virus detections in poultry farms (red), by province, as part of the national avian influenza (AI) surveillance program in the Netherlands, January 2006-September 2016. A case is considered subtyped if the hemagglutinin (HA) or neuraminidase (NA) subtype of the virus or the subtype-specificity of the influenza virus-specific antibodies is determined.

Wild bird samples were mainly collected in water-rich areas along the coastline of the Netherlands, in the provinces Zuid Holland (51%), Noord Holland (15%) and Friesland (9%) (Fig. 4A), while most tested farms were located in poultry dense areas in the central and south-eastern part of the Netherlands, in the provinces Gelderland (24%) and Noord Brabant (22%) (Fig. 4B). Wild bird viruses were relatively more frequently detected in the provinces Groningen and Friesland, whereas the distribution of virus detections in poultry was proportional to the distribution of the farms.

**Figure 5:**
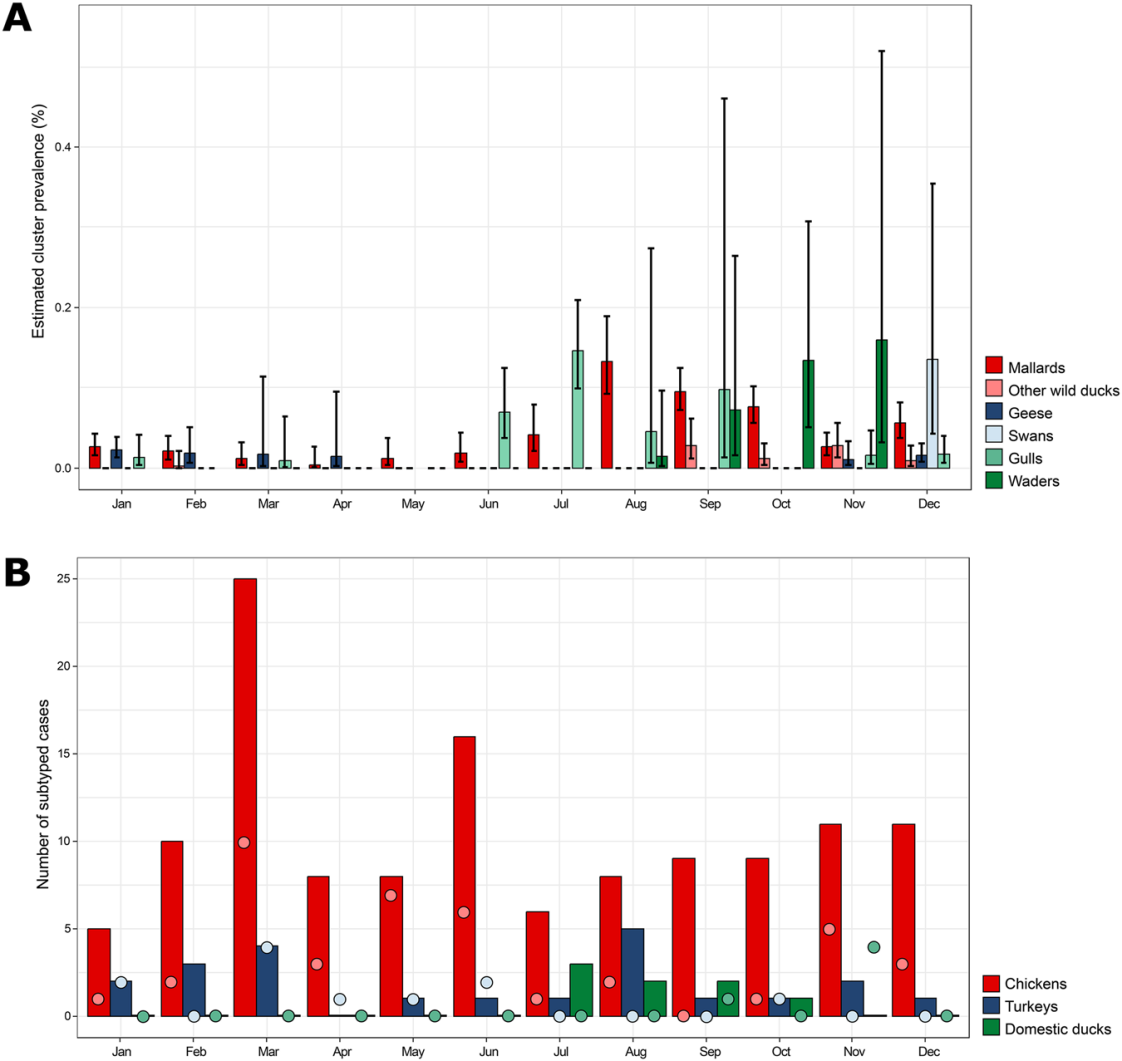
Temporal patterns of virus detections in wild birds and poultry. (**A**) Estimated cluster prevalence of low pathogenic avian influenza (LPAI) viruses in wild birds per month of the year. For this analysis, subtyped cases in wild birds were clustered based on identical host species, subtype combination, sampling location and collection date. The error bars show the standard deviation between different years. (**B**) Number of subtyped cases in poultry per month of the year based on serology (bars) and virology (dots). The black line represents the average number of poultry farms tested per month of the year. The error bars show the standard deviation between different years. Data was collected as part of the national avian influenza (AI) surveillance program in the Netherlands, January 2006-September 2016. A case is considered subtyped if the hemagglutinin (HA) or neuraminidase (NA) subtype of the virus or the subtype-specificity of the influenza virus-specific antibodies is determined.

To analyse temporal patterns in the detection of LPAI viruses, we estimated the monthly cluster prevalence for each wild bird species for each year of the study period. A significant increase in prevalence of virus detections in wild birds was found in August, September, October and December compared to January (p < 0.05). LPAI viruses in gulls were most frequently detected in summer season (June-September) (Fig. 5A). Although belonging to the same species order, LPAI viruses in waders were more often detected during autumn (September-November). LPAI viruses in mallards and other wild ducks were primarily observed between late summer and early winter (August-December). LPAI viruses in geese and swans were most often detected in winter and spring season (November-April). In chickens and turkeys, LPAI virus detections were made throughout the year, with an increase in incidence in chickens in March based on both serological and virological surveillance data (Fig. 5B). LPAI virus detections in domestic ducks were solely observed during summer and autumn (July-November).

### Genetic analysis of HA and NA gene segments

To investigate the genetic relationship between LPAI viruses from wild birds and poultry, the sequences of the HA and NA gene segments were determined for all 42 poultry viruses and a selection of 162 wild bird viruses. The sequences of the poultry viruses were subsequently compared to the sequences of wild bird viruses determined in this study, and publicly available sequences. Phylogenetic analysis was performed for all HA subtypes (H1-H3 and H5-H10) (Supplementary Fig. S1) and NA subtypes (N1-N5 and N7-N9) (Supplementary Fig. S2) detected in poultry. Most LPAI viruses isolated from poultry clustered phylogenetically with European virus strains. The HA gene of 21 poultry viruses and the NA gene of 25 poultry viruses clustered phylogenetically with viruses collected in the Netherlands. One poultry virus was genetically most closely related to viruses outside Europe: the HA and NA gene of A/Chicken/Netherlands/14003323/2014 (H5N2) clustered phylogenetically with Asian strains.

To detect potential precursor viruses in wild birds, we determined the most identical wild bird viruses for each poultry virus HA and NA gene by BLAST (Supplementary Table S5). Related wild bird viruses were of different HA/NA subtype combination in approximately half of the cases. In 33 cases, the poultry virus shared the highest sequence identity with the HA and NA gene of two different wild bird viruses. For nine poultry viruses, a single wild bird virus was identified as most identical for both gene segments (Table 1). These poultry viruses showed nucleotide sequence identities of 97.8–99.8% (HA) and 98.2–99.9% (NA) to the most identical wild bird virus. The distance between the sampling sites of these poultry and wild bird viruses varied from 27 to 216 km. Two of the wild bird viruses were collected within a three-months period prior to detection in poultry.

**Table 1:** Poultry viruses and their most identical wild bird virus.

Poultry virus	Collection date poultry virus	Collection location poultry virus (country – province)	Most identical wild bird virus	Collection date wild bird virus	Collection location wild bird virus (country – province)	Time interval (days)	Distance (km)	Identity HA (%)	Identity NA (%)
A/Duck/Netherlands/06027358/2006 (H3N8)	2006-09-27	NL - Gelderland	A/Mallard Duck/Netherlands/60/2006 (H3N8)	2006-09-18	NL - Noord Holland	9	135	99.80	99.80
A/Chicken/Netherlands/10008427/2010 (H10N7)	2010-05-20	NL - Friesland	A/Mallard/Netherlands/67/2008 (H10N7)	2008-12-13	NL - Zuid Holland	523	172	99.00	99.00
A/Chicken/Netherlands/11004004/2011 (H8N4)	2011-03-09	NL - Utrecht	A/Common Teal/Netherlands/12002960/2012 (H8N4)	2012-03-02	NL - Noord Holland	−359	27	99.20	99.50
A/Chicken/Netherlands/11008327/2011 (H7N7)	2011-05-12	NL - Gelderland	A/Mallard Duck/Netherlands/1/2011 (H7N7)	2011-02-23	NL - Noord Holland	78	96	99.40	98.80
A/Chicken/Netherlands/11009919/2011 (H1N1)	2011-05-30	NL – Zuid Holland	A/Greater white-fronted goose/Netherlands/4/2011 (H1N1)	2011-01-17	NL - Noord Brabant	133	50	98.80	99.90
A/Chicken/Netherlands/11011392/2011 (H7N7)^a^	2011-06-22	NL - Flevoland	A/Mallard Duck/Netherlands/1/2011 (H7N7)	2011-02-23	NL - Noord Holland	119	51	99.30	98.80
A/Turkey/Netherlands/11011530/2011 (H7N7)	2011-06-25	NL - Flevoland	A/Mallard Duck/Netherlands/1/2011 (H7N7)	2011-02-23	NL - Noord Holland	122	51	99.30	98.70
A/Chicken/Netherlands/13003601/2013 (H7N7)	2013-03-12	NL - Gelderland	A/Anas platyrhynchos/Belgium/23852cls33/2012 (H7N7)	2012-09-12	BE - Namur	181	216	99.20	99.20
A/Duck/Netherlands/14016168/2014 (H6N8)	2014-11-25	NL - Gelderland	A/Mallard Duck/Netherlands/15/2011 (H6N8)	2011-09-14	NL - Noord Holland	1168	84	97.80	98.20

Low pathogenic avian influenza (LPAI) poultry viruses, detected as part of the national avian influenza (AI) surveillance program in the Netherlands, January 2006-September 2016, for which the same most identical wild bird virus was identified for the hemagglutinin (HA) and neuraminidase (NA) gene segment sequences by BLAST. The time interval between collection dates, the distance between the collection locations and nucleotide sequence identities between the HA and NA gene segments of the poultry and wild bird viruses are shown. We gratefully acknowledge the authors, originating and submitting laboratories of the sequences from GISAID’s EpiFlu Database [28] on which this research is based. All submitters of data may be contacted directly via the GISAID website (http://www.gisaid.org).

BE = Belgium, NL = The Netherlands. ^a^A/Chicken/Netherlands/11011392/2011 (H7N7); A/Chicken/Netherlands/11011326/2011 (H7N7).

The most identical wild bird viruses were often isolated from mallards (75%), whereas a smaller subset was isolated from other duck species, swans, geese, and gulls (25%). Genetic analysis also revealed a close relationship between viruses derived from different poultry farms. Poultry viruses that were related based on both the HA and NA gene segment were collected within the same year: H1N5 (2007), H10N7 (2009), H6N1 (2010), H7N7 (2011), H10N9 (2012), H5N3 (2013), and H6N2 (2014). These poultry viruses showed nucleotide sequence identities of 99.5–100.0% (HA) and 99.7–100.0% (NA). In seven cases, poultry viruses clustered together in the phylogenetic tree based on only one of the two gene segments. These poultry viruses were of different HA/NA subtype combination or collected in separate years. An exception is A/Chicken/Netherlands/13003601/2013 (H7N7) that clustered phylogenetically together with A/Chicken/Netherlands/13003983/2013 (H7N7) based on HA, but not NA.

## Discussion

Analysis of surveillance data obtained in the Netherlands between January 2006 and September 2016 demonstrated that wild birds were frequently infected with LPAI viruses and infection of poultry was not uncommon. Most wild bird LPAI viruses were detected in mallards, which were the most sampled species among the waterfowl breeding population in the Netherlands. Mallards belong to the group of dabbling ducks, which are considered the main reservoir hosts of LPAI viruses^35^. As the most abundant dabbling duck species, mallards have been the focus of many influenza monitoring programs^1,2,35^. In poultry, most LPAI viruses were detected in chickens, which represent 98% of the commercial poultry population in the Netherlands^36^. Detections were relatively more frequently made in outdoor layer chickens, domestic ducks and turkeys compared to indoor layers and broiler chickens, as reported previously^12,37,38^. Outdoor-ranged poultry is considered to have an increased risk for AI virus introduction because of its close contact with wild birds^37^. The relatively high rate of introduction in turkey and domestic duck farms is likely due to a higher susceptibility of these poultry species to wild bird LPAI viruses. Experimental studies have demonstrated that turkeys are highly susceptible to influenza viruses of diverse origins^39–41^. Influenza viruses from wild ducks may be more easily transmitted to domestic ducks than other poultry species because of the lack of a species barrier.

During the ten-year surveillance period, a wide range of LPAI virus subtypes was identified. The HA and NA subtypes most frequently found in wild birds and poultry differed, and not all subtypes detected in wild birds were also found in poultry. Differences in the HA and NA subtype distribution between wild birds and poultry suggest that virus transmission is selective, and likely depends on viral factors that determine host range restriction.

Analysis of the HA subtype diversity indicated that H13 and H16 viruses exclusively infect gulls, which is presumably due to a strict host range^4,42,43^. H3 and H4 viruses were primarily isolated from wild ducks and rarely detected in poultry. These observations are consistent with previous surveillance studies conducted in the Netherlands^16^, other European countries^2,35,44–47^ and North America^48^. In contrast, H3 and H4 virus infections have been repeatedly reported in poultry in Asia, mainly affecting domestic ducks^49–55^, and occasionally chickens^49,56–58^. Experimental studies have demonstrated that H3 and H4 viruses are capable of infecting chickens^55,59–61^. However, infection in chickens is often restricted to the upper respiratory tract and replication efficiency differs strongly between virus strains, which may contribute to the observed host bias.

H8 and H9 viruses were frequently detected in poultry, but only sporadically found in wild birds. The low prevalence of H8 and H9 subtypes in wild birds is consistent with previous findings^2,16,35,44,45,62^. Like in previous studies, H8 and H9 subtypes were most commonly found in combination with N4 and N2, respectively^16,63^. H9 viruses have frequently been detected in poultry in Eurasia^64–66^, which may be related to increased monitoring of H9 viruses since certain H9N2 strains have caused clinical disease and significant mortality in poultry^67^. Moreover, transmission of H9N2 viruses from poultry to humans have been reported^68^. According to published sequence data, H8 viruses have rarely been isolated from poultry outside the Netherlands. Like H9 viruses, H8 viruses may also predominantly infect poultry but remain undetected during most monitoring studies because no clinical signs are present. Although high incidence in poultry was observed, H8 and H9 virus infections have also been described in wild birds^69^. Therefore, host range tends to be less stringent for these subtypes. The low number H8 and H9 virus detections in wild birds may represent a limitation of sampling.

Associations between bird species and NA subtypes were often linked to HA subtype, e.g. N3 combined with H16 in gulls (H16N3) and N4 combined with H8 in poultry (H8N4). Additionally, N6 was predominantly found in combination with two HA subtypes that were not detected in poultry, H4 and H13, causing a negative association of N6 with poultry. HA and NA subtypes that were detected in various bird species were located around the centre of the correspondence plot, confirming their species independence. These subtypes tend to have a rather broad host range or may rapidly adapt to a new host.

HA/NA combinations H6N2, H6N8 and H7N7, which were commonly detected in poultry, were in wild birds most frequently detected in mallards and geese. Mallards and geese are recognized reservoirs for influenza viruses^2,3^. Geese mainly feed on pastures and agricultural fields allowing contact with poultry^70^. However, a low prevalence of AI viruses has been reported in goose species^35^, and several studies suggest that the role of geese in virus transmission is limited^71–74^. A large diversity of LPAI virus subtypes was observed in mallards, which are likely exposed to a large variety of influenza viruses during migration. Unlike poultry, mallards preferably reside in water-rich areas and feed in surface water^3,75^. Therefore, geese may act as intermediate hosts that transfer the virus from wild ducks to poultry. Alternatively, geese may be susceptible or exposed to the same viruses as poultry. It should be mentioned that - due to irregular sampling of only a small proportion of the wild bird population and the absence of serological monitoring - the circulation of certain HA and NA subtypes in wild birds may have remain undetected, influencing the corresponding analysis. In addition, the detection of the same subtype in large sampling clusters may also have contributed to a bias in the host-subtype association.

Spatial analysis revealed limited geographical overlap between sites of LPAI virus detections in wild birds and poultry, confirming previous observations^16^. Most wild bird viruses were detected in water-rich areas along the coastline of the Netherlands, containing breeding, stopover and wintering locations of wild birds. Wild bird sampling activities were considerably biased toward these areas because of the abundance of waterfowl and the presence of duck decoys that are used by ornithologists and hunters for wild bird capturing. In addition, the relative high rate of subtyped cases in the provinces Friesland and Groningen could be explained by intensive sampling of gulls in these areas during fledging season. In contrast, LPAI virus introductions in poultry were predominantly detected in the Central and South-Eastern part of the Netherlands, where most poultry farms are located. The differences between the geographical distribution of wild bird and poultry viruses appears to be a result of different sampling strategies.

The analysis of LPAI virus detections over the calendar year revealed discordant temporal patterns between wild bird species and poultry types. LPAI viruses in gulls were most frequently detected in summer, while LPAI viruses in wild ducks were primarily detected between late summer and early winter. These observations are consistent with previous studies^44,46,62,76,77^, and likely related to the fledging period of gull chicks^77^ and the migration period of wild ducks^45^. LPAI viruses in geese and swans were detected in winter and spring season. This period coincides with the period of increased LPAI virus observations in chickens, supporting the hypothesis that geese may have a role in transmission of LPAI viruses to poultry. It should be noted that serological surveillance in poultry can cause late diagnosis of virus infection, because antibodies can often be detected for many weeks or months post infection^78^, when virus has already been cleared. Information on seronegative test results prior to influenza-specific antibody detection may be used to improve estimations of the time of virus introduction, but is limited by the low frequency of sampling (1–4 times a year). Interestingly, LPAI viruses were solely detected in domestic ducks during the seasonal peak of LPAI virus infections in wild ducks. This observation supports the hypothesis that LPAI viruses may be more easily transmitted from wild to domestic ducks.

Genetic analysis of the HA and NA gene segments showed that many LPAI viruses from poultry shared common ancestors with wild bird viruses in the Netherlands. Some poultry viruses were more closely related to wild bird viruses from other countries in Europe and Asia. In these cases, virus circulation has likely been missed during wild bird surveillance in the Netherlands. The HA and NA gene segments of individual poultry viruses were often related to different wild bird viruses, indicating a lack of sequence data on immediate precursor viruses. Most poultry viruses were subtype reassortants compared to their closest related wild bird virus, due to the emergence of novel gene constellations during genetic reassortment^7^. For nine poultry viruses, a single virus was identified as most identical wild bird virus for both gene segments, but no direct spatiotemporal link was observed. These results suggest prolonged undetected virus circulation and frequent reassortment events with local and newly introduced viruses within the wild bird population.

Genetically related wild bird viruses were often isolated from mallards. However, since we did not identify wild bird viruses that were linked both genetically and spatiotemporally, it is not known whether the viruses were introduced into poultry by mallards or via another (intermediate) host. Wild bird sampling activities should be performed year-round and intensified in areas of commercial poultry production with focus on farm grounds with turkeys, ducks and outdoor chickens, to allow the detection of genetically related wild bird viruses that can also be linked spatiotemporally to poultry viruses. In addition, samples from wild bird species other than mallards should be collected to identify potential wild bird species of importance for virus transmission to poultry.

Most poultry farms were likely infected by separate virus introductions from wild birds. However, some poultry viruses were genetically highly related based on the HA and NA gene segments, suggesting they were introduced from the same wild bird source or by between-farm transmission. A previous genetic analysis of the internal gene segments confirmed their close genetic relationship^79^. In addition, combined genetic and epidemiological analysis has provided information on the possible routes of introduction for these viruses. Better understanding of factors associated with virus transmission into poultry is important to control virus spread and improve surveillance strategies in the Netherlands.

## Supplementary information

Supplementary information

## Data Availability

All data generated or analysed during this study are fully available without restriction.
