# Supplementary material for: Circulation of low pathogenic avian influenza (LPAI) viruses in wild birds and poultry in the Netherlands, 2006–2016
Source: Sci Rep. 2019 Sep 23;9:13681. doi: 10.1038/s41598-019-50170-8 (PMC6757041; doi:10.1038/s41598-019-50170-8)
Supplement: Supplementary file 1 — Supplementary information [file 41598_2019_50170_MOESM1_ESM.pdf]

Supplementary information

Circulation of low pathogenic avian influenza (LPAI) viruses in wild birds and poultry in the Netherlands, 2006-2016

Saskia A. Bergervoet[1,2], Sylvia B.E. Pritz-Verschuren[1], Jose L. Gonzales[3], Alex Bossers[4], Marjolein J. Poen[2], Jayeeta Dutta[5], Zenab Khan[5], Divya Kriti[5], Harm van Bakel[5,6], Ruth Bouwstra[7], Ron A.M. Fouchier[2], Nancy Beerens[1*]

[1]Department of Virology, Wageningen Bioveterinary Research, Lelystad, The Netherlands
[2]Department of Viroscience, Erasmus MC, Rotterdam, The Netherlands
[3]Department of Epidemiology, Wageningen Bioveterinary Research, Lelystad, The Netherlands
[4]Department of Infection Biology, Wageningen Bioveterinary Research, Lelystad, The Netherlands
[5]Department of Genetics and Genomic Sciences, Icahn School of Medicine at Mount Sinai, New York, USA
[6]Icahn Institute for Genomics and Multiscale Biology, Icahn School of Medicine at Mount Sinai, New York, USA
[7]GD Animal Health Service, Deventer, The Netherlands

*Corresponding author:
Email: nancy.beerens@wur.nl

Contents

Supplementary Table S1. Genbank accession numbers of wild bird virus sequences.
Supplementary Table S2. GISAID accession numbers of poultry virus sequences.
Supplementary Table S3. Collection and subtyping of wild bird samples.
Supplementary Table S4. Collection and subtyping of poultry samples.
Supplementary Table S5. Poultry viruses and their most identical wild bird virus.
Supplementary Figure S1. Phylogenetic trees of HA genes.
Supplementary Figure S2. Phylogenetic trees of NA genes.

Supplementary Table S1. Genbank accession numbers of wild bird virus sequences.
Genbank accession numbers of hemagglutinin (HA) and neuraminidase (NA) gene segment sequences of low pathogenic avian influenza (LPAI) viruses from wild birds detected as part of the national avian influenza (AI) surveillance program in the Netherlands, January 2006-September 2016.

Isolate name	Collection date	Genbank HA segment ID	Genbank NA segment ID
A/Barnacle goose/Netherlands/1/2006 (H1N1)	2006-01-09	KX979589	KX977747
A/Barnacle goose/Netherlands/1/2010 (H6N8)	2010-01-08	KX977787	KX979558
A/Barnacle goose/Netherlands/1/2011 (H6N8)	2011-01-06	KX979107	KX979461
A/Barnacle goose/Netherlands/1/2014 (H6N8)	2014-12-15	KX978284	KX978681
A/Bean goose/Netherlands/1/2007 (H1N1)	2007-01-15	KX978559	KX978842
A/Bean goose/Netherlands/1/2008 (H6N8)	2008-01-22	KX977816	KX978984
A/Bean goose/Netherlands/1/2009 (H7N1)	2009-01-21	KX979368	KX979548
A/Bean goose/Netherlands/2/2009 (H7N1)	2009-01-21	KX978247	KX978908
A/Bewicks swan/Netherlands/2/2007 (H4N6)	2007-12-06	KX978511	KX977631
A/Bewicks swan/Netherlands/3/2006 (H1N1)	2006-11-17	KX978266	KX977791
A/Bewicks swan/Netherlands/3/2007 (H4N6)	2007-12-06	KX978846	KX979193
A/Bewicks swan/Netherlands/4/2006 (H9N2)	2006-11-17	KX977700	KX979422
A/Bewicks swan/Netherlands/5/2008 (H5N2)	2008-12-17	KX979015	KX978975
A/Bewicks swan/Netherlands/6/2006 (H1N1)	2006-11-22	KX978574	KX978892
A/Bewicks swan/Netherlands/7/2008 (H7N1)	2008-12-24	KX978000	KX978951
A/Bewicks swan/Netherlands/8/2009 (H6N8)	2008-01-04	KX977884	KX978281
A/Brent goose/Netherlands/1/2006 (H1N1)	2006-01-10	KX979392	KX979523
A/Dunlin/Netherlands/2/2007 (H3N8)	2007-07-31	KX978538	KX979291
A/Dunlin/Netherlands/3/2007 (H3N8)	2007-07-31	KX979366	KX978457
A/Eurasian teal/Netherlands/1/2008 (H6N1)	2008-09-22	KX978922	KX978742
A/Eurasian teal/Netherlands/1/2011 (H3N8)	2011-10-15	KX979458	KX979211
A/Eurasian teal/Netherlands/3/2008 (H6N1)	2008-09-22	KX978950	KX979001
A/Eurasian wigeon/Netherlands/1/2006 (H6N2)	2007-12-21	KX979500	KX978832
A/Eurasian wigeon/Netherlands/1/2008 (H6N1)	2008-09-22	KX978248	KX978405
A/Eurasian wigeon/Netherlands/1/2009 (H5N2)	2009-09-11	KX979575	KX977926
A/Eurasian wigeon/Netherlands/1/2010 (H5N2)	2010-09-27	KX977950	KX978397
A/Eurasian wigeon/Netherlands/2/2007 (H1N1)	2007-10-20	KX978381	KX977776
A/Eurasian wigeon/Netherlands/2/2008 (H7N4)	2008-12-29	KX978318	KX977640
A/Eurasian wigeon/Netherlands/6/2007 (H1N1)	2007-11-15	KX979129	KX977745
A/Gadwall duck/Netherlands/1/2011 (H6N2)	2011-11-04	KX978389	KX977644
A/Gadwall duck/Netherlands/2/2006 (H9N2)	2006-01-01*	KX978145	KX979196
A/Gadwall duck/Netherlands/3/2006 (H3N8)	2006-09-03	KX978829	KX979428
A/Greater white-fronted goose/Netherlands/1/2006 (H6N2)	2006-01-14	KX979150	KX978480
A/Greater white-fronted goose/Netherlands/1/2007 (H1N1)	2007-01-23	KX977711	KX977666
A/Greater white-fronted goose/Netherlands/1/2009 (H6N8)	2009-01-20	KX978830	KX979241
A/Greater white-fronted goose/Netherlands/1/2010 (H6N1)	2010-01-20	KX977696	KX978928
A/Greater white-fronted goose/Netherlands/1/2011 (H6N8)	2011-01-04	KX977775	KX978983
A/Greater white-fronted goose/Netherlands/1/2012 (H1N1)	2012-11-20	KX978558	KX978251
A/Greater white-fronted goose/Netherlands/11/2009 (H6N1)	2009-12-09	KX977968	KX978797
A/Greater white-fronted goose/Netherlands/2/2007 (H6N8)	2007-12-12	KX979168	KX978521
A/Greater white-fronted goose/Netherlands/2/2008 (H6N8)	2008-01-22	KX979494	KX978669
A/Greater white-fronted goose/Netherlands/2/2009 (H5N3)	2009-12-14	KX977974	KX979382
A/Greater white-fronted goose/Netherlands/2/2010 (H5N2)	2010-01-29	KX978688	KX978932
A/Greater white-fronted goose/Netherlands/2/2011 (H6N2)	2011-01-13	KX977613	KX978753
A/Greater white-fronted goose/Netherlands/3/2006 (H6N8)	2006-01-01*	KX979190	KX979264

A/Greater white-fronted goose/Netherlands/3/2007 (H6N5)	2007-12-18	KX978034	KX978798
A/Greater white-fronted goose/Netherlands/3/2011 (H6N2)	2011-01-11	KX978988	KX978364
A/Greater white-fronted goose/Netherlands/4/2006 (H6N8)	2006-01-01*	KX978498	KX979449
A/Greater white-fronted goose/Netherlands/4/2008 (H6N8)	2008-01-22	KX979397	KX979084
A/Greater white-fronted goose/Netherlands/4/2009 (H5N2)	2009-02-21	KX978287	KX978719
A/Greater white-fronted goose/Netherlands/4/2010 (H6N8)	2010-02-04	KX978377	KX978184
A/Greater white-fronted goose/Netherlands/4/2011 (H1N1)	2011-01-17	KX978591	KX978181
A/Greater white-fronted goose/Netherlands/5/2008 (H5N2)	2008-12-30	KX979073	KX978387
A/Greater white-fronted goose/Netherlands/5/2010 (H5N3)	2010-11-13	KX978851	KX979439
A/Greater white-fronted goose/Netherlands/6/2008 (H5N2)	2008-12-30	KX978329	KX978609
A/Greater white-fronted goose/Netherlands/6/2009 (H6N8)	2009-01-04	KX978349	KX977708
A/Greater white-fronted goose/Netherlands/6/2010 (H6N2)	2010-11-23	KX978734	KX978786
A/Greater white-fronted goose/Netherlands/6/2011 (H6N8)	2011-12-14	KX978828	KX977722
A/Greater white-fronted goose/Netherlands/8/2009 (H6N2)	2009-12-30	KX978319	KX978626
A/Greater white-fronted goose/Netherlands/9/2009 (H6N2)	2009-12-30	KX978339	KX978180
A/Mallard duck/Netherlands/1/2007 (H10N7)	2007-01-01*	KX978192	KX977605
A/Mallard duck/Netherlands/1/2008 (H5N3)	2008-01-30	KX977798	KX978667
A/Mallard duck/Netherlands/1/2009 (H10N7)	2009-02-04	KX977889	KX979271
A/Mallard duck/Netherlands/1/2011 (H7N7)	2011-02-23	KX979524	KX978358
A/Mallard duck/Netherlands/1/2012 (H10N7)	2012-01-14	KX979240	KX978542
A/Mallard duck/Netherlands/1/2013 (H7N7)	2013-01-26	KX978524	KX978579
A/Mallard duck/Netherlands/1/2014 (H10N7)	2014-02-17	KX979229	KX978673
A/Mallard duck/Netherlands/10/2012 (H6N1)	2012-09-05	KX979411	KX978507
A/Mallard duck/Netherlands/12/2006 (H5N2)	2006-09-16	KX977669	KX979415
A/Mallard duck/Netherlands/12/2009 (H11N9)	2009-09-02	KX978958	KX978900
A/Mallard duck/Netherlands/12/2012 (H6N1)	2012-10-18	KX978330	KX979308
A/Mallard duck/Netherlands/13/2007 (H6N5)	2007-11-01	KX977641	KX977833
A/Mallard duck/Netherlands/13/2012 (H3N8)	2012-10-01	KX979517	KX979121
A/Mallard duck/Netherlands/13/2013 (H7N7)	2013-12-10	KX979399	KX978080
A/Mallard duck/Netherlands/14/2006 (H8N4)	2006-01-01*	KX977927	KX979046
A/Mallard duck/Netherlands/15/2007 (H6N8)	2007-11-09	KX979354	KX979328
A/Mallard duck/Netherlands/15/2009 (H6N8)	2009-10-02	KX978489	KX978378
A/Mallard duck/Netherlands/15/2011 (H6N8)	2011-09-14	KX978593	KX978920
A/Mallard duck/Netherlands/16/2006 (H10N7)	2006-01-14	KX978236	KX978605
A/Mallard duck/Netherlands/16/2007 (H6N8)	2007-11-15	KX978196	KX978905
A/Mallard duck/Netherlands/16/2009 (H1N1)	2009-09-30	KX978081	KX977672
A/Mallard duck/Netherlands/17/2009 (H6N8)	2009-10-06	KX978322	KX978549
A/Mallard duck/Netherlands/17/2011 (H3N8)	2011-09-20	KX978036	KX978560
A/Mallard duck/Netherlands/18/2007 (H4N6)	2007-11-22	KX979234	KX978376
A/Mallard duck/Netherlands/18/2009 (H6N2)	2009-10-08	KX978564	KX979049
A/Mallard duck/Netherlands/18/2010 (H6N8)	2010-09-03	KX979113	KX977957
A/Mallard duck/Netherlands/19/2007 (H6N2)	2007-12-07	KX978966	KX978008
A/Mallard duck/Netherlands/19/2009 (H5N3)	2009-10-26	KX978032	KX978963
A/Mallard duck/Netherlands/19/2012 (H4N6)	2012-09-07	KX979134	KX978671
A/Mallard duck/Netherlands/2/2007 (H10N7)	2007-01-01*	KX978494	KX979021
A/Mallard duck/Netherlands/2/2008 (H10N7)	2008-01-07	KX978964	KX978237
A/Mallard duck/Netherlands/2/2009 (H7N7)	2009-01-09	KX978114	KX978763
A/Mallard duck/Netherlands/2/2011 (H10N7)	2011-03-08	KX978624	KX979161
A/Mallard duck/Netherlands/2/2015 (H7N7)	2015-01-16	KX979185	KX978353
A/Mallard duck/Netherlands/20/2009 (H5N3)	2009-10-26	KX978388	KX977757
A/Mallard duck/Netherlands/20/2011 (H6N8)	2011-09-24	KX978022	KX979447

A/Mallard duck/Netherlands/22/2010 (H10N7)	2010-09-07	KX978006	KX978632
A/Mallard duck/Netherlands/23/2006 (H1N1)	2006-08-25	KX979325	KX978811
A/Mallard duck/Netherlands/23/2012 (H11N9)	2012-09-27	KX977778	KX977684
A/Mallard duck/Netherlands/24/2009 (H6N1)	2009-12-01	KX978844	KX978238
A/Mallard duck/Netherlands/24/2013 (H4N6)	2013-09-16	KX978385	KX978890
A/Mallard duck/Netherlands/26/2010 (H4N6)	2010-09-13	KX979423	KX979292
A/Mallard duck/Netherlands/26/2011 (H11N9)	2011-10-10	KX979452	KX978692
A/Mallard duck/Netherlands/27/2009 (H4N6)	2009-12-21	KX978295	KX978169
A/Mallard duck/Netherlands/28/2008 (H5N6)	2008-09-22	KX977852	KX978060
A/Mallard duck/Netherlands/28/2009 (H1N1)	2009-12-21	KX977807	KX978631
A/Mallard duck/Netherlands/28/2010 (H5N2)	2010-09-17	KX979224	KX977947
A/Mallard duck/Netherlands/29/2008 (H11N9)	2008-09-22	KX978267	KX978971
A/Mallard duck/Netherlands/29/2009 (H11N9)	2009-12-21	KX977705	KX977840
A/Mallard duck/Netherlands/3/2008 (H6N2)	2008-01-07	KX977797	KX978336
A/Mallard duck/Netherlands/3/2015 (H7N7)	2015-01-24	KX978067	KX979143
A/Mallard duck/Netherlands/30/2008 (H5N3)	2008-09-23	KX978877	KX978452
A/Mallard duck/Netherlands/30/2010 (H3N8)	2010-09-21	KX978097	KX979363
A/Mallard duck/Netherlands/30/2011 (H6N4)	2011-10-19	KX979356	KX979148
A/Mallard duck/Netherlands/30/2014 (H4N6)	2014-07-21	KX978886	KX979350
A/Mallard duck/Netherlands/31/2008 (H6N1)	2008-09-22	KX978655	KX978400
A/Mallard duck/Netherlands/31/2012 (H7N1)	2012-01-10	KX977922	KX978711
A/Mallard duck/Netherlands/31/2013 (H10N7)	2013-05-20	KX978193	KX977867
A/Mallard duck/Netherlands/32/2011 (H5N2)	2011-10-20	KX979009	KX978469
A/Mallard duck/Netherlands/32/2013 (H10N7)	2013-08-05	KX977924	KX978875
A/Mallard duck/Netherlands/33/2014 (H10N7)	2014-12-16	KX978314	KX979578
A/Mallard duck/Netherlands/34/2006 (H3N8)	2006-09-11	KX979215	KX979577
A/Mallard duck/Netherlands/36/2006 (H5N3)	2006-09-11	KX979003	KX979553
A/Mallard duck/Netherlands/36/2008 (H11N9)	2008-09-23	KX978075	KX978903
A/Mallard duck/Netherlands/37/2011 (H1N1)	2011-12-08	KX977882	KX979536
A/Mallard duck/Netherlands/40/2006 (H6N8)	2006-09-16	KX977839	KX978188
A/Mallard duck/Netherlands/43/2006 (H5N2)	2006-09-18	KX979501	KX978545
A/Mallard duck/Netherlands/43/2011 (H7N1)	2011-12-24	KX977677	KX978462
A/Mallard duck/Netherlands/44/2011 (H7N1)	2011-12-24	KX978740	KX977735
A/Mallard duck/Netherlands/47/2010 (H10N7)	2010-11-26	KX978640	KX977904
A/Mallard duck/Netherlands/5/2008 (H6N8)	2008-09-10	KX979551	KX977649
A/Mallard duck/Netherlands/5/2009 (H5N2)	2009-02-02	KX977767	KX978896
A/Mallard duck/Netherlands/5/2012 (H10N7)	2012-05-12	KX978848	KX979065
A/Mallard duck/Netherlands/5/2013 (H1N1)	2013-09-16	KX979268	KX978982
A/Mallard duck/Netherlands/51/2010 (H1N1)	2010-12-03	KX978723	KX978328
A/Mallard duck/Netherlands/52/2008 (H5N2)	2008-10-09	KX979499	KX977925
A/Mallard duck/Netherlands/53/2010 (H1N1)	2010-12-08	KX978255	KX977899
A/Mallard duck/Netherlands/55/2008 (H4N6)	2008-10-06	KX978110	KX978178
A/Mallard duck/Netherlands/6/2006 (H4N6)	2006-06-03	KX977697	KX979028
A/Mallard duck/Netherlands/6/2013 (H3N8)	2013-10-01	KX979335	KX979409
A/Mallard duck/Netherlands/6/2015 (H10N7)	2015-03-04	KX978577	KX978040
A/Mallard duck/Netherlands/60/2008 (H7N1)	2008-10-15	KX978337	KX979011
A/Mallard duck/Netherlands/64/2006 (H1N1)	2006-09-22	KX978552	KX977762
A/Mallard duck/Netherlands/67/2008 (H10N7)	2008-12-13	KX979437	KX977963
A/Mallard duck/Netherlands/7/2008 (H3N8)	2008-08-22	KX978132	KX978054
A/Mallard duck/Netherlands/7/2009 (H7N7)	2009-02-02	KX977692	KX978598
A/Mallard duck/Netherlands/7/2011 (H4N6)	2011-08-15	KX978670	KX977749

A/Mallard duck/Netherlands/7/2012 (H1N1)	2012-07-09	KX978074	KX978447
A/Mallard duck/Netherlands/7/2014 (H6N2)	2014-10-01	KX978776	KX979466
A/Mallard duck/Netherlands/76/2008 (H6N8)	2008-11-27	KX978130	KX978245
A/Mallard duck/Netherlands/77/2008 (H7N1)	2008-12-29	KX977881	KX978814
A/Mallard duck/Netherlands/78/2006 (H6N8)	2006-10-21	KX978159	KX977724
A/Mallard duck/Netherlands/8/2008 (H6N1)	2008-09-27	KX978779	KX977984
A/Mallard duck/Netherlands/8/2012 (H1N1)	2012-10-23	KX979169	KX977770
A/Mallard duck/Netherlands/82/2008 (H7N7)	2008-12-17	KX979315	KX979225
A/Mallard duck/Netherlands/9/2013 (H6N8)	2013-10-15	KX979493	KX979138
A/Mute swan/Netherlands/2/2006 (H4N6)	2006-01-01*	KX978073	KX979448
A/Mute swan/Netherlands/3/2006 (H4N6)	2006-01-01*	KX977958	KX978909
A/Turnstone/Netherlands/1/2010 (H3N8)	2010-10-09	KX979124	KX977713
A/Turnstone/Netherlands/2/2007 (H3N8)	2007-07-31	KX979144	KX978279
A/Turnstone/Netherlands/3/2008 (H3N8)	2008-10-31	KX978665	KX979101
A/Turnstone/Netherlands/4/2008 (H3N8)	2008-11-01	KX978661	KX979097

* date unknown, set to 1st of January

Supplementary Table S2. GISAID accession numbers of poultry virus sequences.

GISAID's EpiFlu database accession numbers of hemagglutinin (HA) and neuraminidase (NA) gene segment sequences of low pathogenic avian influenza (LPAI) viruses from poultry, detected as part of the national avian influenza (AI) surveillance program in the Netherlands, January 2006-September 2016.

Isolate name	Collection date	GISAID HA segment ID	GISAID NA segment ID
A/Chicken/Netherlands/06022003/2006 (H7N7)	2006-08-01	EPI1229818	EPI1229817
A/Chicken/Netherlands/09006942/2009 (H10N7)	2009-04-15	EPI1229826	EPI1229825
A/Chicken/Netherlands/10007882/2010 (H7N4)	2010-05-14	EPI1229834	EPI1229833
A/Chicken/Netherlands/10008427/2010 (H10N7)	2010-05-20	EPI966037	EPI966050
A/Chicken/Netherlands/10009401/2010 (H8N4)	2010-06-04	EPI1229842	EPI1229841
A/Chicken/Netherlands/10010413/2010 (H6N1)	2010-06-21	EPI1229850	EPI1229849
A/Chicken/Netherlands/10012103/2010 (H6N1)	2010-07-19	EPI1229858	EPI1229857
A/Chicken/Netherlands/10020245/2010 (H9N2)	2010-12-10	EPI1229866	EPI1229865
A/Chicken/Netherlands/11004004/2011 (H8N4)	2011-03-09	EPI1229874	EPI1229873
A/Chicken/Netherlands/11004875/2011 (H7N1)	2011-03-22	EPI1229882	EPI1229881
A/Chicken/Netherlands/11008325/2011 (H8N4)	2011-05-10	EPI1229890	EPI1229889
A/Chicken/Netherlands/11008327/2011 (H7N7)	2011-05-12	EPI1230758	EPI1230760
A/Chicken/Netherlands/11009919/2011 (H1N1)	2011-05-30	EPI1229898	EPI1229897
A/Chicken/Netherlands/11011392/2011 (H7N7)	2011-06-22	EPI1229906	EPI1229905
A/Chicken/Netherlands/12002495-001-005/2012 (H10N9)	2012-02-06	EPI1229914	EPI1229913
A/Chicken/Netherlands/12014794/2012 (H7N7)	2012-08-09	EPI1229922	EPI1229921
A/Chicken/Netherlands/13003601/2013 (H7N7)	2013-03-12	EPI1229930	EPI1229929
A/Chicken/Netherlands/13003983/2013 (H7N7)	2013-03-18	EPI1229938	EPI1229937
A/Chicken/Netherlands/13015884/2013 (H5N3)	2013-11-29	EPI1229946	EPI1229945
A/Chicken/Netherlands/13016263-031-035/2013 (H5N3)	2013-12-10	EPI1229954	EPI1229953
A/Chicken/Netherlands/14002541/2014 (H5N1)	2014-02-25	EPI1229962	EPI1229961
A/Chicken/Netherlands/14003005/2014 (H2N7)	2014-03-05	EPI1229970	EPI1229969
A/Chicken/Netherlands/14003323/2014 (H5N2)	2014-03-12	EPI1229978	EPI1229977
A/Chicken/Netherlands/14004070/2014 (H9N1)	2014-03-26	EPI1229986	EPI1229985
A/Chicken/Netherlands/15005968-056060/2015 (H5N2)	2015-04-02	EPI1230063	EPI1230062
A/Chicken/Netherlands/15007212/2015 (H10N7)	2015-04-28	EPI774514	EPI774516
A/Chicken/Netherlands/16007311-037041/2016 (H7N9)	2016-06-08	EPI773765	EPI773767
A/Chicken/Netherlands/16010778-021-025/2016 (H2N3)	2016-08-30	EPI1230079	EPI1230078
A/Chicken/NL-Barneveld/15004745-001-005/2015 (H7N7)	2015-03-11	EPI629344	EPI629341
A/Duck/Netherlands/06027358/2006 (H3N8)	2006-09-27	EPI1230087	EPI1230086
A/Duck/Netherlands/14015610/2014 (H6N2)	2014-11-17	EPI1230095	EPI1230094
A/Duck/Netherlands/14016168/2014 (H6N8)	2014-11-25	EPI1230103	EPI1230102
A/Duck/Netherlands/14016396/2014 (H6N2)	2014-11-25	EPI1230111	EPI1230110
A/Turkey/Netherlands/06001571/2006 (H6N5)	2006-01-24	EPI1229994	EPI1229993
A/Turkey/Netherlands/07014290/2007 (H1N5)	2007-05-31	EPI1230002	EPI1230001
A/Turkey/Netherlands/07016245/2007 (H1N5)	2007-06-22	EPI1230010	EPI1230009
A/Turkey/Netherlands/09006938/2009 (H10N7)	2009-04-14	EPI1230018	EPI1230017
A/Turkey/Netherlands/11011530/2011 (H7N7)	2011-06-25	EPI1230026	EPI1230025
A/Turkey/Netherlands/11015452/2011 (H9N2)	2011-08-31	EPI1230034	EPI1230033
A/Turkey/Netherlands/12004763-001-004/2012 (H10N9)	2012-03-05	EPI1230042	EPI1230041
A/Turkey/Netherlands/12005615/2012 (H10N9)	2012-03-13	EPI1230050	EPI1230049
A/Turkey/Netherlands/13001007/2013 (H8N4)	2013-01-18	EPI1230127	EPI1230126

Supplementary Table S3. Collection and subtyping of wild bird samples.

Number of wild birds sampled and number of subtyped cases of low pathogenic avian influenza (LPAI) virus detections per wild bird species as part of the national avian influenza (AI) surveillance program in the Netherlands, January 2006-September 2016. A case is considered subtyped if the hemagglutinin (HA) or neuraminidase (NA) subtype of the virus is determined.

Order	Group	Species	Latin name	Number of birds sampled	Number of subtyped cases
Anseriformes	Ducks	Mallard	*Anas platyrhynchos*	46939	446
		Eurasian Wigeon	*Anas penelope*	10039	22
		Gadwall	*Anas strepera*	1317	5
		Common Teal	*Anas crecca*	1209	8
		Northern Pintail	*Anas acuta*	372	0
		Northern Shoveler	*Anas clypeata*	355	1
		Common Eider	*Somateria mollissima*	99	2
		Other ducks		312	0
	Geese	White-fronted Goose	*Anser albifrons*	12001	31
		Barnacle Goose	*Branta leucopsis*	4933	8
		Greylag Goose	*Anser anser*	1924	1
		Egyptian Goose	*Alopochen aegyptiacus*	1159	0
		Bean Goose	*Anser fabalis*	1095	3
		Brent Goose	*Branta bernicla*	858	1
		Canada Goose	*Branta canadensis*	326	0
		Pink-footed Goose	*Anser brachyrhynchus*	206	1
		Other geese		110	0
	Swans	Mute Swan	*Cygnus olor*	2126	3
		Bewick's Swan	*Cygnus bewickii*	250	14
		Other swans		46	0
Charadriiformes	Gulls	Black-headed Gull	*Chroicocephalus ridibundus*	14170	401
		Common Gull	*Larus canus*	2087	0
		Herring Gull	*Larus argentatus*	1024	10
		Lesser Black-backed Gull	*Larus fuscus*	894	4
		Mediterranean Gull	*Larus Melanocephalus*	207	1
		Great Black-backed Gull	*Larus marinus*	31	1
		Other gulls		51	0
	Waders	Turnstone	*Arenaria interpres*	911	14
		Dunlin	*Calidris alpina*	654	4
		Oystercatcher	*Haematopus ostralegus*	724	0
		Red Knot	*Calidris canutus*	163	0
		Other waders		177	0
	Other *Charadriiformes*			351	0
Other				2172	0
Unknown				1822	0
Total				111114	981

Supplementary Table S4. Collection and subtyping of poultry samples.

Number of serologically tested poultry farms and number of subtyped cases of low pathogenic avian influenza (LPAI) virus detections per poultry type as part of the national avian influenza (AI) surveillance program in the Netherlands, January 2006-September 2016. A case is considered if the hemagglutinin (HA) or neuraminidase (NA) subtype of the virus or the subtype-specificity of the influenza virus-specific antibodies is determined.

Poultry type		Housing system	Number of farms tested	Number of subtyped cases
Chicken	Layer	Indoor	18883	20
		Outdoor	11797	120
		Unknown		8
	Broiler		7309	9
	Unknown			10
Turkey			2269	33
Duck			889	13
Unknown			662	7
Total			41769	220

Supplementary Table S5. Poultry viruses and their most identical wild bird virus.

Low pathogenic avian influenza (LPAI) viruses isolated from poultry, detected as part of the national avian influenza (AI) surveillance program in the Netherlands, January 2006-September 2016, with their genetically most identical wild bird virus based on the hemagglutinin (HA) and neuraminidase (NA) gene segment sequences as determined by BLAST. The time interval between collection days and the percentage of nucleotide (nt) sequence identity between the poultry virus and the most identical wild bird virus are shown. We gratefully acknowledge the authors, originating and submitting laboratories of the sequences from GISAID's EpiFlu database [28] on which this research is based. All submitters of data may be contacted directly via the GISAID website (http://www.gisaid.org).

Year	Poultry virus	Location poultry virus (province – city)	Seg-ment	Most identical wild bird virus	Location wild bird virus (continent – country – province)	Time interval (days)	Sequence identity (%)
2006	A/Turkey/Netherlands/06001571/2006 (H6N5)	Noord Brabant - Dinteloord	HA	A/Greater white-fronted goose/Netherlands/ 1/2006 (H6N2)	EU - NL - Noord Brabant	10	99.5%
			NA	A/Mallard/Sweden/343/2002 (H12N5)	EU - Sweden	1181	98.7%
	A/Chicken/Netherlands/06022003/2006 (H7N7)	Gelderland - Voorthuizen	HA	A/Mallard Duck/Netherlands/ 60/2008 (H7N1)	EU - NL - Noord Holland	-806	99.5%
			NA	A/Mallard/Sweden/5927/2005 (H7N7)	EU - Sweden	251	98.7%
				A/Mallard/Sweden/5944/2005 (H7N7)	EU - Sweden	248	
				A/Mallard/Sweden/95/2005 (H7N7)	EU - Sweden	577	
	A/Duck/Netherlands/06027358/2006 (H3N8)	Gelderland - Nijmegen	HA	A/Mallard Duck/Netherlands/ 60/2006 (H3N8)	EU - NL - Noord Holland	9	99.8%
			NA	A/Mallard Duck/Netherlands/ 60/2006 (H3N8)	EU - NL - Noord Holland	9	99.8%
2007	A/Turkey/Netherlands/07014290/2007 (H1N5)	Limburg - Nederweert	HA	A/Bewicks swan/Netherlands/ 1/2007 (H1N5)	EU - NL - Friesland	150	98.9%
			NA	A/Black-headed gull/Netherlands/ 1/2006 (H4N5)	EU - NL - Gelderland	515	98.5%
	A/Turkey/Netherlands/07016245/2007 (H1N5)	Limburg - Weert	HA	A/Bewicks swan/Netherlands/ 1/2007 (H1N5)	EU - NL - Friesland	172	99.0%
			NA	A/Black-headed gull/Netherlands/ 1/2006 (H4N5)	EU - NL - Gelderland	537	98.5%
2009	A/Turkey/Netherlands/09006938/2009 (H10N7)	Noord Brabant - Deurne	HA	A/Mallard/Sweden/64476/2007 (H10N4)	EU - Sweden	677	98.9%
			NA	A/Anas_platyrhynchos/Camargue/091863/09 (H10N7)	EU - France	-36	99.8%
				A/Mallard Duck/Netherlands/ 82/2008 (H7N7)	EU - NL - Zuid Holland	118	
	A/Chicken/Netherlands/09006942/2009 (H10N7)	Noord Brabant - Deurne	HA	A/Mallard/Sweden/64476/2007 (H10N4)	EU - Sweden	678	98.8%
				A/Mallard/Sweden/93475/2009 (H10N6)	EU - Sweden	-130	
			NA	A/Anas_platyrhynchos/Camargue/091863/09 (H10N7)	EU - France	-35	99.8%
				A/Mallard Duck/Netherlands/ 82/2008 (H7N7)	EU - NL - Zuid Holland	119	
2010	A/Chicken/Netherlands/10007882/2010 (H7N4)	Noord Brabant - Deurne	HA	A/Mallard Duck/Netherlands/ 60/2008 (H7N1)	EU - NL - Noord Holland	576	99.1%
			NA	A/Teal/Chany/7119/2008 (H15N4)	EU - Russia	622	99.1%
	A/Chicken/Netherlands/10008427/2010 (H10N7)	Friesland – Drachtstercompagnie	HA	A/Mallard/Netherlands/ 67/2008 (H10N7)	EU - NL - Friesland	523	99.0%
			NA	A/Mallard/Netherlands/ 67/2008 (H10N7)	EU - NL - Friesland	523	99.0%
				A/Mallard/Netherlands/ 82/2008 (H10N7)	EU - NL - Friesland	519	
				A/Mallard/Netherlands/ 1/2009 (H10N7)	EU - NL - Friesland	470	
	A/Chicken/Netherlands/10009401/2010 (H8N4)	Friesland - Hiaure	HA	A/Mallard/Sweden/99377/2009 (H8N4)	EU - Sweden	274	98.5%
			NA	A/Teal/Chany/7119/2008 (H15N4)	EU - Russia	643	99.2%

	A/Chicken/Netherlands/10010413/2010 (H6N1)	Friesland - Idsegahuizum	HA	A/Mallard Duck/Netherlands/ 18/2010 (H6N8)	EU - NL - Zuid Holland	-74	98.8%
			NA	A/Mallard/Bavaria/185-8/2008 (H1N1)	EU - Germany	637	99.2%
	A/Chicken/Netherlands/10012103/2010 (H6N1)	Friesland - Parrega	HA	A/Mallard Duck/Netherlands/ 18/2010 (H6N8)	EU - NL - Zuid Holland	-46	98.8%
			NA	A/Mallard/Bavaria/185-8/2008 (H1N1)	EU - Germany	665	99.1%
	A/Chicken/Netherlands/10020245/2010 (H9N2)	Zuid Holland - Pijnacker	HA	A/Bewicks swan/Netherlands/ 4/2006 (H9N2)	EU - NL - Noord Holland	1484	96.6%
			NA	A/Mallard Duck/Netherlands/ 53/2006 (H4N2)	EU - NL - Noord Holland	1544	98.0%
				A/Mallard/Netherlands/ 7/2007 (H4N2)	EU - NL - Zuid Holland	1439	
2011	A/Chicken/Netherlands/11004004/2011 (H8N4)	Utrecht - Vreeland	HA	A/Common Teal/Netherlands/ 12002960/2012 (H8N4)	EU - NL - Noord Holland	-359	99.2%
			NA	A/Common Teal/Netherlands/ 12002960/2012 (H8N4)	EU - NL - Noord Holland	-359	99.5%
	A/Chicken/Netherlands/11004875/2011 (H7N1)	Zeeland - Schore	HA	A/Swan/Czech Republic/5416/2011 (H7N7)	EU - Czech Republic	-15	99.5%
			NA	A/Mallard Duck/Netherlands/ 51/2010 (H1N1)	EU - NL - Zuid Holland	109	99.6%
	A/Chicken/Netherlands/11008325/2011 (H8N4)	Gelderland - Lunteren	HA	A/Mallard/Sweden/101165/2009 (H8N4)	EU - Sweden	551	99.2%
			NA	A/Mallard/Sweden/133546/2011 (H10N4)	EU - Sweden	-197	99.4%
	A/Chicken/Netherlands/11008327/2011 (H7N7)	Gelderland - Kootwijkerbroek	HA	A/Mallard Duck/Netherlands/ 1/2011 (H7N7)	EU - NL - Noord Holland	78	99.4%
			NA	A/Mallard Duck/Netherlands/ 1/2011 (H7N7)	EU - NL - Noord Holland	78	98.8%
	A/Chicken/Netherlands/11009919/2011 (H1N1)	Zuid Holland - Stolwijk	HA	A/Greater white-fronted goose/Netherlands/ 4/2011 (H1N1)	EU - NL - Noord Brabant	133	98.8%
			NA	A/Greater white-fronted goose/Netherlands/ 4/2011 (H1N1)	EU - NL - Noord Brabant	133	99.9%
	A/Chicken/Netherlands/11011392/2011 (H7N7)<br>A/Chicken/Netherlands/11011326/2011 (H7N7)*	Flevoland - Creil	HA	A/Mallard Duck/Netherlands/ 1/2011 (H7N7)	EU - NL - Noord Holland	120	99.3%
			NA	A/Mallard Duck/Netherlands/ 1/2011 (H7N7)	EU - NL - Noord Holland	120	98.8%
	A/Turkey/Netherlands/11011530/2011 (H7N7)	Flevoland - Creil	HA	A/Mallard Duck/Netherlands/ 1/2011 (H7N7)	EU - NL - Noord Holland	122	99.3%
			NA	A/Mallard Duck/Netherlands/ 1/2011 (H7N7)	EU - NL - Noord Holland	122	98.7%
	A/Turkey/Netherlands/11015452/2011 (H9N2)	Noord Brabant - Deurne	HA	A/Anas platyrhynchos/Belgium/24311pcs5/2012 (H9N2)	EU - Belgium	-375	99.2%
			NA	A/Mallard Duck/Netherlands/ 16/2012 (H3N2)	EU - NL - Zuid Holland	-352	99.1%
2012	A/Chicken/Netherlands/12002495-001-005/2012 (H10N9)	Noord Brabant - Heusden	HA	A/Mallard/Sweden/105186/2009 (H10N1)	EU - Sweden	800	98.2%
				A/Mallard/Sweden/105254/2009 (H10N1)	EU - Sweden	799	
				A/Mallard/Sweden/105259/2009 (H10N1)	EU - Sweden	799	
				A/Mallard/Sweden/105364/2009 (H10N1)	EU - Sweden	798	
				A/Mallard/Sweden/105365/2009 (H10N1)	EU - Sweden	798	
				A/Mallard/Sweden/105474/2009 (H10N1)	EU - Sweden	796	
				A/Mallard/Sweden/107688/2009 (H10N1)	EU - Sweden	794	
			NA	A/Mallard/Sweden/100878/2009 (H11N9)	EU - Sweden	827	99.0%
				A/Mallard/Sweden/101011/2009 (H11N9)	EU - Sweden	825	
				A/Mallard/Sweden/50980/2006 (H11N9)	EU - Sweden	1946	
	A/Turkey/Netherlands/12004763-001-004/2012 (H10N9)	Limburg - Ospel	HA	A/Mallard/Sweden/105186/2009 (H10N1)	EU - Sweden	828	98.2%
				A/Mallard/Sweden/105254/2009 (H10N1)	EU - Sweden	827	
				A/Mallard/Sweden/105259/2009 (H10N1)	EU - Sweden	827	
				A/Mallard/Sweden/105364/2009 (H10N1)	EU - Sweden	826	
				A/Mallard/Sweden/105365/2009 (H10N1)	EU - Sweden	826	

				A/Mallard/Sweden/105474/2009 (H10N1)	EU - Sweden	824	
				A/Mallard/Sweden/107688/2009 (H10N1)	EU - Sweden	822	
			NA	A/Mallard/Sweden/100878/2009 (H11N9)	EU - Sweden	855	98.9%
				A/Mallard/Sweden/101011/2009 (H11N9)	EU - Sweden	853	
				A/Mallard/Sweden/50980/2006 (H11N9)	EU - Sweden	1974	
	A/Turkey/Netherlands/12005615/2012 (H10N9)	Limburg - Nederweert	HA	A/Mallard/Sweden/105186/2009 (H10N1)	EU - Sweden	836	98.2%
				A/Mallard/Sweden/105254/2009 (H10N1)	EU - Sweden	835	
				A/Mallard/Sweden/105259/2009 (H10N1)	EU - Sweden	835	
				A/Mallard/Sweden/105364/2009 (H10N1)	EU - Sweden	834	
				A/Mallard/Sweden/105365/2009 (H10N1)	EU - Sweden	834	
				A/Mallard/Sweden/105474/2009 (H10N1)	EU - Sweden	832	
				A/Mallard/Sweden/107688/2009 (H10N1)	EU - Sweden	830	
			NA	A/Mallard/Sweden/100878/2009 (H11N9)	EU - Sweden	863	98.8%
				A/Mallard/Sweden/101011/2009 (H11N9)	EU - Sweden	861	
				A/Mallard/Sweden/50980/2006 (H11N9)	EU - Sweden	1982	
	A/Chicken/Netherlands/12014794/2012 (H7N7)	Utrecht - Hagestein	HA	A/Anas platyrhynchos/Belgium/23852cls33/2012 (H7N7)	EU - Belgium	-34	99.4%
			NA	A/Swan/Czech Republic/5416/2011 (H7N7)	EU - Czech Republic	491	98.7%
2013	A/Turkey/Netherlands/13001007/2013 (H8N4)	Noord Brabant - Helmond	HA	A/Mallard/Sweden/101165/2009 (H8N4)	EU - Sweden	1170	98.2%
			NA	A/Mallard/Sweden/99377/2009 (H8N4)	EU - Sweden	1233	98.4%
	A/Chicken/Netherlands/13003601/2013 (H7N7)	Gelderland - Lochem	HA	A/Anas platyrhynchos/Belgium/23852cls33/2012 (H7N7)	EU - Belgium	181	99.2%
				A/Swan/Czech Republic/5416/2011 (H7N7)	EU - Czech Republic	706	
			NA	A/Anas platyrhynchos/Belgium/23852cls33/2012 (H7N7)	EU - Belgium	181	99.2%
	A/Chicken/Netherlands/13003983/2013 (H7N7)	Flevoland - Zeewolde	HA	A/Swan/Czech Republic/5416/2011 (H7N7)	EU - Czech Republic	712	98.9%
			NA	A/Mallard Duck/Netherlands/ 5/2012 (H10N7)	EU - NL - Zuid Holland	310	98.7%
	A/Chicken/Netherlands/13015884/2013 (H5N3)	Groningen - Sint Annen	HA	A/Razorbill/Scotland/7343/14 (H5Nx)	EU - United Kingdom	-94	98.5%
			NA	A/Mallard/Netherlands/ 29/2010 (H2N3)	EU - NL - Noord Holland	1428	98.7%
				A/Mallard/Sweden/101900/2009 (H4N3)	EU - Sweden	1476	
	A/Chicken/Netherlands/13016263-031-035/2013 (H5N3)	Groningen - Scheemda	HA	A/Razorbill/Scotland/7343/14 (H5Nx)	EU - United Kingdom	-83	98.5%
			NA	A/Mallard/Netherlands/ 29/2010 (H2N3)	EU - NL - Noord Holland	1439	98.8%
				A/Mallard/Sweden/101900/2009 (H4N3)	EU - Sweden	1487	
2014	A/Chicken/Netherlands/14002541/2014 (H5N1)	Flevoland - Swifterbant	HA	A/Razorbill/Scotland/7343/14 (H5Nx)	EU - United Kingdom	-6	98.8%
			NA	A/Mallard/Republic of Georgia/4/2012 (H1N1)	EU - Georgia	433	98.7%
	A/Chicken/Netherlands/14003005/2014 (H2N7)	Overijssel - Witharen	HA	A/Great black-backed gull/Iceland/1395/2011 (H2N5)	EU - Iceland	862	98.3%
				A/Herring gull/Iceland/1320/2011 (H2N5)	EU - Iceland	864	
				A/Herring gull/Iceland/1342/2011 (H2N5)	EU - Iceland	862	
				A/Iceland gull/Iceland/1124/2011 (H2N5)	EU - Iceland	871	
			NA	A/Mallard Duck/Netherlands/ 21/2010 (H10N7)	EU - NL - Noord Holland	1275	98.3%
	A/Chicken/Netherlands/14003323/2014 (H5N2)	Gelderland - Bruchem	HA	A/Duck/Hunan/S4120/2011 (H5N2)	AS - China	852	97.6%
			NA	A/Wild bird/Korea/L60-2/2008 (H5N2)	AS - Republic of Korea	1924	97.7%

	A/Chicken/Netherlands/14004070/2014 (H9N1)	Groningen - Uithuizermeeden	HA	A/Teal/Finland/10529/2010 (H9N2)	EU - Finland	1545	99.3%
			NA	A/Mallard/Sweden/816/2014 (H1N1)	EU - Sweden	-264	99.4%
	A/Duck/Netherlands/14015610/2014 (H6N2)	Utrecht - Lopik	HA	A/Mallard Duck/Netherlands/ 10/2012 (H6N1)	EU - NL - Noord Holland	803	99.0%
			NA	A/Swan/Netherlands/ 14000281/2014 (H5N2)	EU - NL - Zuid Holland	313	99.7%
	A/Duck/Netherlands/14016396/2014 (H6N2)	Gelderland - Hierden	HA	A/Mallard Duck/Netherlands/ 10/2012 (H6N1)	EU - NL - Noord Holland	811	99.0%
			NA	A/Swan/Netherlands/ 14000281/2014 (H5N2)	EU - NL - Zuid Holland	321	99.6%
	A/Duck/Netherlands/14016168/2014 (H6N8)	Gelderland - Putten	HA	A/Mallard Duck/Netherlands/ 15/2011 (H6N8)	EU - NL - Noord Holland	1168	97.8%
				A/Mallard Duck/Netherlands/ 20/2011 (H6N8)	EU - NL - Noord Holland	1158	
			NA	A/Mallard Duck/Netherlands/ 15/2011 (H6N8)	EU - NL - Noord Holland	1168	98.2%
2015	A/Chicken/NL-Barneveld/15004745-001-005/2015 (H7N7)	Gelderland - Barneveld	HA	A/Mallard Duck/Netherlands/ 23/2013 (H7N3)	EU - NL - Zuid Holland	701	98.5%
			NA	A/Mallard Duck/Netherlands/ 13/2013 (H7N7)	EU - NL - Zuid Holland	456	98.8%
	A/Chicken/Netherlands/15005968-056060/2015 (H5N2)	Noord Brabant - Milheeze	HA	A/Razorbill/Scotland/7343/14 (H5Nx)	EU - United Kingdom	395	96.6%
			NA	A/Mallard Duck/Netherlands/ 9/2014 (H6N2)	EU - NL - Zuid Holland	156	99.4%
	A/Chicken/Netherlands/15007212/2015 (H10N7)	Noord Brabant - Heusden	HA	A/Mallard Duck/Netherlands/ 31/2013 (H10N7)	EU - NL - Zuid Holland	708	97.9%
				A/Mallard Duck/Netherlands/ 32/2013 (H10N7)	EU - NL - Zuid Holland	631	
			NA	A/Mallard Duck/Netherlands/ 5/2012 (H10N7)	EU - NL - Zuid Holland	1081	98.1%
2016	A/Chicken/Netherlands/16007311-037041/2016 (H7N9)	Friesland - Hiaure	HA	A/Mallard Duck/Netherlands/ 24/2014 (H7N3)	EU - NL - Noord Holland	617	99.4%
			NA	A/Duck/Bangladesh/26980/2015 (H7N9)	AS - Bangladesh	181	97.3%
				A/Duck/Bangladesh/26992/2015 (H7N9)	AS - Bangladesh	181	
				A/Duck/Bangladesh/27042/2015 (H7N9)	AS - Bangladesh	181	
	A/Chicken/Netherlands/16010778-021-025/2016 (H2N3)	Overijssel - Bathmen	HA	A/Tufted Duck/Georgia/1/2012 (H2N3)	EU - Georgia	1347	97.2%
			NA	A/Mallard Duck/Netherlands/ 24/2014 (H7N3)	EU - NL - Noord Holland	700	98.9%

AS, Asia; EU, Europe; NL, The Netherlands

* two viruses were isolated from a single poultry farm

Supplementary Figure S1. Phylogenetic trees of HA genes.

Neighbour joining (NJ) phylogenetic trees of the hemagglutinin (HA) gene segments of low pathogenic avian influenza (LPAI) viruses from poultry, detected as part of the national avian influenza (AI) surveillance program in the Netherlands, January 2006-September 2016. Nucleotide (nt) sequences of cluster representatives, poultry viruses and top 50 BLAST hits were aligned for each HA separately: H1 (1659 nt), H2 (1679 nt), H3 (1699 nt), H5 (1571 nt), H6 (1596 nt), H7 (1494 nt), (H8 1626 nt), H9 (1567 nt) and H10 (1585 nt). Phylogenetic trees were generated using the Tamura-Nei substitution model with a gamma distribution (shape parameter = 1) for rate variation within the MEGA7 software package. Bootstrap support values above 70 (1,000 replicates) are shown at the branches. Colours represent cluster representatives (blue), poultry viruses (red), and most identical wild bird viruses as determined by BLAST (red). Symbols represent poultry viruses most identical to wild bird viruses isolated in the Netherlands (dots), other European countries (squares) and Asia (triangles). We gratefully acknowledge the authors, originating and submitting laboratories of the sequences from GISAID's EpiFlu database [28] on which this research is based. All submitters of data may be contacted directly via the GISAID website (http://www.gisaid.org).

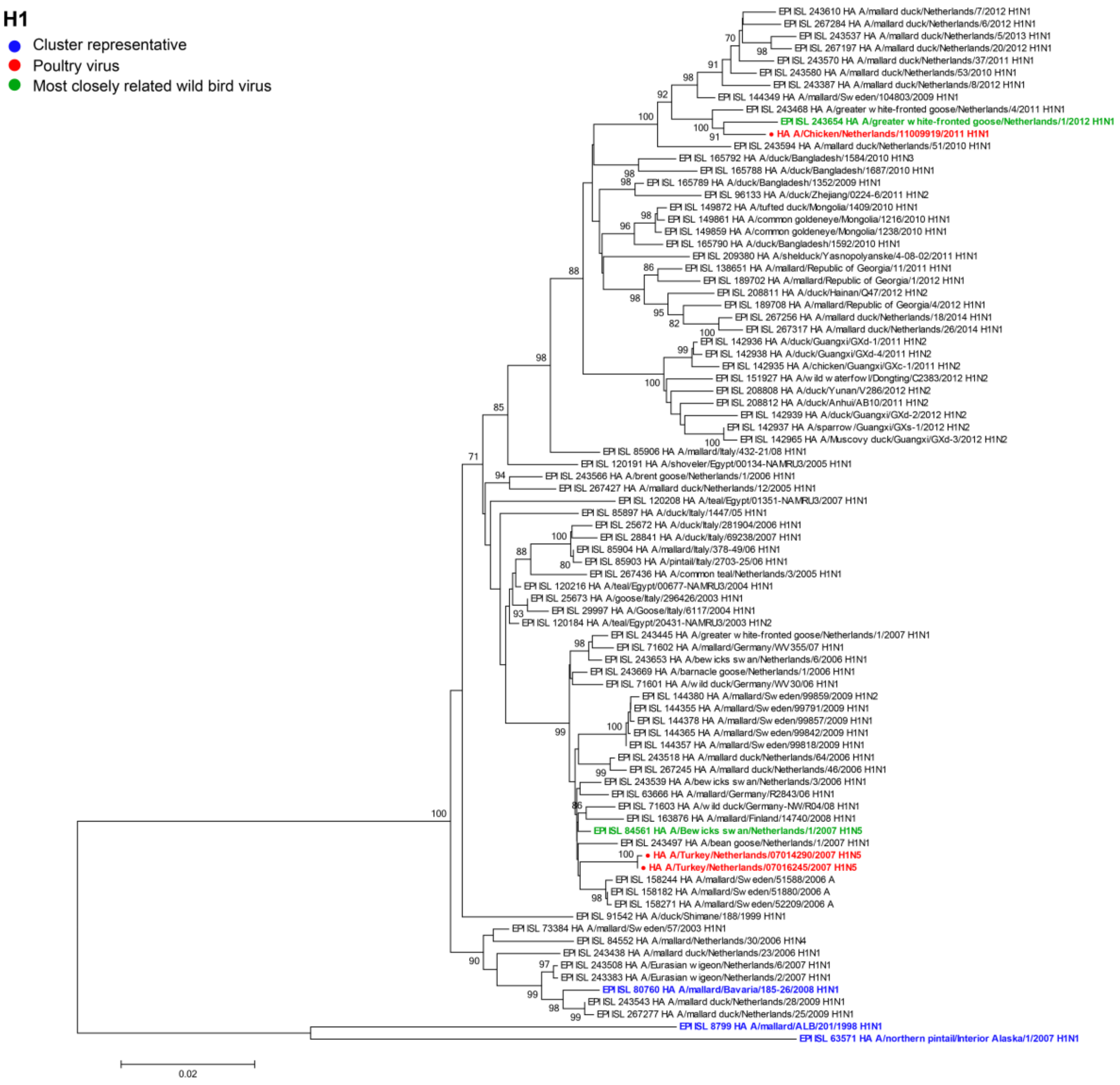
H1
Cluster representative
Poultry virus
Most closely related wild bird virus
EPI ISL 243610 HA A/mallard duck/Netherlands/7/2012 H1N1
EPI ISL 267284 HA A/mallard duck/Netherlands/6/2012 H1N1
EPI ISL 243537 HA A/mallard duck/Netherlands/5/2013 H1N1
EPI ISL 267197 HA A/mallard duck/Netherlands/20/2012 H1N1
EPI ISL 243570 HA A/mallard duck/Netherlands/37/2011 H1N1
EPI ISL 243580 HA A/mallard duck/Netherlands/53/2010 H1N1
EPI ISL 243387 HA A/mallard duck/Netherlands/8/2012 H1N1
EPI ISL 144349 HA A/mallard/Sweden/104803/2009 H1N1
EPI ISL 243468 HA A/greater white-fronted goose/Netherlands/4/2011 H1N1
EPI ISL 243654 HA A/greater white-fronted goose/Netherlands/1/2012 H1N1
HA A/Chicken/Netherlands/11009919/2011 H1N1
EPI ISL 243594 HA A/mallard duck/Netherlands/51/2010 H1N1
EPI ISL 165792 HA A/duck/Bangladesh/1584/2010 H1N3
EPI ISL 165788 HA A/duck/Bangladesh/1687/2010 H1N1
EPI ISL 165789 HA A/duck/Bangladesh/1352/2009 H1N1
EPI ISL 96133 HA A/duck/Zhejiang/0224-6/2011 H1N2
EPI ISL 149872 HA A/tufted duck/Mongolia/1409/2010 H1N1
EPI ISL 149861 HA A/common goldeneye/Mongolia/1216/2010 H1N1
EPI ISL 149859 HA A/common goldeneye/Mongolia/1238/2010 H1N1
EPI ISL 165790 HA A/duck/Bangladesh/1592/2010 H1N1
EPI ISL 209380 HA A/shelduck/Yasnopolyanske/4-08-02/2011 H1N1
EPI ISL 138651 HA A/mallard/Republic of Georgia/11/2011 H1N1
EPI ISL 189702 HA A/mallard/Republic of Georgia/1/2012 H1N1
EPI ISL 208811 HA A/duck/Hainan/Q47/2012 H1N2
EPI ISL 189708 HA A/mallard/Republic of Georgia/4/2012 H1N1
EPI ISL 267256 HA A/mallard duck/Netherlands/18/2014 H1N1
EPI ISL 267317 HA A/mallard duck/Netherlands/26/2014 H1N1
EPI ISL 142936 HA A/duck/Guangxi/GXd-1/2011 H1N2
EPI ISL 142938 HA A/duck/Guangxi/GXd-4/2011 H1N2
EPI ISL 142935 HA A/chicken/Guangxi/GXc-1/2011 H1N2
EPI ISL 151927 HA A/wild waterfowl/Dongting/C2383/2012 H1N2
EPI ISL 208808 HA A/duck/Yunan/V286/2012 H1N2
EPI ISL 208812 HA A/duck/Anhui/AB10/2011 H1N2
EPI ISL 142939 HA A/duck/Guangxi/GXd-2/2012 H1N2
EPI ISL 142937 HA A/sparrow/Guangxi/GXs-1/2012 H1N2
EPI ISL 142965 HA A/Muscovy duck/Guangxi/GXd-3/2012 H1N2
EPI ISL 85906 HA A/mallard/Italy/432-21/08 H1N1
EPI ISL 120191 HA A/shoveler/Egypt/00134-NAMRU3/2005 H1N1
EPI ISL 243566 HA A/brent goose/Netherlands/1/2006 H1N1
EPI ISL 267427 HA A/mallard duck/Netherlands/12/2005 H1N1
EPI ISL 120208 HA A/teal/Egypt/01351-NAMRU3/2007 H1N1
EPI ISL 85897 HA A/duck/Italy/1447/05 H1N1
EPI ISL 25672 HA A/duck/Italy/281904/2006 H1N1
EPI ISL 28841 HA A/duck/Italy/69238/2007 H1N1
EPI ISL 85904 HA A/mallard/Italy/378-49/06 H1N1
EPI ISL 85903 HA A/pintail/Italy/2703-25/06 H1N1
EPI ISL 267436 HA A/common teal/Netherlands/3/2005 H1N1
EPI ISL 120216 HA A/teal/Egypt/00677-NAMRU3/2004 H1N1
EPI ISL 25673 HA A/goose/Italy/296426/2003 H1N1
EPI ISL 29997 HA A/Goose/Italy/6117/2004 H1N1
EPI ISL 120184 HA A/teal/Egypt/20431-NAMRU3/2003 H1N2
EPI ISL 243445 HA A/greater white-fronted goose/Netherlands/1/2007 H1N1
EPI ISL 71602 HA A/mallard/Germany/WV355/07 H1N1
EPI ISL 243653 HA A/bewicks swan/Netherlands/6/2006 H1N1
EPI ISL 243669 HA A/barnacle goose/Netherlands/1/2006 H1N1
EPI ISL 71601 HA A/wild duck/Germany/WV30/06 H1N1
EPI ISL 144380 HA A/mallard/Sweden/99859/2009 H1N2
EPI ISL 144355 HA A/mallard/Sweden/99791/2009 H1N1
EPI ISL 144378 HA A/mallard/Sweden/99857/2009 H1N1
EPI ISL 144365 HA A/mallard/Sweden/99842/2009 H1N1
EPI ISL 144357 HA A/mallard/Sweden/99818/2009 H1N1
EPI ISL 243518 HA A/mallard duck/Netherlands/64/2006 H1N1
EPI ISL 267245 HA A/mallard duck/Netherlands/46/2006 H1N1
EPI ISL 243539 HA A/bewicks swan/Netherlands/3/2006 H1N1
EPI ISL 63666 HA A/mallard/Germany/R2843/06 H1N1
EPI ISL 71603 HA A/wild duck/Germany-NW/R04/08 H1N1
EPI ISL 163876 HA A/mallard/Finland/14740/2008 H1N1
EPI ISL 84561 HA A/Bewicks swan/Netherlands/1/2007 H1N5
EPI ISL 243497 HA A/bean goose/Netherlands/1/2007 H1N1
HA A/Turkey/Netherlands/07014290/2007 H1N5
HA A/Turkey/Netherlands/07016245/2007 H1N5
EPI ISL 158244 HA A/mallard/Sweden/51588/2006 A
EPI ISL 158182 HA A/mallard/Sweden/51880/2006 A
EPI ISL 158271 HA A/mallard/Sweden/52209/2006 A
EPI ISL 91542 HA A/duck/Shimane/188/1999 H1N1
EPI ISL 73384 HA A/mallard/Sweden/57/2003 H1N1
EPI ISL 84552 HA A/mallard/Netherlands/30/2006 H1N4
EPI ISL 243438 HA A/mallard duck/Netherlands/23/2006 H1N1
EPI ISL 243508 HA A/Eurasian wigeon/Netherlands/6/2007 H1N1
EPI ISL 243383 HA A/Eurasian wigeon/Netherlands/2/2007 H1N1
EPI ISL 80760 HA A/mallard/Bavaria/185-26/2008 H1N1
EPI ISL 243543 HA A/mallard duck/Netherlands/28/2009 H1N1
EPI ISL 267277 HA A/mallard duck/Netherlands/25/2009 H1N1
EPI ISL 8799 HA A/mallard/ALB/201/1998 H1N1
EPI ISL 63571 HA A/northern pintail/Interior Alaska/1/2007 H1N1
0.02

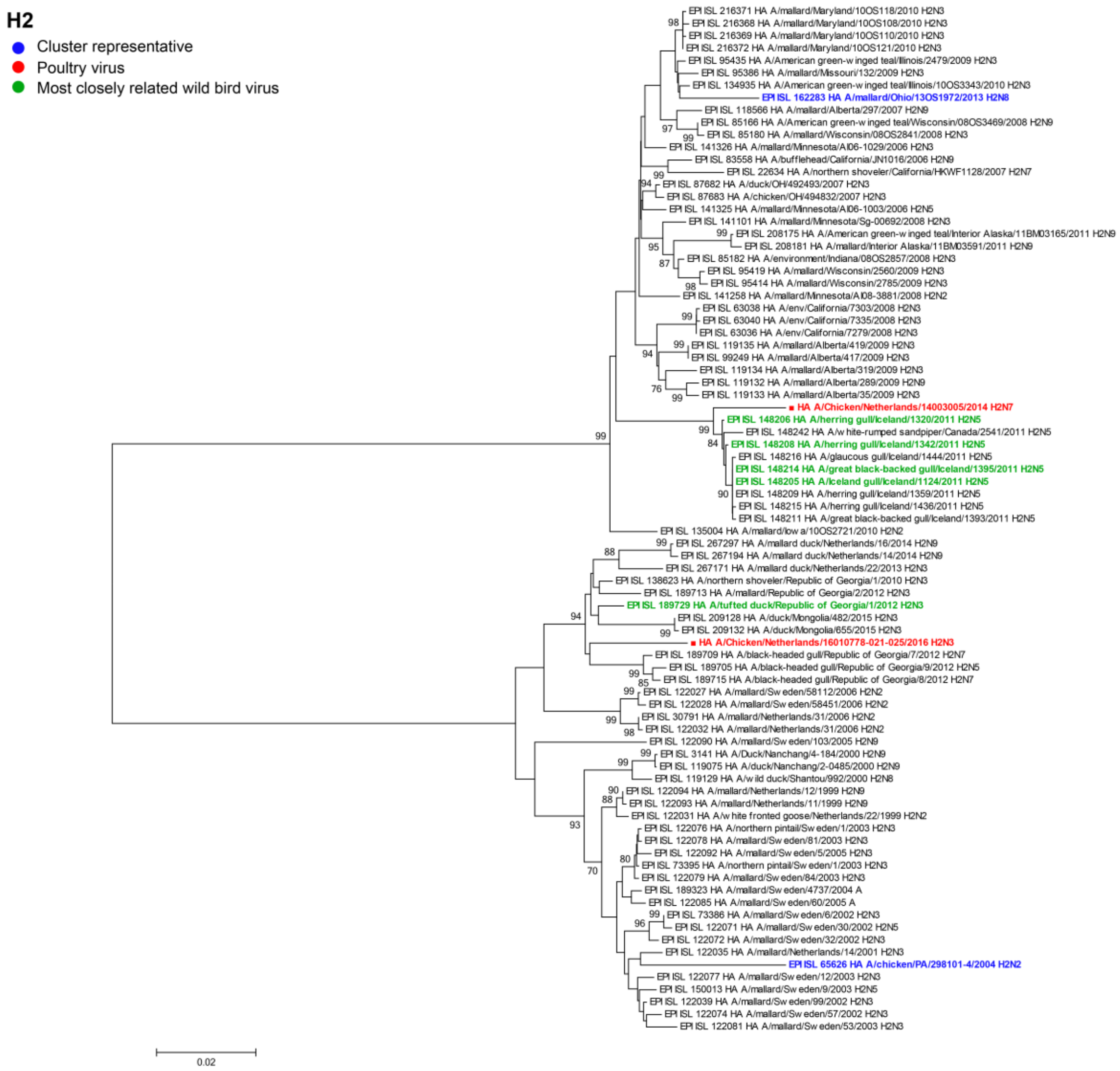
H2
Cluster representative
Poultry virus
Most closely related wild bird virus
EPI ISL 216371 HA A/mallard/Maryland/10OS118/2010 H2N3
EPI ISL 216368 HA A/mallard/Maryland/10OS108/2010 H2N3
EPI ISL 216369 HA A/mallard/Maryland/10OS110/2010 H2N3
EPI ISL 216372 HA A/mallard/Maryland/10OS121/2010 H2N3
EPI ISL 95435 HA A/American green-w inged teal/Illinois/2479/2009 H2N3
EPI ISL 95386 HA A/mallard/Missouri/132/2009 H2N3
EPI ISL 134935 HA A/American green-w inged teal/Illinois/10OS3343/2010 H2N3
EPI ISL 162283 HA A/mallard/Ohio/13OS1972/2013 H2N8
EPI ISL 118566 HA A/mallard/Alberta/297/2007 H2N9
EPI ISL 85166 HA A/American green-w inged teal/Wisconsin/08OS3469/2008 H2N9
EPI ISL 85180 HA A/mallard/Wisconsin/08OS2841/2008 H2N3
EPI ISL 141326 HA A/mallard/Minnesota/AI06-1029/2006 H2N3
EPI ISL 83558 HA A/bufflehead/California/JN1016/2006 H2N9
EPI ISL 22634 HA A/northern shoveler/California/HKWF1128/2007 H2N7
EPI ISL 87682 HA A/duck/OH/492493/2007 H2N3
EPI ISL 87683 HA A/chicken/OH/494832/2007 H2N3
EPI ISL 141325 HA A/mallard/Minnesota/AI06-1003/2006 H2N5
EPI ISL 141101 HA A/mallard/Minnesota/Sg-00692/2008 H2N3
EPI ISL 208175 HA A/American green-w inged teal/Interior Alaska/11BM03165/2011 H2N9
EPI ISL 208181 HA A/mallard/Interior Alaska/11BM03591/2011 H2N9
EPI ISL 85182 HA A/environment/Indiana/08OS2857/2008 H2N3
EPI ISL 95419 HA A/mallard/Wisconsin/2560/2009 H2N3
EPI ISL 95414 HA A/mallard/Wisconsin/2785/2009 H2N3
EPI ISL 141258 HA A/mallard/Minnesota/AI08-3881/2008 H2N2
EPI ISL 63038 HA A/env/California/7303/2008 H2N3
EPI ISL 63040 HA A/env/California/7335/2008 H2N3
EPI ISL 63036 HA A/env/California/7279/2008 H2N3
EPI ISL 119135 HA A/mallard/Alberta/419/2009 H2N3
EPI ISL 99249 HA A/mallard/Alberta/417/2009 H2N3
EPI ISL 119134 HA A/mallard/Alberta/319/2009 H2N3
EPI ISL 119132 HA A/mallard/Alberta/289/2009 H2N9
EPI ISL 119133 HA A/mallard/Alberta/35/2009 H2N3
HA A/Chicken/Netherlands/14003005/2014 H2N7
EPI ISL 148206 HA A/herring gull/Iceland/1320/2011 H2N5
EPI ISL 148242 HA A/w hite-rumped sandpiper/Canada/2541/2011 H2N5
EPI ISL 148208 HA A/herring gull/Iceland/1342/2011 H2N5
EPI ISL 148216 HA A/glaucous gull/Iceland/1444/2011 H2N5
EPI ISL 148214 HA A/great black-backed gull/Iceland/1395/2011 H2N5
EPI ISL 148205 HA A/Iceland gull/Iceland/1124/2011 H2N5
EPI ISL 148209 HA A/herring gull/Iceland/1359/2011 H2N5
EPI ISL 148215 HA A/herring gull/Iceland/1436/2011 H2N5
EPI ISL 148211 HA A/great black-backed gull/Iceland/1393/2011 H2N5
EPI ISL 135004 HA A/mallard/Iow a/10OS2721/2010 H2N2
EPI ISL 267297 HA A/mallard duck/Netherlands/16/2014 H2N9
EPI ISL 267194 HA A/mallard duck/Netherlands/14/2014 H2N9
EPI ISL 267171 HA A/mallard duck/Netherlands/22/2013 H2N3
EPI ISL 138623 HA A/northern shoveler/Republic of Georgia/1/2010 H2N3
EPI ISL 189713 HA A/mallard/Republic of Georgia/2/2012 H2N3
EPI ISL 189729 HA A/tufted duck/Republic of Georgia/1/2012 H2N3
EPI ISL 209128 HA A/duck/Mongolia/482/2015 H2N3
EPI ISL 209132 HA A/duck/Mongolia/655/2015 H2N3
HA A/Chicken/Netherlands/16010778-021-025/2016 H2N3
EPI ISL 189709 HA A/black-headed gull/Republic of Georgia/7/2012 H2N7
EPI ISL 189705 HA A/black-headed gull/Republic of Georgia/9/2012 H2N5
EPI ISL 189715 HA A/black-headed gull/Republic of Georgia/8/2012 H2N7
EPI ISL 122027 HA A/mallard/Sw eden/58112/2006 H2N2
EPI ISL 122028 HA A/mallard/Sw eden/58451/2006 H2N2
EPI ISL 30791 HA A/mallard/Netherlands/31/2006 H2N2
EPI ISL 122032 HA A/mallard/Netherlands/31/2006 H2N2
EPI ISL 122090 HA A/mallard/Sw eden/103/2005 H2N9
EPI ISL 3141 HA A/Duck/Nanchang/4-184/2000 H2N9
EPI ISL 119075 HA A/duck/Nanchang/2-0485/2000 H2N9
EPI ISL 119129 HA A/w ild duck/Shantou/992/2000 H2N8
EPI ISL 122094 HA A/mallard/Netherlands/12/1999 H2N9
EPI ISL 122093 HA A/mallard/Netherlands/11/1999 H2N9
EPI ISL 122031 HA A/w hite fronted goose/Netherlands/22/1999 H2N2
EPI ISL 122076 HA A/northern pintail/Sw eden/1/2003 H2N3
EPI ISL 122078 HA A/mallard/Sw eden/81/2003 H2N3
EPI ISL 122092 HA A/mallard/Sw eden/5/2005 H2N3
EPI ISL 73395 HA A/northern pintail/Sw eden/1/2003 H2N3
EPI ISL 122079 HA A/mallard/Sw eden/84/2003 H2N3
EPI ISL 189323 HA A/mallard/Sw eden/4737/2004 A
EPI ISL 122085 HA A/mallard/Sw eden/60/2005 A
EPI ISL 73386 HA A/mallard/Sw eden/6/2002 H2N3
EPI ISL 122071 HA A/mallard/Sw eden/30/2002 H2N5
EPI ISL 122072 HA A/mallard/Sw eden/32/2002 H2N3
EPI ISL 122035 HA A/mallard/Netherlands/14/2001 H2N3
EPI ISL 65626 HA A/chicken/PA/298101-4/2004 H2N2
EPI ISL 122077 HA A/mallard/Sw eden/12/2003 H2N3
EPI ISL 150013 HA A/mallard/Sw eden/9/2003 H2N5
EPI ISL 122039 HA A/mallard/Sw eden/99/2002 H2N3
EPI ISL 122074 HA A/mallard/Sw eden/57/2002 H2N3
EPI ISL 122081 HA A/mallard/Sw eden/53/2003 H2N3
0.02

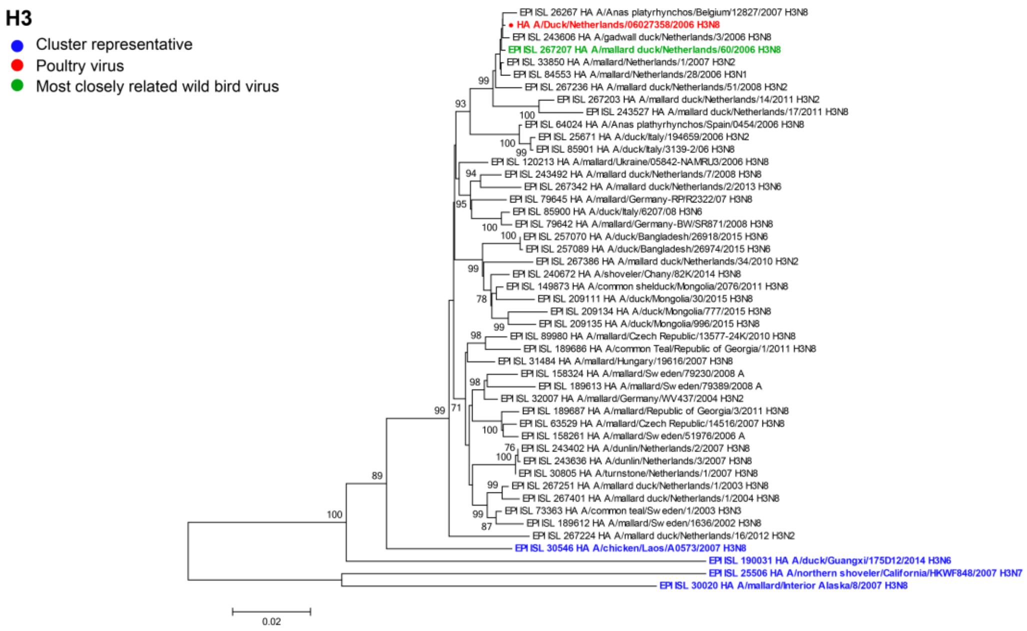
H3
Cluster representative
Poultry virus
Most closely related wild bird virus
EPI ISL 26267 HA A/Anas platyrhynchos/Belgium/12827/2007 H3N8
HA A/Duck/Netherlands/06027358/2006 H3N8
EPI ISL 243606 HA A/gadwall duck/Netherlands/3/2006 H3N8
EPI ISL 267207 HA A/mallard duck/Netherlands/60/2006 H3N8
EPI ISL 33850 HA A/mallard/Netherlands/1/2007 H3N2
EPI ISL 84553 HA A/mallard/Netherlands/28/2006 H3N1
EPI ISL 267236 HA A/mallard duck/Netherlands/51/2008 H3N2
EPI ISL 267203 HA A/mallard duck/Netherlands/14/2011 H3N2
EPI ISL 243527 HA A/mallard duck/Netherlands/17/2011 H3N8
EPI ISL 64024 HA A/Anas plathyrhynchos/Spain/0454/2006 H3N8
EPI ISL 25671 HA A/duck/Italy/194659/2006 H3N2
EPI ISL 85901 HA A/duck/Italy/3139-2/06 H3N8
EPI ISL 120213 HA A/mallard/Ukraine/05842-NAMRU3/2006 H3N8
EPI ISL 243492 HA A/mallard duck/Netherlands/7/2008 H3N8
EPI ISL 267342 HA A/mallard duck/Netherlands/2/2013 H3N6
EPI ISL 79645 HA A/mallard/Germany-RP/R2322/07 H3N8
EPI ISL 85900 HA A/duck/Italy/6207/08 H3N6
EPI ISL 79642 HA A/mallard/Germany-BW/SR871/2008 H3N8
EPI ISL 257070 HA A/duck/Bangladesh/26918/2015 H3N6
EPI ISL 257089 HA A/duck/Bangladesh/26974/2015 H3N6
EPI ISL 267386 HA A/mallard duck/Netherlands/34/2010 H3N2
EPI ISL 240672 HA A/shoveler/Chany/82K/2014 H3N8
EPI ISL 149873 HA A/common shelduck/Mongolia/2076/2011 H3N8
EPI ISL 209111 HA A/duck/Mongolia/30/2015 H3N8
EPI ISL 209134 HA A/duck/Mongolia/777/2015 H3N8
EPI ISL 209135 HA A/duck/Mongolia/996/2015 H3N8
EPI ISL 89980 HA A/mallard/Czech Republic/13577-24K/2010 H3N8
EPI ISL 189686 HA A/common Teal/Republic of Georgia/1/2011 H3N8
EPI ISL 31484 HA A/mallard/Hungary/19616/2007 H3N8
EPI ISL 158324 HA A/mallard/Sw eden/79230/2008 A
EPI ISL 189613 HA A/mallard/Sw eden/79389/2008 A
EPI ISL 32007 HA A/mallard/Germany/WV437/2004 H3N2
EPI ISL 189687 HA A/mallard/Republic of Georgia/3/2011 H3N8
EPI ISL 63529 HA A/mallard/Czech Republic/14516/2007 H3N8
EPI ISL 158261 HA A/mallard/Sw eden/51976/2006 A
EPI ISL 243402 HA A/dunlin/Netherlands/2/2007 H3N8
EPI ISL 243636 HA A/dunlin/Netherlands/3/2007 H3N8
EPI ISL 30805 HA A/turnstone/Netherlands/1/2007 H3N8
EPI ISL 267251 HA A/mallard duck/Netherlands/1/2003 H3N8
EPI ISL 267401 HA A/mallard duck/Netherlands/1/2004 H3N8
EPI ISL 73363 HA A/common teal/Sw eden/1/2003 H3N3
EPI ISL 189612 HA A/mallard/Sw eden/1636/2002 H3N8
EPI ISL 267224 HA A/mallard duck/Netherlands/16/2012 H3N2
EPI ISL 30546 HA A/chicken/Laos/A0573/2007 H3N8
EPI ISL 190031 HA A/duck/Guangxi/175D12/2014 H3N6
EPI ISL 25506 HA A/northern shoveler/California/HKWF848/2007 H3N7
EPI ISL 30020 HA A/mallard/Interior Alaska/8/2007 H3N8
0.02

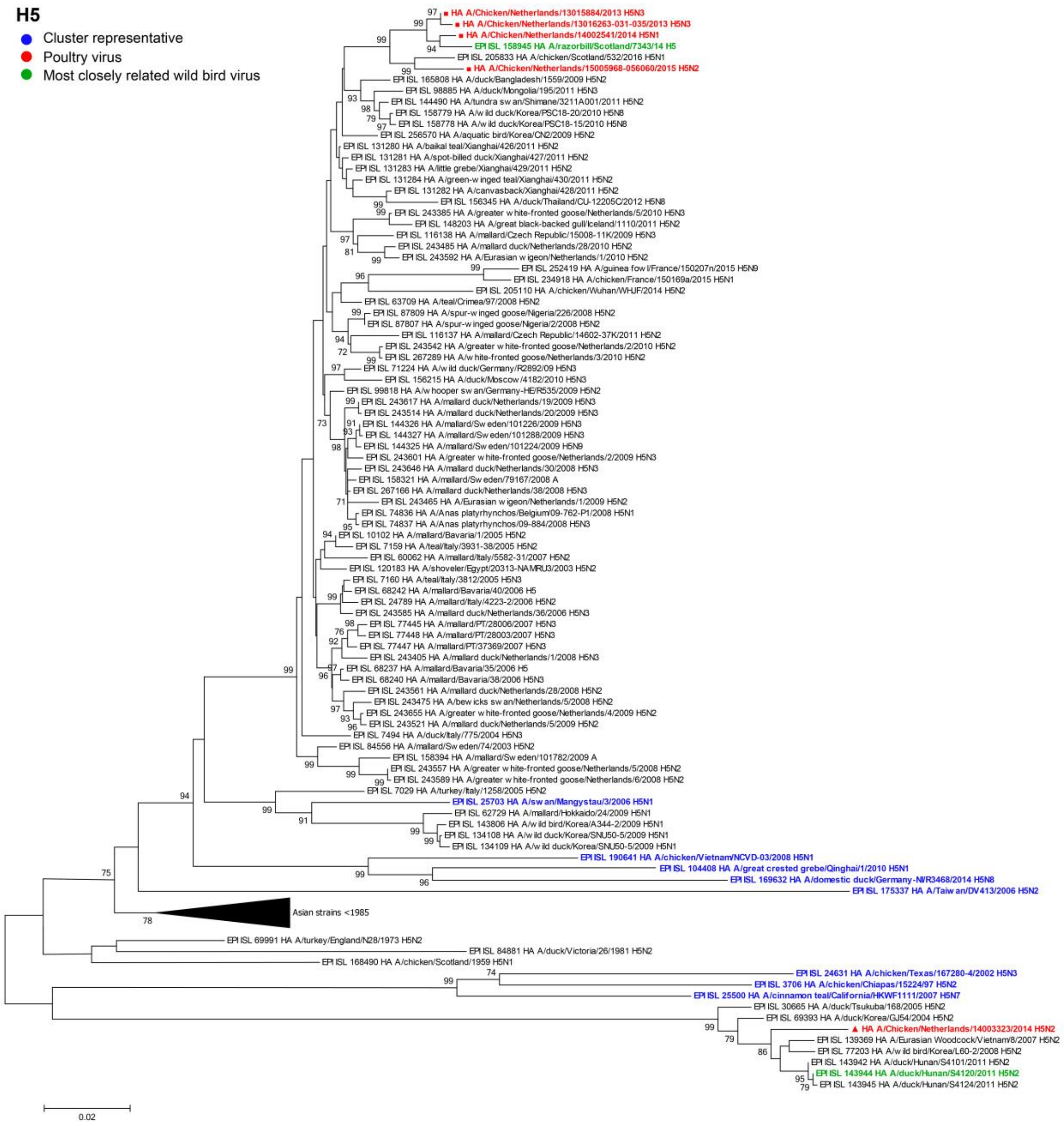
H5
Cluster representative
Poultry virus
Most closely related wild bird virus
HA A/Chicken/Netherlands/13015884/2013 H5N3
HA A/Chicken/Netherlands/13016263-031-035/2013 H5N3
HA A/Chicken/Netherlands/14002541/2014 H5N1
EPI ISL 158945 HA A/razorbill/Scotland/7343/14 H5
EPI ISL 205833 HA A/chicken/Scotland/532/2016 H5N1
HA A/Chicken/Netherlands/15005968-056060/2015 H5N2
EPI ISL 165808 HA A/duck/Bangladesh/1559/2009 H5N2
EPI ISL 98885 HA A/duck/Mongolia/195/2011 H5N3
EPI ISL 144490 HA A/tundra swan/Shimane/3211A001/2011 H5N2
EPI ISL 158779 HA A/wild duck/Korea/PSC18-20/2010 H5N8
EPI ISL 158778 HA A/wild duck/Korea/PSC18-15/2010 H5N8
EPI ISL 256570 HA A/aquatic bird/Korea/CN2/2009 H5N2
EPI ISL 131280 HA A/baikal teal/Xianghai/426/2011 H5N2
EPI ISL 131281 HA A/spot-billed duck/Xianghai/427/2011 H5N2
EPI ISL 131283 HA A/little grebe/Xianghai/429/2011 H5N2
EPI ISL 131284 HA A/green-winged teal/Xianghai/430/2011 H5N2
EPI ISL 131282 HA A/canvasback/Xianghai/428/2011 H5N2
EPI ISL 156345 HA A/duck/Thailand/CU-12205C/2012 H5N8
EPI ISL 243385 HA A/greater white-fronted goose/Netherlands/5/2010 H5N3
EPI ISL 148203 HA A/great black-backed gull/Iceland/1110/2011 H5N2
EPI ISL 116138 HA A/mallard/Czech Republic/15008-11K/2009 H5N3
EPI ISL 243485 HA A/mallard duck/Netherlands/28/2010 H5N2
EPI ISL 243592 HA A/Eurasian wigeon/Netherlands/1/2010 H5N2
EPI ISL 252419 HA A/guinea fowl/France/150207n/2015 H5N9
EPI ISL 234918 HA A/chicken/France/150169a/2015 H5N1
EPI ISL 205110 HA A/chicken/Wuhan/WHJF/2014 H5N2
EPI ISL 63709 HA A/teal/Crimea/97/2008 H5N2
EPI ISL 87809 HA A/spur-winged goose/Nigeria/226/2008 H5N2
EPI ISL 87807 HA A/spur-winged goose/Nigeria/2/2008 H5N2
EPI ISL 116137 HA A/mallard/Czech Republic/14602-37K/2011 H5N2
EPI ISL 243542 HA A/greater white-fronted goose/Netherlands/2/2010 H5N2
EPI ISL 267289 HA A/white-fronted goose/Netherlands/3/2010 H5N2
EPI ISL 71224 HA A/wild duck/Germany/R2892/09 H5N3
EPI ISL 156215 HA A/duck/Moscow/4182/2010 H5N3
EPI ISL 99818 HA A/whooper swan/Germany-HE/R535/2009 H5N2
EPI ISL 243617 HA A/mallard duck/Netherlands/19/2009 H5N3
EPI ISL 243514 HA A/mallard duck/Netherlands/20/2009 H5N3
EPI ISL 144326 HA A/mallard/Sweden/101226/2009 H5N3
EPI ISL 144327 HA A/mallard/Sweden/101288/2009 H5N3
EPI ISL 144325 HA A/mallard/Sweden/101224/2009 H5N9
EPI ISL 243601 HA A/greater white-fronted goose/Netherlands/2/2009 H5N3
EPI ISL 243646 HA A/mallard duck/Netherlands/30/2008 H5N3
EPI ISL 158321 HA A/mallard/Sweden/79167/2008 A
EPI ISL 267166 HA A/mallard duck/Netherlands/38/2008 H5N3
EPI ISL 243465 HA A/Eurasian wigeon/Netherlands/1/2009 H5N2
EPI ISL 74836 HA A/Anas platyrhynchos/Belgium/09-762-P1/2008 H5N1
EPI ISL 74837 HA A/Anas platyrhynchos/09-884/2008 H5N3
EPI ISL 10102 HA A/mallard/Bavaria/1/2005 H5N2
EPI ISL 7159 HA A/teal/Italy/3931-38/2005 H5N2
EPI ISL 60062 HA A/mallard/Italy/5582-31/2007 H5N2
EPI ISL 120183 HA A/shoveler/Egypt/20313-NAMRU3/2003 H5N2
EPI ISL 7160 HA A/teal/Italy/3812/2005 H5N3
EPI ISL 68242 HA A/mallard/Bavaria/40/2006 H5
EPI ISL 24789 HA A/mallard/Italy/4223-2/2006 H5N2
EPI ISL 243585 HA A/mallard duck/Netherlands/36/2006 H5N3
EPI ISL 77445 HA A/mallard/PT/28006/2007 H5N3
EPI ISL 77448 HA A/mallard/PT/28003/2007 H5N3
EPI ISL 77447 HA A/mallard/PT/37369/2007 H5N3
EPI ISL 243405 HA A/mallard duck/Netherlands/1/2008 H5N3
EPI ISL 68237 HA A/mallard/Bavaria/35/2006 H5
EPI ISL 68240 HA A/mallard/Bavaria/38/2006 H5N3
EPI ISL 243561 HA A/mallard duck/Netherlands/28/2008 H5N2
EPI ISL 243475 HA A/bewicks swan/Netherlands/5/2008 H5N2
EPI ISL 243655 HA A/greater white-fronted goose/Netherlands/4/2009 H5N2
EPI ISL 243521 HA A/mallard duck/Netherlands/5/2009 H5N2
EPI ISL 7494 HA A/duck/Italy/775/2004 H5N3
EPI ISL 84556 HA A/mallard/Sweden/74/2003 H5N2
EPI ISL 158394 HA A/mallard/Sweden/101782/2009 A
EPI ISL 243557 HA A/greater white-fronted goose/Netherlands/5/2008 H5N2
EPI ISL 243589 HA A/greater white-fronted goose/Netherlands/6/2008 H5N2
EPI ISL 7029 HA A/turkey/Italy/1258/2005 H5N2
EPI ISL 25703 HA A/swan/Mangystau/3/2006 H5N1
EPI ISL 62729 HA A/mallard/Hokkaido/24/2009 H5N1
EPI ISL 143806 HA A/wild bird/Korea/A344-2/2009 H5N1
EPI ISL 134108 HA A/wild duck/Korea/SNU50-5/2009 H5N1
EPI ISL 134109 HA A/wild duck/Korea/SNU50-5/2009 H5N1
EPI ISL 190641 HA A/chicken/Vietnam/NCVD-03/2008 H5N1
EPI ISL 104408 HA A/great crested grebe/Qinghai/1/2010 H5N1
EPI ISL 169632 HA A/domestic duck/Germany-NIIR3468/2014 H5N8
EPI ISL 175337 HA A/Taiwan/DV413/2006 H5N2
Asian strains <1985
EPI ISL 69991 HA A/turkey/England/N28/1973 H5N2
EPI ISL 84881 HA A/duck/Victoria/26/1981 H5N2
EPI ISL 168490 HA A/chicken/Scotland/1959 H5N1
EPI ISL 24631 HA A/chicken/Texas/167280-4/2002 H5N3
EPI ISL 3706 HA A/chicken/Chiapas/15224/97 H5N2
EPI ISL 25500 HA A/cinnamon teal/California/HKWF1111/2007 H5N7
EPI ISL 30665 HA A/duck/Tsukuba/168/2005 H5N2
EPI ISL 69393 HA A/duck/Korea/GJ54/2004 H5N2
HA A/Chicken/Netherlands/14003323/2014 H5N2
EPI ISL 139369 HA A/Eurasian Woodcock/Vietnam/8/2007 H5N2
EPI ISL 77203 HA A/wild bird/Korea/L60-2/2008 H5N2
EPI ISL 143942 HA A/duck/Hunan/S4101/2011 H5N2
EPI ISL 143944 HA A/duck/Hunan/S4120/2011 H5N2
EPI ISL 143945 HA A/duck/Hunan/S4124/2011 H5N2
0.02

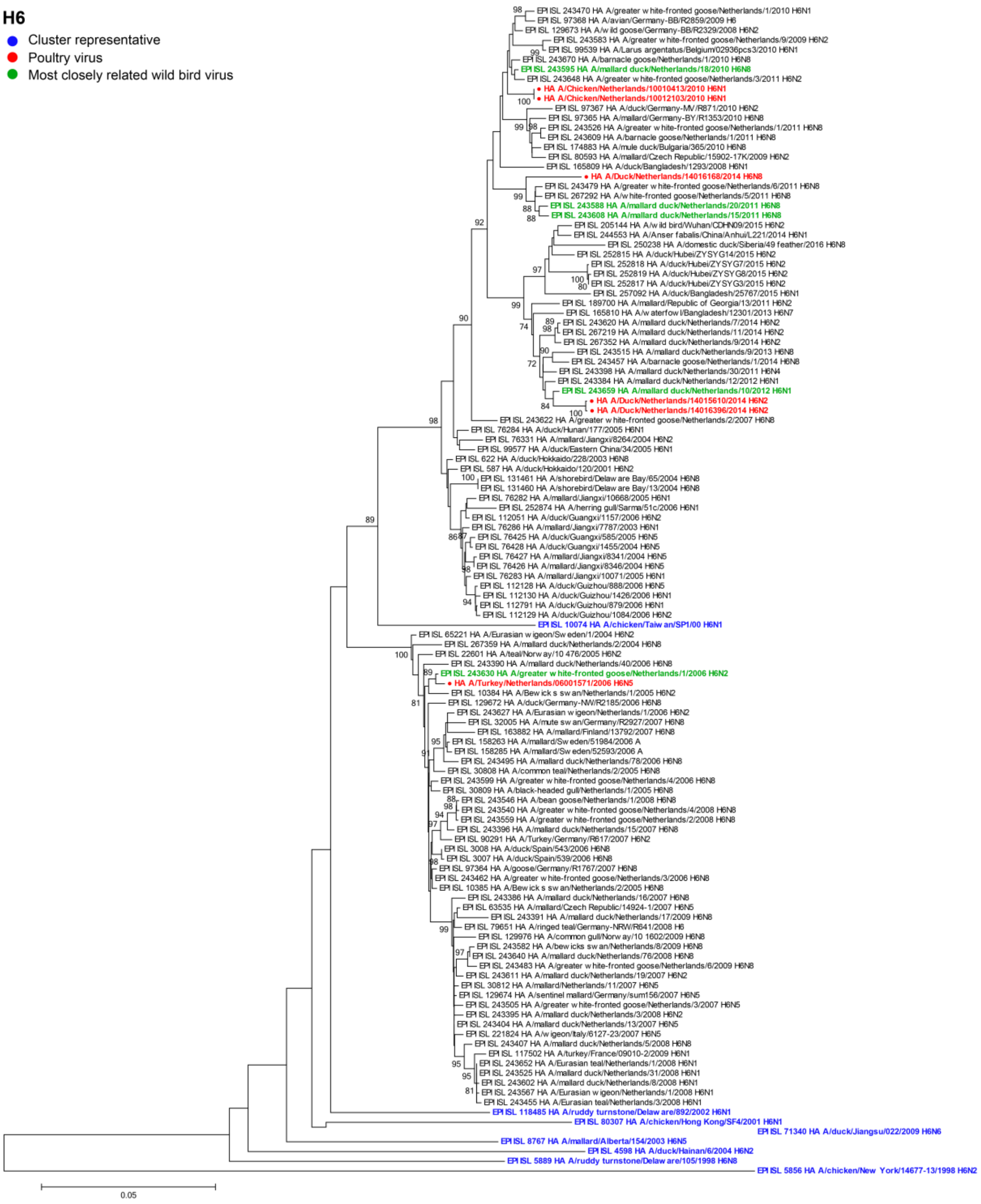
H6
Cluster representative
Poultry virus
Most closely related wild bird virus
EPI ISL 243470 HA A/greater w hite-fronted goose/Netherlands/1/2010 H6N1
EPI ISL 97368 HA A/avian/Germany-BB/R2859/2009 H6
EPI ISL 129673 HA A/w ild goose/Germany-BB/R2329/2008 H6N2
EPI ISL 243583 HA A/greater w hite-fronted goose/Netherlands/9/2009 H6N2
EPI ISL 99539 HA A/Larus argentatus/Belgium/02936pcs3/2010 H6N1
EPI ISL 243670 HA A/barnacle goose/Netherlands/1/2010 H6N8
EPI ISL 243595 HA A/mallard duck/Netherlands/18/2010 H6N8
EPI ISL 243648 HA A/greater w hite-fronted goose/Netherlands/3/2011 H6N2
HA A/Chicken/Netherlands/10010413/2010 H6N1
HA A/Chicken/Netherlands/10012103/2010 H6N1
EPI ISL 97367 HA A/duck/Germany-MV/R871/2010 H6N2
EPI ISL 97365 HA A/mallard/Germany-BY/R1353/2010 H6N8
EPI ISL 243526 HA A/greater w hite-fronted goose/Netherlands/1/2011 H6N8
EPI ISL 243609 HA A/barnacle goose/Netherlands/1/2011 H6N8
EPI ISL 174883 HA A/mule duck/Bulgaria/365/2010 H6N8
EPI ISL 80593 HA A/mallard/Czech Republic/15902-17K/2009 H6N2
EPI ISL 165809 HA A/duck/Bangladesh/1293/2008 H6N1
HA A/Duck/Netherlands/14016168/2014 H6N8
EPI ISL 243479 HA A/greater w hite-fronted goose/Netherlands/6/2011 H6N8
EPI ISL 267292 HA A/w hite-fronted goose/Netherlands/5/2011 H6N8
EPI ISL 243588 HA A/mallard duck/Netherlands/20/2011 H6N8
EPI ISL 243608 HA A/mallard duck/Netherlands/15/2011 H6N8
EPI ISL 205144 HA A/w ild bird/Wuhan/CDHN09/2015 H6N2
EPI ISL 244553 HA A/Anser fabalis/China/Anhui/L221/2014 H6N1
EPI ISL 250238 HA A/domestic duck/Siberia/49 feather/2016 H6N8
EPI ISL 252815 HA A/duck/Hubei/ZYSYG14/2015 H6N2
EPI ISL 252818 HA A/duck/Hubei/ZYSYG7/2015 H6N2
EPI ISL 252819 HA A/duck/Hubei/ZYSYG8/2015 H6N2
EPI ISL 252817 HA A/duck/Hubei/ZYSYG3/2015 H6N2
EPI ISL 257092 HA A/duck/Bangladesh/25767/2015 H6N1
EPI ISL 189700 HA A/mallard/Republic of Georgia/13/2011 H6N2
EPI ISL 165810 HA A/w aterfow l/Bangladesh/12301/2013 H6N7
EPI ISL 243620 HA A/mallard duck/Netherlands/7/2014 H6N2
EPI ISL 267219 HA A/mallard duck/Netherlands/11/2014 H6N2
EPI ISL 267352 HA A/mallard duck/Netherlands/9/2014 H6N2
EPI ISL 243515 HA A/mallard duck/Netherlands/9/2013 H6N8
EPI ISL 243457 HA A/barnacle goose/Netherlands/1/2014 H6N8
EPI ISL 243398 HA A/mallard duck/Netherlands/30/2011 H6N4
EPI ISL 243384 HA A/mallard duck/Netherlands/12/2012 H6N1
EPI ISL 243659 HA A/mallard duck/Netherlands/10/2012 H6N1
HA A/Duck/Netherlands/14015610/2014 H6N2
HA A/Duck/Netherlands/14016396/2014 H6N2
EPI ISL 243622 HA A/greater w hite-fronted goose/Netherlands/2/2007 H6N8
EPI ISL 76284 HA A/duck/Hunan/177/2005 H6N1
EPI ISL 76331 HA A/mallard/Jiangxi/8264/2004 H6N2
EPI ISL 99577 HA A/duck/Eastern China/34/2005 H6N1
EPI ISL 622 HA A/duck/Hokkaido/228/2003 H6N8
EPI ISL 587 HA A/duck/Hokkaido/120/2001 H6N2
EPI ISL 131461 HA A/shorebird/Delaw are Bay/65/2004 H6N8
EPI ISL 131460 HA A/shorebird/Delaw are Bay/13/2004 H6N8
EPI ISL 76282 HA A/mallard/Jiangxi/10668/2005 H6N1
EPI ISL 252874 HA A/herring gull/Sarma/51c/2006 H6N1
EPI ISL 112051 HA A/duck/Guangxi/1157/2006 H6N2
EPI ISL 76286 HA A/mallard/Jiangxi/7787/2003 H6N1
EPI ISL 76425 HA A/duck/Guangxi/585/2005 H6N5
EPI ISL 76428 HA A/duck/Guangxi/1455/2004 H6N5
EPI ISL 76427 HA A/mallard/Jiangxi/8341/2004 H6N5
EPI ISL 76426 HA A/mallard/Jiangxi/8346/2004 H6N5
EPI ISL 76283 HA A/mallard/Jiangxi/10071/2005 H6N1
EPI ISL 112128 HA A/duck/Guizhou/888/2006 H6N5
EPI ISL 112130 HA A/duck/Guizhou/1426/2006 H6N1
EPI ISL 112791 HA A/duck/Guizhou/879/2006 H6N1
EPI ISL 112129 HA A/duck/Guizhou/1084/2006 H6N2
EPI ISL 10074 HA A/chicken/Taiw an/SP1/00 H6N1
EPI ISL 65221 HA A/Eurasian w igeon/Sw eden/1/2004 H6N2
EPI ISL 267359 HA A/mallard duck/Netherlands/2/2004 H6N8
EPI ISL 22601 HA A/teal/Norw ay/10 476/2005 H6N2
EPI ISL 243390 HA A/mallard duck/Netherlands/40/2006 H6N8
EPI ISL 243630 HA A/greater w hite-fronted goose/Netherlands/1/2006 H6N2
HA A/Turkey/Netherlands/06001571/2006 H6N5
EPI ISL 10384 HA A/Bew ick s sw an/Netherlands/1/2005 H6N2
EPI ISL 129672 HA A/duck/Germany-NW/R2185/2006 H6N8
EPI ISL 243627 HA A/Eurasian w igeon/Netherlands/1/2006 H6N2
EPI ISL 32005 HA A/mute sw an/Germany/R2927/2007 H6N8
EPI ISL 163882 HA A/mallard/Finland/13792/2007 H6N8
EPI ISL 158263 HA A/mallard/Sw eden/51984/2006 A
EPI ISL 158285 HA A/mallard/Sw eden/52593/2006 A
EPI ISL 243495 HA A/mallard duck/Netherlands/78/2006 H6N8
EPI ISL 30808 HA A/common teal/Netherlands/2/2005 H6N8
EPI ISL 243599 HA A/greater w hite-fronted goose/Netherlands/4/2006 H6N8
EPI ISL 30809 HA A/black-headed gull/Netherlands/1/2005 H6N8
EPI ISL 243546 HA A/bean goose/Netherlands/1/2008 H6N8
EPI ISL 243540 HA A/greater w hite-fronted goose/Netherlands/4/2008 H6N8
EPI ISL 243559 HA A/greater w hite-fronted goose/Netherlands/2/2008 H6N8
EPI ISL 243396 HA A/mallard duck/Netherlands/15/2007 H6N8
EPI ISL 90291 HA A/Turkey/Germany/R617/2007 H6N2
EPI ISL 3008 HA A/duck/Spain/543/2006 H6N8
EPI ISL 3007 HA A/duck/Spain/539/2006 H6N8
EPI ISL 97364 HA A/goose/Germany/R1767/2007 H6N8
EPI ISL 243462 HA A/greater w hite-fronted goose/Netherlands/3/2006 H6N8
EPI ISL 10385 HA A/Bew ick s sw an/Netherlands/2/2005 H6N8
EPI ISL 243386 HA A/mallard duck/Netherlands/16/2007 H6N8
EPI ISL 63535 HA A/mallard/Czech Republic/14924-1/2007 H6N5
EPI ISL 243391 HA A/mallard duck/Netherlands/17/2009 H6N8
EPI ISL 79651 HA A/ringed teal/Germany-NRW/R641/2008 H6
EPI ISL 129976 HA A/common gull/Norw ay/10 1602/2009 H6N8
EPI ISL 243582 HA A/bew icks sw an/Netherlands/8/2009 H6N8
EPI ISL 243640 HA A/mallard duck/Netherlands/76/2008 H6N8
EPI ISL 243483 HA A/greater w hite-fronted goose/Netherlands/6/2009 H6N8
EPI ISL 243611 HA A/mallard duck/Netherlands/19/2007 H6N2
EPI ISL 30812 HA A/mallard/Netherlands/11/2007 H6N5
EPI ISL 129674 HA A/sentinel mallard/Germany/sum156/2007 H6N5
EPI ISL 243505 HA A/greater w hite-fronted goose/Netherlands/3/2007 H6N5
EPI ISL 243395 HA A/mallard duck/Netherlands/3/2008 H6N2
EPI ISL 243404 HA A/mallard duck/Netherlands/13/2007 H6N5
EPI ISL 221824 HA A/w igeon/Italy/6127-23/2007 H6N5
EPI ISL 243407 HA A/mallard duck/Netherlands/5/2008 H6N8
EPI ISL 117502 HA A/turkey/France/09010-2/2009 H6N1
EPI ISL 243652 HA A/Eurasian teal/Netherlands/1/2008 H6N1
EPI ISL 243525 HA A/mallard duck/Netherlands/31/2008 H6N1
EPI ISL 243602 HA A/mallard duck/Netherlands/8/2008 H6N1
EPI ISL 243567 HA A/Eurasian w igeon/Netherlands/1/2008 H6N1
EPI ISL 243455 HA A/Eurasian teal/Netherlands/3/2008 H6N1
EPI ISL 118485 HA A/ruddy turnstone/Delaw are/892/2002 H6N1
EPI ISL 80307 HA A/chicken/Hong Kong/SF4/2001 H6N1
EPI ISL 71340 HA A/duck/Jiangsu/022/2009 H6N6
EPI ISL 8767 HA A/mallard/Alberta/154/2003 H6N5
EPI ISL 4598 HA A/duck/Hainan/6/2004 H6N2
EPI ISL 5889 HA A/ruddy turnstone/Delaw are/105/1998 H6N8
EPI ISL 5856 HA A/chicken/New York/14677-13/1998 H6N2
0.05

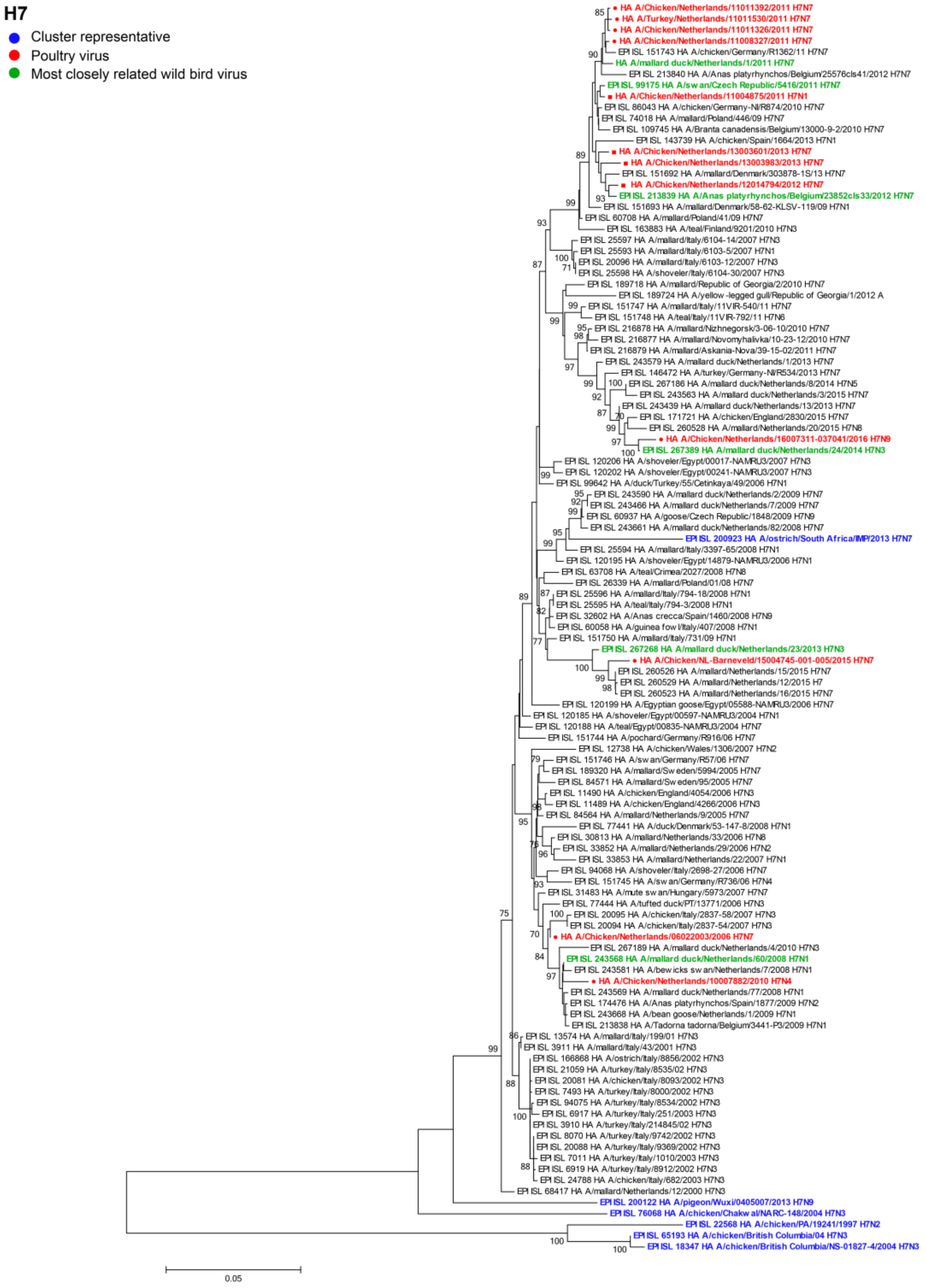
H7
Cluster representative
Poultry virus
Most closely related wild bird virus
HA A/Chicken/Netherlands/11011392/2011 H7N7
HA A/Turkey/Netherlands/11011530/2011 H7N7
HA A/Chicken/Netherlands/11011326/2011 H7N7
HA A/Chicken/Netherlands/11008327/2011 H7N7
EPI ISL 151743 HA A/chicken/Germany/R1362/11 H7N7
HA A/mallard duck/Netherlands/1/2011 H7N7
EPI ISL 213840 HA A/Anas platyrhynchos/Belgium/25576cls41/2012 H7N7
EPI ISL 99175 HA A/swan/Czech Republic/5416/2011 H7N7
HA A/Chicken/Netherlands/11004875/2011 H7N1
EPI ISL 86043 HA A/chicken/Germany-NI/R874/2010 H7N7
EPI ISL 74018 HA A/mallard/Poland/446/09 H7N7
EPI ISL 109745 HA A/Branta canadensis/Belgium/13000-9-2/2010 H7N7
EPI ISL 143739 HA A/chicken/Spain/1664/2013 H7N1
HA A/Chicken/Netherlands/13003601/2013 H7N7
HA A/Chicken/Netherlands/13003983/2013 H7N7
EPI ISL 151692 HA A/mallard/Denmark/303878-1S/13 H7N7
HA A/Chicken/Netherlands/12014794/2012 H7N7
EPI ISL 213839 HA A/Anas platyrhynchos/Belgium/23852cls33/2012 H7N7
EPI ISL 151693 HA A/mallard/Denmark/58-62-KLSV-119/09 H7N1
EPI ISL 60708 HA A/mallard/Poland/41/09 H7N7
EPI ISL 163883 HA A/teal/Finland/9201/2010 H7N3
EPI ISL 25597 HA A/mallard/Italy/6104-14/2007 H7N3
EPI ISL 25593 HA A/mallard/Italy/6103-5/2007 H7N1
EPI ISL 20096 HA A/mallard/Italy/6103-12/2007 H7N3
EPI ISL 25598 HA A/shoveler/Italy/6104-30/2007 H7N3
EPI ISL 189718 HA A/mallard/Republic of Georgia/2/2010 H7N7
EPI ISL 189724 HA A/yellow-legged gull/Republic of Georgia/1/2012 A
EPI ISL 151747 HA A/mallard/Italy/11VIR-540/11 H7N7
EPI ISL 151748 HA A/teal/Italy/11VIR-792/11 H7N6
EPI ISL 216878 HA A/mallard/Nizhnegorsk/3-06-10/2010 H7N7
EPI ISL 216877 HA A/mallard/Novomyhalivka/10-23-12/2010 H7N7
EPI ISL 216879 HA A/mallard/Askania-Nova/39-15-02/2011 H7N7
EPI ISL 243579 HA A/mallard duck/Netherlands/1/2013 H7N7
EPI ISL 146472 HA A/turkey/Germany-NI/R534/2013 H7N7
EPI ISL 267186 HA A/mallard duck/Netherlands/8/2014 H7N5
EPI ISL 243563 HA A/mallard duck/Netherlands/3/2015 H7N7
EPI ISL 243439 HA A/mallard duck/Netherlands/13/2013 H7N7
EPI ISL 171721 HA A/chicken/England/2830/2015 H7N7
EPI ISL 260528 HA A/mallard/Netherlands/20/2015 H7N8
HA A/Chicken/Netherlands/16007311-037041/2016 H7N9
EPI ISL 267389 HA A/mallard duck/Netherlands/24/2014 H7N3
EPI ISL 120206 HA A/shoveler/Egypt/00017-NAMRU3/2007 H7N3
EPI ISL 120202 HA A/shoveler/Egypt/00241-NAMRU3/2007 H7N3
EPI ISL 99642 HA A/duck/Turkey/55/Cetinkaya/49/2006 H7N1
EPI ISL 243590 HA A/mallard duck/Netherlands/2/2009 H7N7
EPI ISL 243466 HA A/mallard duck/Netherlands/7/2009 H7N7
EPI ISL 60937 HA A/goose/Czech Republic/1848/2009 H7N9
EPI ISL 243661 HA A/mallard duck/Netherlands/82/2008 H7N7
EPI ISL 200923 HA A/ostrich/South Africa/IMP/2013 H7N7
EPI ISL 25594 HA A/mallard/Italy/3397-65/2008 H7N1
EPI ISL 120195 HA A/shoveler/Egypt/14879-NAMRU3/2006 H7N1
EPI ISL 63708 HA A/teal/Crimea/2027/2008 H7N8
EPI ISL 26339 HA A/mallard/Poland/01/08 H7N7
EPI ISL 25596 HA A/mallard/Italy/794-18/2008 H7N1
EPI ISL 25595 HA A/teal/Italy/794-3/2008 H7N1
EPI ISL 32602 HA A/Anas crecca/Spain/1460/2008 H7N9
EPI ISL 60058 HA A/guinea fowl/Italy/407/2008 H7N1
EPI ISL 151750 HA A/mallard/Italy/731/09 H7N1
EPI ISL 267268 HA A/mallard duck/Netherlands/23/2013 H7N3
HA A/Chicken/NL-Barneveld/15004745-001-005/2015 H7N7
EPI ISL 260526 HA A/mallard/Netherlands/15/2015 H7N7
EPI ISL 260529 HA A/mallard/Netherlands/12/2015 H7
EPI ISL 260523 HA A/mallard/Netherlands/16/2015 H7N7
EPI ISL 120199 HA A/Egyptian goose/Egypt/05588-NAMRU3/2006 H7N7
EPI ISL 120185 HA A/shoveler/Egypt/00597-NAMRU3/2004 H7N1
EPI ISL 120188 HA A/teal/Egypt/00835-NAMRU3/2004 H7N7
EPI ISL 151744 HA A/pochard/Germany/R916/06 H7N7
EPI ISL 12738 HA A/chicken/Wales/1306/2007 H7N2
EPI ISL 151746 HA A/swan/Germany/R57/06 H7N7
EPI ISL 189320 HA A/mallard/Sweden/5994/2005 H7N7
EPI ISL 84571 HA A/mallard/Sweden/95/2005 H7N7
EPI ISL 11490 HA A/chicken/England/4054/2006 H7N3
EPI ISL 11489 HA A/chicken/England/4266/2006 H7N3
EPI ISL 84564 HA A/mallard/Netherlands/9/2005 H7N7
EPI ISL 77441 HA A/duck/Denmark/53-147-8/2008 H7N1
EPI ISL 30813 HA A/mallard/Netherlands/33/2006 H7N8
EPI ISL 33852 HA A/mallard/Netherlands/29/2006 H7N2
EPI ISL 33853 HA A/mallard/Netherlands/22/2007 H7N1
EPI ISL 94068 HA A/shoveler/Italy/2698-27/2006 H7N7
EPI ISL 151745 HA A/swan/Germany/R736/06 H7N4
EPI ISL 31483 HA A/mute swan/Hungary/5973/2007 H7N7
EPI ISL 77444 HA A/tufted duck/PT/13771/2006 H7N3
EPI ISL 20095 HA A/chicken/Italy/2837-58/2007 H7N3
EPI ISL 20094 HA A/chicken/Italy/2837-54/2007 H7N3
HA A/Chicken/Netherlands/06022003/2006 H7N7
EPI ISL 267189 HA A/mallard duck/Netherlands/4/2010 H7N3
EPI ISL 243568 HA A/mallard duck/Netherlands/60/2008 H7N1
EPI ISL 243581 HA A/bewicks swan/Netherlands/7/2008 H7N1
HA A/Chicken/Netherlands/10007882/2010 H7N4
EPI ISL 243569 HA A/mallard duck/Netherlands/77/2008 H7N1
EPI ISL 174476 HA A/Anas platyrhynchos/Spain/1877/2009 H7N2
EPI ISL 243668 HA A/bean goose/Netherlands/1/2009 H7N1
EPI ISL 213838 HA A/Tadorna tadorna/Belgium/3441-P3/2009 H7N1
EPI ISL 13574 HA A/mallard/Italy/199/01 H7N3
EPI ISL 3911 HA A/mallard/Italy/43/2001 H7N3
EPI ISL 166868 HA A/ostrich/Italy/8856/2002 H7N3
EPI ISL 21059 HA A/turkey/Italy/8535/02 H7N3
EPI ISL 20081 HA A/chicken/Italy/8093/2002 H7N3
EPI ISL 7493 HA A/turkey/Italy/8000/2002 H7N3
EPI ISL 94075 HA A/turkey/Italy/8534/2002 H7N3
EPI ISL 6917 HA A/turkey/Italy/251/2003 H7N3
EPI ISL 3910 HA A/turkey/Italy/214845/02 H7N3
EPI ISL 8070 HA A/turkey/Italy/9742/2002 H7N3
EPI ISL 20088 HA A/turkey/Italy/9369/2002 H7N3
EPI ISL 7011 HA A/turkey/Italy/1010/2003 H7N3
EPI ISL 6919 HA A/turkey/Italy/8912/2002 H7N3
EPI ISL 24788 HA A/chicken/Italy/682/2003 H7N3
EPI ISL 68417 HA A/mallard/Netherlands/12/2000 H7N3
EPI ISL 200122 HA A/pigeon/Wuxi/0405007/2013 H7N9
EPI ISL 76068 HA A/chicken/Chakwal/NARC-148/2004 H7N3
EPI ISL 22568 HA A/chicken/PA/19241/1997 H7N2
EPI ISL 65193 HA A/chicken/British Columbia/04 H7N3
EPI ISL 18347 HA A/chicken/British Columbia/NS-01827-4/2004 H7N3
0.05

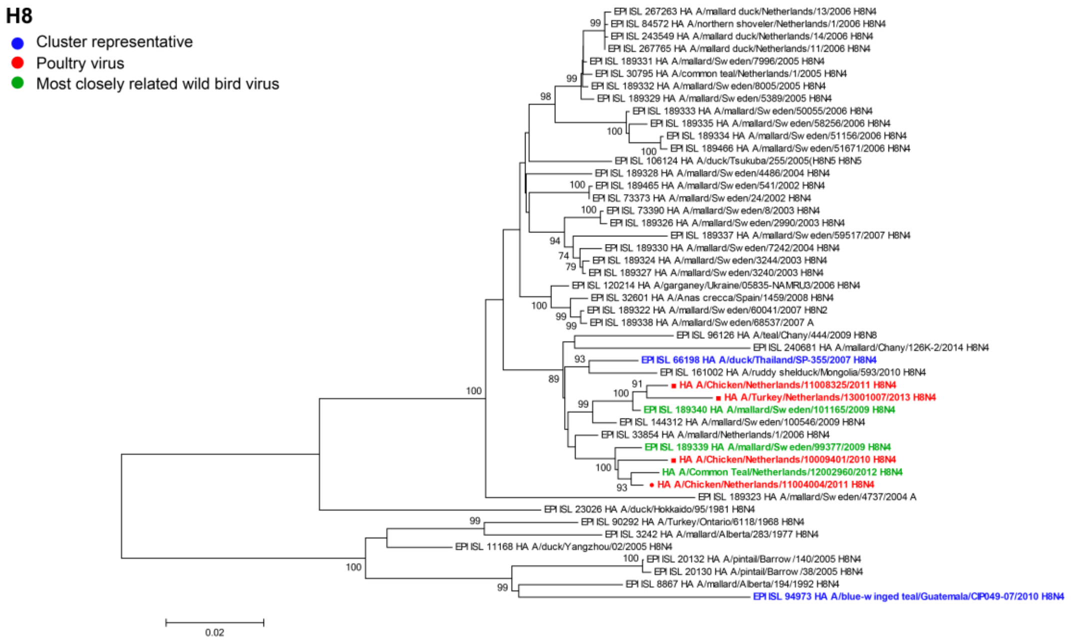
H8
Cluster representative
Poultry virus
Most closely related wild bird virus
EPI ISL 267263 HA A/mallard duck/Netherlands/13/2006 H8N4
EPI ISL 84572 HA A/northern shoveler/Netherlands/1/2006 H8N4
EPI ISL 243549 HA A/mallard duck/Netherlands/14/2006 H8N4
EPI ISL 267765 HA A/mallard duck/Netherlands/11/2006 H8N4
EPI ISL 189331 HA A/mallard/Sw eden/7996/2005 H8N4
EPI ISL 30795 HA A/common teal/Netherlands/1/2005 H8N4
EPI ISL 189332 HA A/mallard/Sw eden/8005/2005 H8N4
EPI ISL 189329 HA A/mallard/Sw eden/5389/2005 H8N4
EPI ISL 189333 HA A/mallard/Sw eden/50055/2006 H8N4
EPI ISL 189335 HA A/mallard/Sw eden/58256/2006 H8N4
EPI ISL 189334 HA A/mallard/Sw eden/51156/2006 H8N4
EPI ISL 189466 HA A/mallard/Sw eden/51671/2006 H8N4
EPI ISL 106124 HA A/duck/Tsukuba/255/2005(H8N5 H8N5
EPI ISL 189328 HA A/mallard/Sw eden/4486/2004 H8N4
EPI ISL 189465 HA A/mallard/Sw eden/541/2002 H8N4
EPI ISL 73373 HA A/mallard/Sw eden/24/2002 H8N4
EPI ISL 73390 HA A/mallard/Sw eden/8/2003 H8N4
EPI ISL 189326 HA A/mallard/Sw eden/2990/2003 H8N4
EPI ISL 189337 HA A/mallard/Sw eden/59517/2007 H8N4
EPI ISL 189330 HA A/mallard/Sw eden/7242/2004 H8N4
EPI ISL 189324 HA A/mallard/Sw eden/3244/2003 H8N4
EPI ISL 189327 HA A/mallard/Sw eden/3240/2003 H8N4
EPI ISL 120214 HA A/garganey/Ukraine/05835-NAMRU3/2006 H8N4
EPI ISL 32601 HA A/Anas crecca/Spain/1459/2008 H8N4
EPI ISL 189322 HA A/mallard/Sw eden/60041/2007 H8N2
EPI ISL 189338 HA A/mallard/Sw eden/68537/2007 A
EPI ISL 96126 HA A/teal/Chany/444/2009 H8N8
EPI ISL 240681 HA A/mallard/Chany/126K-2/2014 H8N4
EPI ISL 66198 HA A/duck/Thailand/SP-355/2007 H8N4
EPI ISL 161002 HA A/ruddy shelduck/Mongolia/593/2010 H8N4
HA A/Chicken/Netherlands/11008325/2011 H8N4
HA A/Turkey/Netherlands/13001007/2013 H8N4
EPI ISL 189340 HA A/mallard/Sw eden/101165/2009 H8N4
EPI ISL 144312 HA A/mallard/Sw eden/100546/2009 H8N4
EPI ISL 33854 HA A/mallard/Netherlands/1/2006 H8N4
EPI ISL 189339 HA A/mallard/Sw eden/99377/2009 H8N4
HA A/Chicken/Netherlands/10009401/2010 H8N4
HA A/Common Teal/Netherlands/12002960/2012 H8N4
HA A/Chicken/Netherlands/11004004/2011 H8N4
EPI ISL 189323 HA A/mallard/Sw eden/4737/2004 A
EPI ISL 23026 HA A/duck/Hokkaido/95/1981 H8N4
EPI ISL 90292 HA A/Turkey/Ontario/6118/1968 H8N4
EPI ISL 3242 HA A/mallard/Alberta/283/1977 H8N4
EPI ISL 11168 HA A/duck/Yangzhou/02/2005 H8N4
EPI ISL 20132 HA A/pintail/Barrow /140/2005 H8N4
EPI ISL 20130 HA A/pintail/Barrow /38/2005 H8N4
EPI ISL 8867 HA A/mallard/Alberta/194/1992 H8N4
EPI ISL 94973 HA A/blue-w inged teal/Guatemala/CIP049-07/2010 H8N4
0.02

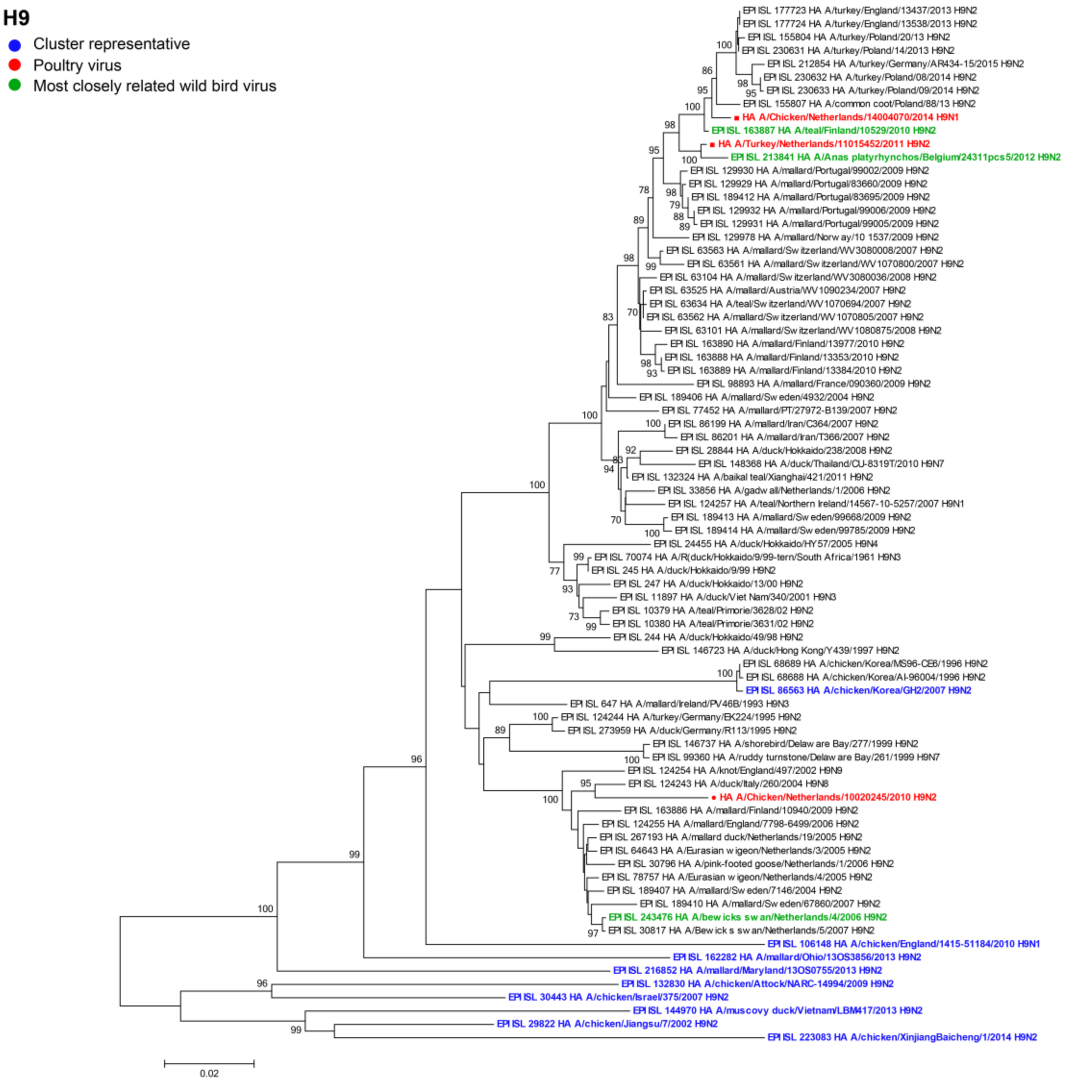
H9
Cluster representative
Poultry virus
Most closely related wild bird virus
EPI ISL 177723 HA A/turkey/England/13437/2013 H9N2
EPI ISL 177724 HA A/turkey/England/13538/2013 H9N2
EPI ISL 155804 HA A/turkey/Poland/20/13 H9N2
EPI ISL 230631 HA A/turkey/Poland/14/2013 H9N2
EPI ISL 212854 HA A/turkey/Germany/AR434-15/2015 H9N2
EPI ISL 230632 HA A/turkey/Poland/08/2014 H9N2
EPI ISL 230633 HA A/turkey/Poland/09/2014 H9N2
EPI ISL 155807 HA A/common coot/Poland/88/13 H9N2
HA A/Chicken/Netherlands/14004070/2014 H9N1
EPI ISL 163887 HA A/teal/Finland/10529/2010 H9N2
HA A/Turkey/Netherlands/11015452/2011 H9N2
EPI ISL 213841 HA A/Anas platyrhynchos/Belgium/24311pcs5/2012 H9N2
EPI ISL 129930 HA A/mallard/Portugal/99002/2009 H9N2
EPI ISL 129929 HA A/mallard/Portugal/83660/2009 H9N2
EPI ISL 189412 HA A/mallard/Portugal/83695/2009 H9N2
EPI ISL 129932 HA A/mallard/Portugal/99006/2009 H9N2
EPI ISL 129931 HA A/mallard/Portugal/99005/2009 H9N2
EPI ISL 129978 HA A/mallard/Norw ay/10 1537/2009 H9N2
EPI ISL 63563 HA A/mallard/Sw itzerland/WV3080008/2007 H9N2
EPI ISL 63561 HA A/mallard/Sw itzerland/WV1070800/2007 H9N2
EPI ISL 63104 HA A/mallard/Sw itzerland/WV3080036/2008 H9N2
EPI ISL 63525 HA A/mallard/Austria/WV1090234/2007 H9N2
EPI ISL 63634 HA A/teal/Sw itzerland/WV1070694/2007 H9N2
EPI ISL 63562 HA A/mallard/Sw itzerland/WV1070805/2007 H9N2
EPI ISL 63101 HA A/mallard/Sw itzerland/WV1080875/2008 H9N2
EPI ISL 163890 HA A/mallard/Finland/13977/2010 H9N2
EPI ISL 163888 HA A/mallard/Finland/13353/2010 H9N2
EPI ISL 163889 HA A/mallard/Finland/13384/2010 H9N2
EPI ISL 98893 HA A/mallard/France/090360/2009 H9N2
EPI ISL 189406 HA A/mallard/Sw eden/4932/2004 H9N2
EPI ISL 77452 HA A/mallard/PT/27972-B139/2007 H9N2
EPI ISL 86199 HA A/mallard/Iran/C364/2007 H9N2
EPI ISL 86201 HA A/mallard/Iran/T366/2007 H9N2
EPI ISL 28844 HA A/duck/Hokkaido/238/2008 H9N2
EPI ISL 148368 HA A/duck/Thailand/CU-8319T/2010 H9N7
EPI ISL 132324 HA A/baikal teal/Xianghai/421/2011 H9N2
EPI ISL 33856 HA A/gadw all/Netherlands/1/2006 H9N2
EPI ISL 124257 HA A/teal/Northern Ireland/14567-10-5257/2007 H9N1
EPI ISL 189413 HA A/mallard/Sw eden/99668/2009 H9N2
EPI ISL 189414 HA A/mallard/Sw eden/99785/2009 H9N2
EPI ISL 24455 HA A/duck/Hokkaido/HY57/2005 H9N4
EPI ISL 70074 HA A/R(duck/Hokkaido/9/99-tern/South Africa/1961 H9N3
EPI ISL 245 HA A/duck/Hokkaido/9/99 H9N2
EPI ISL 247 HA A/duck/Hokkaido/13/00 H9N2
EPI ISL 11897 HA A/duck/Viet Nam/340/2001 H9N3
EPI ISL 10379 HA A/teal/Primorie/3628/02 H9N2
EPI ISL 10380 HA A/teal/Primorie/3631/02 H9N2
EPI ISL 244 HA A/duck/Hokkaido/49/98 H9N2
EPI ISL 146723 HA A/duck/Hong Kong/Y439/1997 H9N2
EPI ISL 68689 HA A/chicken/Korea/MS96-CE6/1996 H9N2
EPI ISL 68688 HA A/chicken/Korea/AI-96004/1996 H9N2
EPI ISL 86563 HA A/chicken/Korea/GH2/2007 H9N2
EPI ISL 647 HA A/mallard/Ireland/PV46B/1993 H9N3
EPI ISL 124244 HA A/turkey/Germany/EK224/1995 H9N2
EPI ISL 273959 HA A/duck/Germany/R113/1995 H9N2
EPI ISL 146737 HA A/shorebird/Delaw are Bay/277/1999 H9N2
EPI ISL 99360 HA A/ruddy turnstone/Delaw are Bay/261/1999 H9N7
EPI ISL 124254 HA A/knot/England/497/2002 H9N9
EPI ISL 124243 HA A/duck/Italy/260/2004 H9N8
HA A/Chicken/Netherlands/10020245/2010 H9N2
EPI ISL 163886 HA A/mallard/Finland/10940/2009 H9N2
EPI ISL 124255 HA A/mallard/England/7798-6499/2006 H9N2
EPI ISL 267193 HA A/mallard duck/Netherlands/19/2005 H9N2
EPI ISL 64643 HA A/Eurasian w igeon/Netherlands/3/2005 H9N2
EPI ISL 30796 HA A/pink-footed goose/Netherlands/1/2006 H9N2
EPI ISL 78757 HA A/Eurasian w igeon/Netherlands/4/2005 H9N2
EPI ISL 189407 HA A/mallard/Sw eden/7146/2004 H9N2
EPI ISL 189410 HA A/mallard/Sw eden/67860/2007 H9N2
EPI ISL 243476 HA A/bew icks sw an/Netherlands/4/2006 H9N2
EPI ISL 30817 HA A/Bew ick s sw an/Netherlands/5/2007 H9N2
EPI ISL 106148 HA A/chicken/England/1415-51184/2010 H9N1
EPI ISL 162282 HA A/mallard/Ohio/13OS3856/2013 H9N2
EPI ISL 216852 HA A/mallard/Maryland/13OS0755/2013 H9N2
EPI ISL 132830 HA A/chicken/Attock/NARC-14994/2009 H9N2
EPI ISL 30443 HA A/chicken/Israel/375/2007 H9N2
EPI ISL 144970 HA A/muscovy duck/Vietnam/LBM417/2013 H9N2
EPI ISL 29822 HA A/chicken/Jiangsu/7/2002 H9N2
EPI ISL 223083 HA A/chicken/XinjiangBaicheng/1/2014 H9N2
0.02

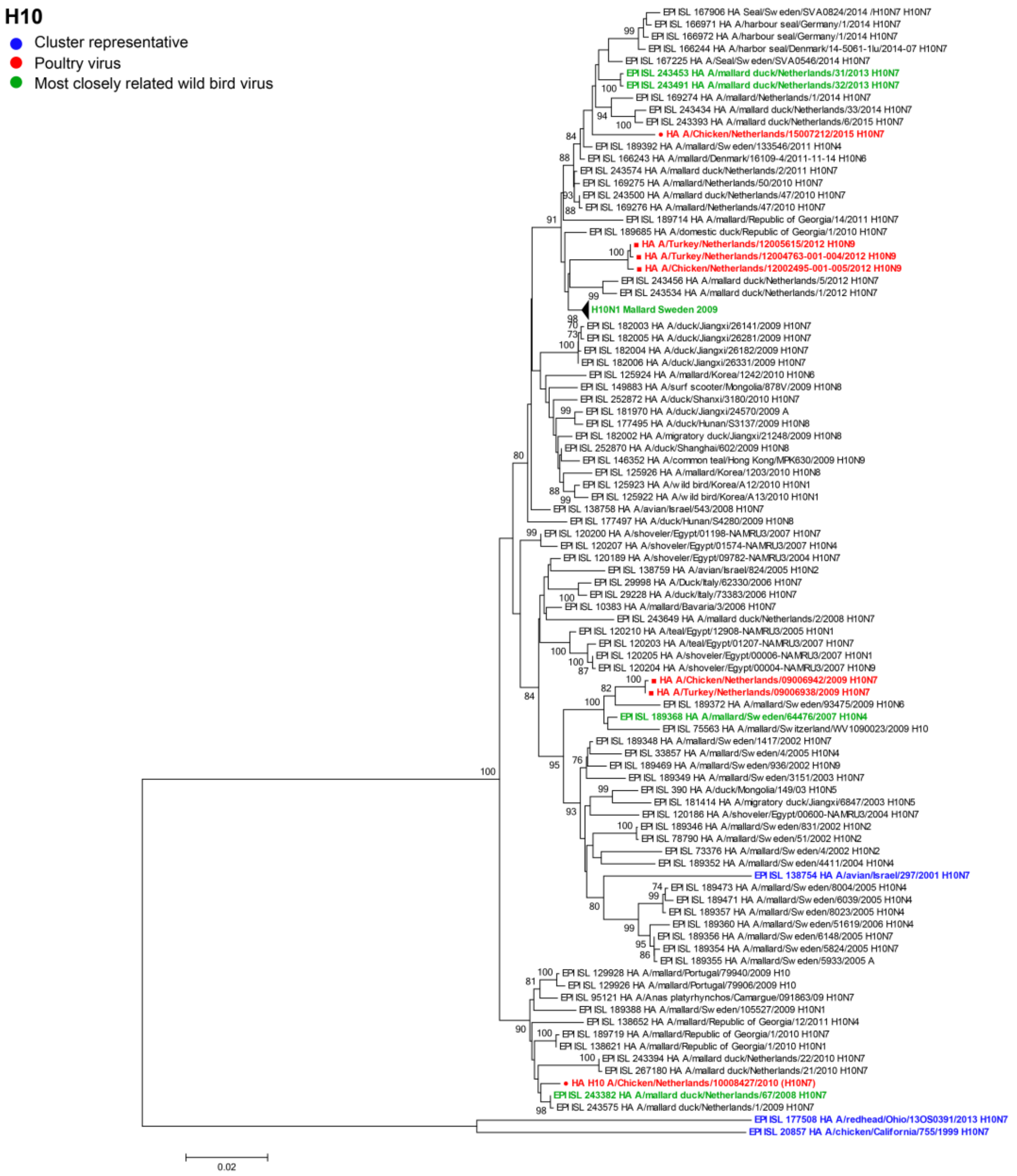
H10
Cluster representative
Poultry virus
Most closely related wild bird virus
EPI ISL 167906 HA Seal/Sw eden/SVA0824/2014 /H10N7 H10N7
EPI ISL 166971 HA A/harbour seal/Germany/1/2014 H10N7
EPI ISL 166972 HA A/harbour seal/Germany/1/2014 H10N7
EPI ISL 166244 HA A/harbor seal/Denmark/14-5061-1lu/2014-07 H10N7
EPI ISL 167225 HA A/Seal/Sw eden/SVA0546/2014 H10N7
EPI ISL 243453 HA A/mallard duck/Netherlands/31/2013 H10N7
EPI ISL 243491 HA A/mallard duck/Netherlands/32/2013 H10N7
EPI ISL 169274 HA A/mallard/Netherlands/1/2014 H10N7
EPI ISL 243434 HA A/mallard duck/Netherlands/33/2014 H10N7
EPI ISL 243393 HA A/mallard duck/Netherlands/6/2015 H10N7
HA A/Chicken/Netherlands/15007212/2015 H10N7
EPI ISL 189392 HA A/mallard/Sw eden/133546/2011 H10N4
EPI ISL 166243 HA A/mallard/Denmark/16109-4/2011-11-14 H10N6
EPI ISL 243574 HA A/mallard duck/Netherlands/2/2011 H10N7
EPI ISL 169275 HA A/mallard/Netherlands/50/2010 H10N7
EPI ISL 243500 HA A/mallard duck/Netherlands/47/2010 H10N7
EPI ISL 169276 HA A/mallard/Netherlands/47/2010 H10N7
EPI ISL 189714 HA A/mallard/Republic of Georgia/14/2011 H10N7
EPI ISL 189685 HA A/domestic duck/Republic of Georgia/1/2010 H10N7
HA A/Turkey/Netherlands/12005615/2012 H10N9
HA A/Turkey/Netherlands/12004763-001-004/2012 H10N9
HA A/Chicken/Netherlands/12002495-001-005/2012 H10N9
EPI ISL 243456 HA A/mallard duck/Netherlands/5/2012 H10N7
EPI ISL 243534 HA A/mallard duck/Netherlands/1/2012 H10N7
H10N1 Mallard Sweden 2009
EPI ISL 182003 HA A/duck/Jiangxi/26141/2009 H10N7
EPI ISL 182005 HA A/duck/Jiangxi/26281/2009 H10N7
EPI ISL 182004 HA A/duck/Jiangxi/26182/2009 H10N7
EPI ISL 182006 HA A/duck/Jiangxi/26331/2009 H10N7
EPI ISL 125924 HA A/mallard/Korea/1242/2010 H10N6
EPI ISL 149883 HA A/surf scooter/Mongolia/878V/2009 H10N8
EPI ISL 252872 HA A/duck/Shanxi/3180/2010 H10N7
EPI ISL 181970 HA A/duck/Jiangxi/24570/2009 A
EPI ISL 177495 HA A/duck/Hunan/S3137/2009 H10N8
EPI ISL 182002 HA A/migratory duck/Jiangxi/21248/2009 H10N8
EPI ISL 252870 HA A/duck/Shanghai/602/2009 H10N8
EPI ISL 146352 HA A/common teal/Hong Kong/MPK630/2009 H10N9
EPI ISL 125926 HA A/mallard/Korea/1203/2010 H10N8
EPI ISL 125923 HA A/w ild bird/Korea/A12/2010 H10N1
EPI ISL 125922 HA A/w ild bird/Korea/A13/2010 H10N1
EPI ISL 138758 HA A/avian/Israel/543/2008 H10N7
EPI ISL 177497 HA A/duck/Hunan/S4280/2009 H10N8
EPI ISL 120200 HA A/shoveler/Egypt/01198-NAMRU3/2007 H10N7
EPI ISL 120207 HA A/shoveler/Egypt/01574-NAMRU3/2007 H10N4
EPI ISL 120189 HA A/shoveler/Egypt/09782-NAMRU3/2004 H10N7
EPI ISL 138759 HA A/avian/Israel/824/2005 H10N2
EPI ISL 29998 HA A/Duck/Italy/62330/2006 H10N7
EPI ISL 29228 HA A/duck/Italy/73383/2006 H10N7
EPI ISL 10383 HA A/mallard/Bavaria/3/2006 H10N7
EPI ISL 243649 HA A/mallard duck/Netherlands/2/2008 H10N7
EPI ISL 120210 HA A/teal/Egypt/12908-NAMRU3/2005 H10N1
EPI ISL 120203 HA A/teal/Egypt/01207-NAMRU3/2007 H10N7
EPI ISL 120205 HA A/shoveler/Egypt/00006-NAMRU3/2007 H10N1
EPI ISL 120204 HA A/shoveler/Egypt/00004-NAMRU3/2007 H10N9
HA A/Chicken/Netherlands/09006942/2009 H10N7
HA A/Turkey/Netherlands/09006938/2009 H10N7
EPI ISL 189372 HA A/mallard/Sw eden/93475/2009 H10N6
EPI ISL 189368 HA A/mallard/Sw eden/64476/2007 H10N4
EPI ISL 75563 HA A/mallard/Sw itzerland/WV1090023/2009 H10
EPI ISL 189348 HA A/mallard/Sw eden/1417/2002 H10N7
EPI ISL 33857 HA A/mallard/Sw eden/4/2005 H10N4
EPI ISL 189469 HA A/mallard/Sw eden/936/2002 H10N9
EPI ISL 189349 HA A/mallard/Sw eden/3151/2003 H10N7
EPI ISL 390 HA A/duck/Mongolia/149/03 H10N5
EPI ISL 181414 HA A/migratory duck/Jiangxi/6847/2003 H10N5
EPI ISL 120186 HA A/shoveler/Egypt/00600-NAMRU3/2004 H10N7
EPI ISL 189346 HA A/mallard/Sw eden/831/2002 H10N2
EPI ISL 78790 HA A/mallard/Sw eden/51/2002 H10N2
EPI ISL 73376 HA A/mallard/Sw eden/4/2002 H10N2
EPI ISL 189352 HA A/mallard/Sw eden/4411/2004 H10N4
EPI ISL 138754 HA A/avian/Israel/297/2001 H10N7
EPI ISL 189473 HA A/mallard/Sw eden/8004/2005 H10N4
EPI ISL 189471 HA A/mallard/Sw eden/6039/2005 H10N4
EPI ISL 189357 HA A/mallard/Sw eden/8023/2005 H10N4
EPI ISL 189360 HA A/mallard/Sw eden/51619/2006 H10N4
EPI ISL 189356 HA A/mallard/Sw eden/6148/2005 H10N7
EPI ISL 189354 HA A/mallard/Sw eden/5824/2005 H10N7
EPI ISL 189355 HA A/mallard/Sw eden/5933/2005 A
EPI ISL 129928 HA A/mallard/Portugal/79940/2009 H10
EPI ISL 129926 HA A/mallard/Portugal/79906/2009 H10
EPI ISL 95121 HA A/Anas platyrhynchos/Camargue/091863/09 H10N7
EPI ISL 189388 HA A/mallard/Sw eden/105527/2009 H10N1
EPI ISL 138652 HA A/mallard/Republic of Georgia/12/2011 H10N4
EPI ISL 189719 HA A/mallard/Republic of Georgia/1/2010 H10N7
EPI ISL 138621 HA A/mallard/Republic of Georgia/1/2010 H10N1
EPI ISL 243394 HA A/mallard duck/Netherlands/22/2010 H10N7
EPI ISL 267180 HA A/mallard duck/Netherlands/21/2010 H10N7
HA H10 A/Chicken/Netherlands/10008427/2010 (H10N7)
EPI ISL 243382 HA A/mallard duck/Netherlands/67/2008 H10N7
EPI ISL 243575 HA A/mallard duck/Netherlands/1/2009 H10N7
EPI ISL 177508 HA A/redhead/Ohio/13OS0391/2013 H10N7
EPI ISL 20857 HA A/chicken/California/755/1999 H10N7
0.02

Supplementary Figure S2. Phylogenetic trees of NA genes.

Neighbour joining (NJ) phylogenetic trees of the neuraminidase (NA) gene segments of low pathogenic avian influenza (LPAI) viruses from poultry, detected as part of the national avian influenza (AI) surveillance program in the Netherlands, January 2006-September 2016. Nucleotide (nt) sequences of cluster representatives, poultry viruses and top 50 BLAST hits were aligned for each NA gene segment separately: N1 (1221 nt), N2 (1245 nt), N3 (1343 nt), N4 (1337 nt), N5 (1232 nt), N7 (1225 nt), N8 (1376 nt) and N9 (1342 nt). Phylogenetic trees were generated using the Tamura-Nei substitution model with a gamma distribution (shape parameter = 1) for rate variation within the MEGA7 software package. Bootstrap support values above 70 (1,000 replicates) are shown at the branches. Colours represent cluster representatives (blue), poultry viruses (red), and most identical wild bird viruses as determined by BLAST (red). Symbols represent poultry viruses most identical to wild bird viruses isolated in the Netherlands (dots), other European countries (squares) and Asia (triangles). We gratefully acknowledge the authors, originating and submitting laboratories of the sequences from GISAID's EpiFlu database [28] on which this research is based. All submitters of data may be contacted directly via the GISAID website (http://www.gisaid.org).

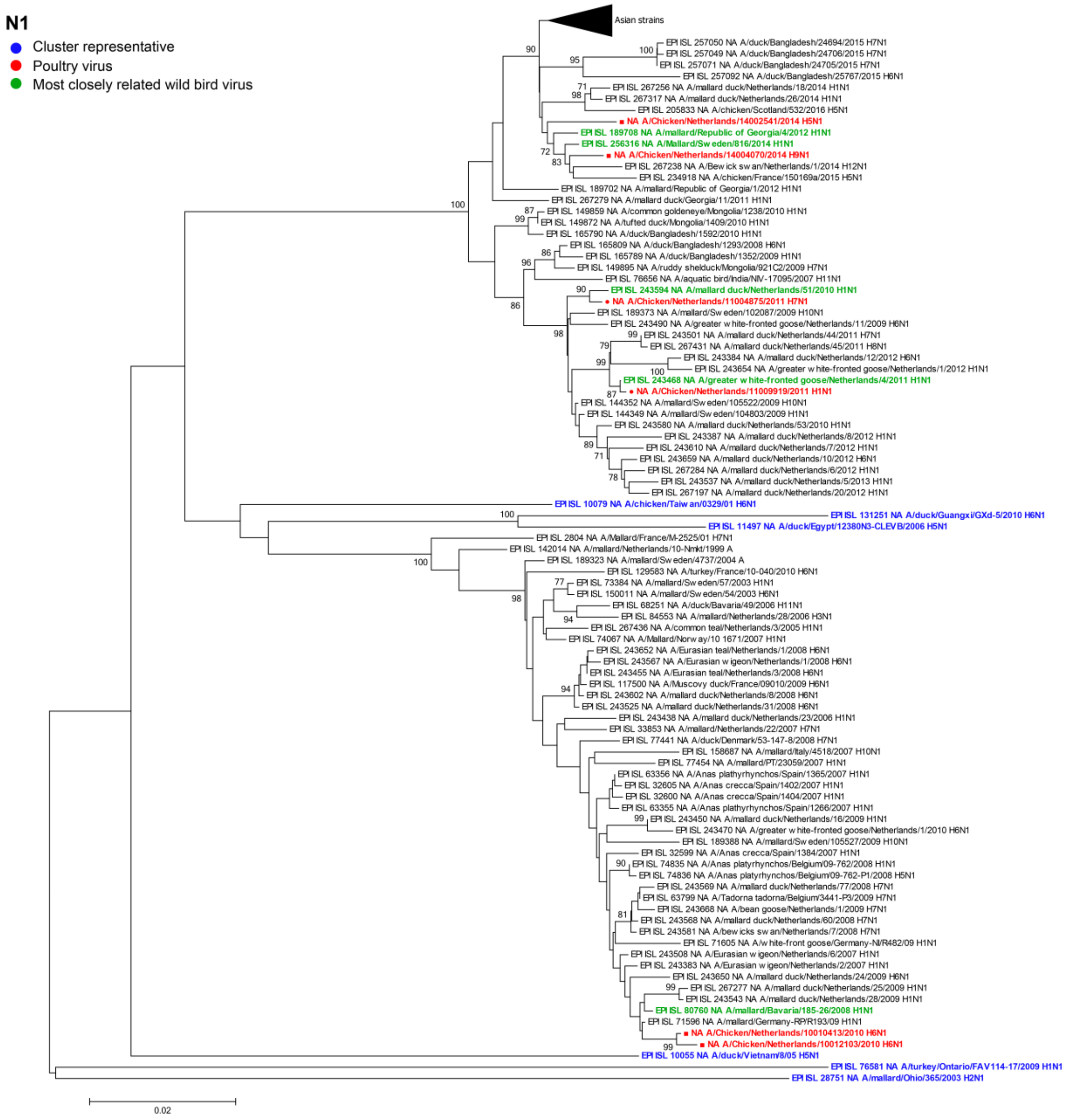
N1
Cluster representative
Poultry virus
Most closely related wild bird virus
Asian strains
EPI ISL 257050 NA A/duck/Bangladesh/24694/2015 H7N1
EPI ISL 257049 NA A/duck/Bangladesh/24706/2015 H7N1
EPI ISL 257071 NA A/duck/Bangladesh/24705/2015 H7N1
EPI ISL 257092 NA A/duck/Bangladesh/25767/2015 H6N1
EPI ISL 267256 NA A/mallard duck/Netherlands/18/2014 H1N1
EPI ISL 267317 NA A/mallard duck/Netherlands/26/2014 H1N1
EPI ISL 205833 NA A/chicken/Scotland/532/2016 H5N1
NA A/Chicken/Netherlands/14002541/2014 H5N1
EPI ISL 189708 NA A/mallard/Republic of Georgia/4/2012 H1N1
EPI ISL 256316 NA A/Mallard/Sweden/816/2014 H1N1
NA A/Chicken/Netherlands/14004070/2014 H9N1
EPI ISL 267238 NA A/Bewick swan/Netherlands/1/2014 H12N1
EPI ISL 234918 NA A/chicken/France/150169a/2015 H5N1
EPI ISL 189702 NA A/mallard/Republic of Georgia/1/2012 H1N1
EPI ISL 267279 NA A/mallard duck/Georgia/11/2011 H1N1
EPI ISL 149859 NA A/common goldeneye/Mongolia/1238/2010 H1N1
EPI ISL 149872 NA A/tufted duck/Mongolia/1409/2010 H1N1
EPI ISL 165790 NA A/duck/Bangladesh/1592/2010 H1N1
EPI ISL 165809 NA A/duck/Bangladesh/1293/2008 H6N1
EPI ISL 165789 NA A/duck/Bangladesh/1352/2009 H1N1
EPI ISL 149895 NA A/ruddy shelduck/Mongolia/921C2/2009 H7N1
EPI ISL 76656 NA A/aquatic bird/India/NIV-17095/2007 H11N1
EPI ISL 243594 NA A/mallard duck/Netherlands/51/2010 H1N1
NA A/Chicken/Netherlands/11004875/2011 H7N1
EPI ISL 189373 NA A/mallard/Sweden/102087/2009 H10N1
EPI ISL 243490 NA A/greater white-fronted goose/Netherlands/11/2009 H6N1
EPI ISL 243501 NA A/mallard duck/Netherlands/44/2011 H7N1
EPI ISL 267431 NA A/mallard duck/Netherlands/45/2011 H8N1
EPI ISL 243384 NA A/mallard duck/Netherlands/12/2012 H6N1
EPI ISL 243654 NA A/greater white-fronted goose/Netherlands/1/2012 H1N1
EPI ISL 243468 NA A/greater white-fronted goose/Netherlands/4/2011 H1N1
NA A/Chicken/Netherlands/11009919/2011 H1N1
EPI ISL 144352 NA A/mallard/Sweden/105522/2009 H10N1
EPI ISL 144349 NA A/mallard/Sweden/104803/2009 H1N1
EPI ISL 243580 NA A/mallard duck/Netherlands/53/2010 H1N1
EPI ISL 243387 NA A/mallard duck/Netherlands/8/2012 H1N1
EPI ISL 243610 NA A/mallard duck/Netherlands/7/2012 H1N1
EPI ISL 243659 NA A/mallard duck/Netherlands/10/2012 H6N1
EPI ISL 267284 NA A/mallard duck/Netherlands/6/2012 H1N1
EPI ISL 243537 NA A/mallard duck/Netherlands/5/2013 H1N1
EPI ISL 267197 NA A/mallard duck/Netherlands/20/2012 H1N1
EPI ISL 10079 NA A/chicken/Taiwan/0329/01 H6N1
EPI ISL 131251 NA A/duck/Guangxi/GXd-5/2010 H6N1
EPI ISL 11497 NA A/duck/Egypt/12380N3-CLEVB/2006 H5N1
EPI ISL 2804 NA A/Mallard/France/M-2525/01 H7N1
EPI ISL 142014 NA A/mallard/Netherlands/10-Nmkt/1999 A
EPI ISL 189323 NA A/mallard/Sweden/4737/2004 A
EPI ISL 129583 NA A/turkey/France/10-040/2010 H6N1
EPI ISL 73384 NA A/mallard/Sweden/57/2003 H1N1
EPI ISL 150011 NA A/mallard/Sweden/54/2003 H6N1
EPI ISL 68251 NA A/duck/Bavaria/49/2006 H11N1
EPI ISL 84553 NA A/mallard/Netherlands/28/2006 H3N1
EPI ISL 267436 NA A/common teal/Netherlands/3/2005 H1N1
EPI ISL 74067 NA A/Mallard/Norway/10 1671/2007 H1N1
EPI ISL 243652 NA A/Eurasian teal/Netherlands/1/2008 H6N1
EPI ISL 243567 NA A/Eurasian wigeon/Netherlands/1/2008 H6N1
EPI ISL 243455 NA A/Eurasian teal/Netherlands/3/2008 H6N1
EPI ISL 117500 NA A/Muscovy duck/France/09010/2009 H6N1
EPI ISL 243602 NA A/mallard duck/Netherlands/8/2008 H6N1
EPI ISL 243525 NA A/mallard duck/Netherlands/31/2008 H6N1
EPI ISL 243438 NA A/mallard duck/Netherlands/23/2006 H1N1
EPI ISL 33853 NA A/mallard/Netherlands/22/2007 H7N1
EPI ISL 77441 NA A/duck/Denmark/53-147-8/2008 H7N1
EPI ISL 158687 NA A/mallard/Italy/4518/2007 H10N1
EPI ISL 77454 NA A/mallard/PT/23059/2007 H1N1
EPI ISL 63356 NA A/Anas plathyrhynchos/Spain/1365/2007 H1N1
EPI ISL 32605 NA A/Anas crecca/Spain/1402/2007 H1N1
EPI ISL 32600 NA A/Anas crecca/Spain/1404/2007 H1N1
EPI ISL 63355 NA A/Anas plathyrhynchos/Spain/1266/2007 H1N1
EPI ISL 243450 NA A/mallard duck/Netherlands/16/2009 H1N1
EPI ISL 243470 NA A/greater white-fronted goose/Netherlands/1/2010 H6N1
EPI ISL 189388 NA A/mallard/Sweden/105527/2009 H10N1
EPI ISL 32599 NA A/Anas crecca/Spain/1384/2007 H1N1
EPI ISL 74835 NA A/Anas platyrhynchos/Belgium/09-762/2008 H1N1
EPI ISL 74836 NA A/Anas platyrhynchos/Belgium/09-762-P1/2008 H5N1
EPI ISL 243569 NA A/mallard duck/Netherlands/77/2008 H7N1
EPI ISL 63799 NA A/Tadorna tadorna/Belgium/3441-P3/2009 H7N1
EPI ISL 243668 NA A/bean goose/Netherlands/1/2009 H7N1
EPI ISL 243568 NA A/mallard duck/Netherlands/60/2008 H7N1
EPI ISL 243581 NA A/bewicks swan/Netherlands/7/2008 H7N1
EPI ISL 71605 NA A/white-front goose/Germany-NI/R482/09 H1N1
EPI ISL 243508 NA A/Eurasian wigeon/Netherlands/6/2007 H1N1
EPI ISL 243383 NA A/Eurasian wigeon/Netherlands/2/2007 H1N1
EPI ISL 243650 NA A/mallard duck/Netherlands/24/2009 H6N1
EPI ISL 267277 NA A/mallard duck/Netherlands/25/2009 H1N1
EPI ISL 243543 NA A/mallard duck/Netherlands/28/2009 H1N1
EPI ISL 80760 NA A/mallard/Bavaria/185-26/2008 H1N1
EPI ISL 71596 NA A/mallard/Germany-RP/R193/09 H1N1
NA A/Chicken/Netherlands/10010413/2010 H6N1
NA A/Chicken/Netherlands/10012103/2010 H6N1
EPI ISL 10055 NA A/duck/Vietnam/8/05 H5N1
EPI ISL 76581 NA A/turkey/Ontario/FAV114-17/2009 H1N1
EPI ISL 28751 NA A/mallard/Ohio/365/2003 H2N1
0.02

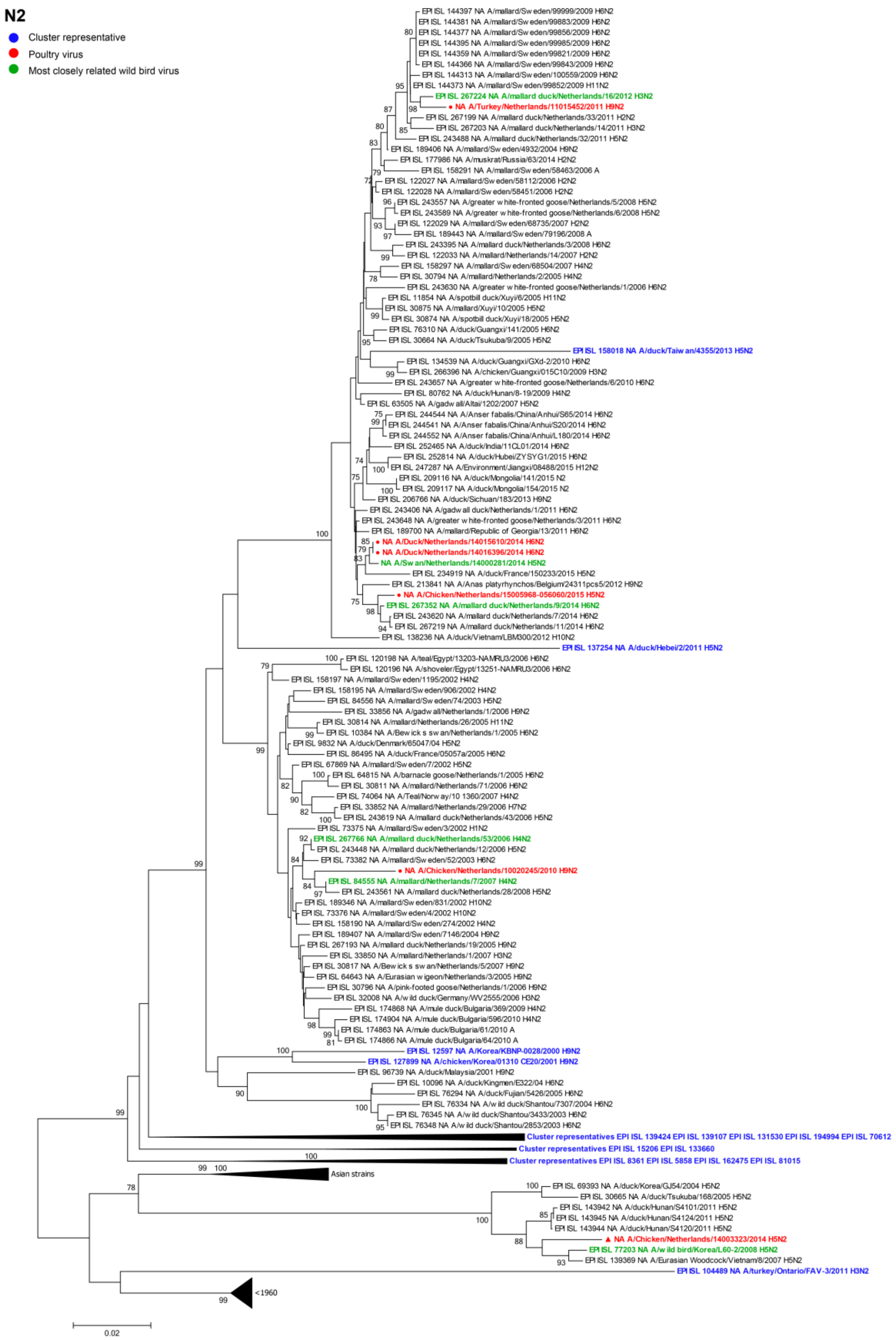
N2
Cluster representative
Poultry virus
Most closely related wild bird virus
EPI ISL 144397 NA A/mallard/Sweden/99999/2009 H6N2
EPI ISL 144381 NA A/mallard/Sweden/99883/2009 H6N2
EPI ISL 144377 NA A/mallard/Sweden/99856/2009 H6N2
EPI ISL 144395 NA A/mallard/Sweden/99985/2009 H6N2
EPI ISL 144359 NA A/mallard/Sweden/99821/2009 H6N2
EPI ISL 144366 NA A/mallard/Sweden/99843/2009 H6N2
EPI ISL 144313 NA A/mallard/Sweden/100559/2009 H6N2
EPI ISL 144373 NA A/mallard/Sweden/99852/2009 H11N2
EPI ISL 267224 NA A/mallard duck/Netherlands/16/2012 H3N2
NA A/Turkey/Netherlands/11015452/2011 H9N2
EPI ISL 267199 NA A/mallard duck/Netherlands/33/2011 H2N2
EPI ISL 267203 NA A/mallard duck/Netherlands/14/2011 H3N2
EPI ISL 243488 NA A/mallard duck/Netherlands/32/2011 H5N2
EPI ISL 189406 NA A/mallard/Sweden/4932/2004 H9N2
EPI ISL 177986 NA A/muskrat/Russia/63/2014 H2N2
EPI ISL 158291 NA A/mallard/Sweden/58463/2006 A
EPI ISL 122027 NA A/mallard/Sweden/58112/2006 H2N2
EPI ISL 122028 NA A/mallard/Sweden/58451/2006 H2N2
EPI ISL 243557 NA A/greater white-fronted goose/Netherlands/5/2008 H5N2
EPI ISL 243589 NA A/greater white-fronted goose/Netherlands/6/2008 H5N2
EPI ISL 122029 NA A/mallard/Sweden/68735/2007 H2N2
EPI ISL 189443 NA A/mallard/Sweden/79196/2008 A
EPI ISL 243395 NA A/mallard duck/Netherlands/3/2008 H6N2
EPI ISL 122033 NA A/mallard/Netherlands/14/2007 H2N2
EPI ISL 158297 NA A/mallard/Sweden/68504/2007 H4N2
EPI ISL 30794 NA A/mallard/Netherlands/2/2005 H4N2
EPI ISL 243630 NA A/greater white-fronted goose/Netherlands/1/2006 H6N2
EPI ISL 11854 NA A/spotbill duck/Xuyi/6/2005 H11N2
EPI ISL 30875 NA A/mallard/Xuyi/10/2005 H5N2
EPI ISL 30874 NA A/spotbill duck/Xuyi/18/2005 H5N2
EPI ISL 76310 NA A/duck/Guangxi/141/2005 H6N2
EPI ISL 30664 NA A/duck/Tsukuba/9/2005 H5N2
EPI ISL 158018 NA A/duck/Taiwan/4355/2013 H5N2
EPI ISL 134539 NA A/duck/Guangxi/GXd-2/2010 H6N2
EPI ISL 266396 NA A/chicken/Guangxi/015C10/2009 H3N2
EPI ISL 243657 NA A/greater white-fronted goose/Netherlands/6/2010 H6N2
EPI ISL 80762 NA A/duck/Hunan/8-19/2009 H4N2
EPI ISL 63505 NA A/gadwall/Altai/1202/2007 H5N2
EPI ISL 244544 NA A/Anser fabalis/China/Anhui/S65/2014 H6N2
EPI ISL 244541 NA A/Anser fabalis/China/Anhui/S20/2014 H6N2
EPI ISL 244552 NA A/Anser fabalis/China/Anhui/L180/2014 H6N2
EPI ISL 252465 NA A/duck/India/11CL01/2014 H6N2
EPI ISL 252814 NA A/duck/Hubei/ZYSYG1/2015 H6N2
EPI ISL 247287 NA A/Environment/Jiangxi/08488/2015 H12N2
EPI ISL 209116 NA A/duck/Mongolia/141/2015 N2
EPI ISL 209117 NA A/duck/Mongolia/154/2015 N2
EPI ISL 206766 NA A/duck/Sichuan/183/2013 H9N2
EPI ISL 243406 NA A/gadwall duck/Netherlands/1/2011 H6N2
EPI ISL 243648 NA A/greater white-fronted goose/Netherlands/3/2011 H6N2
EPI ISL 189700 NA A/mallard/Republic of Georgia/13/2011 H6N2
NA A/Duck/Netherlands/14015610/2014 H6N2
NA A/Duck/Netherlands/14016396/2014 H6N2
NA A/Swan/Netherlands/14000281/2014 H5N2
EPI ISL 234919 NA A/duck/France/150233/2015 H5N2
EPI ISL 213841 NA A/Anas platyrhynchos/Belgium/24311pcs5/2012 H9N2
NA A/Chicken/Netherlands/15005968-056060/2015 H5N2
EPI ISL 267352 NA A/mallard duck/Netherlands/9/2014 H6N2
EPI ISL 243620 NA A/mallard duck/Netherlands/7/2014 H6N2
EPI ISL 267219 NA A/mallard duck/Netherlands/11/2014 H6N2
EPI ISL 138236 NA A/duck/Vietnam/LBM300/2012 H10N2
EPI ISL 137254 NA A/duck/Hebei/2/2011 H5N2
EPI ISL 120198 NA A/teal/Egypt/13203-NAMRU3/2006 H6N2
EPI ISL 120196 NA A/shoveler/Egypt/13251-NAMRU3/2006 H6N2
EPI ISL 158197 NA A/mallard/Sweden/1195/2002 H4N2
EPI ISL 158195 NA A/mallard/Sweden/906/2002 H4N2
EPI ISL 84556 NA A/mallard/Sweden/74/2003 H5N2
EPI ISL 33856 NA A/gadwall/Netherlands/1/2006 H9N2
EPI ISL 30814 NA A/mallard/Netherlands/26/2005 H11N2
EPI ISL 10384 NA A/Bewicks swan/Netherlands/1/2005 H6N2
EPI ISL 9832 NA A/duck/Denmark/65047/04 H5N2
EPI ISL 86495 NA A/duck/France/05057a/2005 H6N2
EPI ISL 67869 NA A/mallard/Sweden/7/2002 H5N2
EPI ISL 64815 NA A/barnacle goose/Netherlands/1/2005 H6N2
EPI ISL 30811 NA A/mallard/Netherlands/71/2006 H6N2
EPI ISL 74064 NA A/Teal/Norway/10 1360/2007 H4N2
EPI ISL 33852 NA A/mallard/Netherlands/29/2006 H7N2
EPI ISL 243619 NA A/mallard duck/Netherlands/43/2006 H5N2
EPI ISL 73375 NA A/mallard/Sweden/3/2002 H1N2
EPI ISL 267766 NA A/mallard duck/Netherlands/53/2006 H4N2
EPI ISL 243448 NA A/mallard duck/Netherlands/12/2006 H5N2
EPI ISL 73382 NA A/mallard/Sweden/52/2003 H6N2
NA A/Chicken/Netherlands/10020245/2010 H9N2
EPI ISL 84555 NA A/mallard/Netherlands/7/2007 H4N2
EPI ISL 243561 NA A/mallard duck/Netherlands/28/2008 H5N2
EPI ISL 189346 NA A/mallard/Sweden/831/2002 H10N2
EPI ISL 73376 NA A/mallard/Sweden/4/2002 H10N2
EPI ISL 158190 NA A/mallard/Sweden/274/2002 H4N2
EPI ISL 189407 NA A/mallard/Sweden/7146/2004 H9N2
EPI ISL 267193 NA A/mallard duck/Netherlands/19/2005 H9N2
EPI ISL 33850 NA A/mallard/Netherlands/1/2007 H3N2
EPI ISL 30817 NA A/Bewicks swan/Netherlands/5/2007 H9N2
EPI ISL 64643 NA A/Eurasian wigeon/Netherlands/3/2005 H9N2
EPI ISL 30796 NA A/pink-footed goose/Netherlands/1/2006 H9N2
EPI ISL 32008 NA A/wild duck/Germany/WV2555/2006 H3N2
EPI ISL 174868 NA A/mule duck/Bulgaria/369/2009 H4N2
EPI ISL 174904 NA A/mule duck/Bulgaria/596/2010 H4N2
EPI ISL 174863 NA A/mule duck/Bulgaria/61/2010 A
EPI ISL 174866 NA A/mule duck/Bulgaria/64/2010 A
EPI ISL 12597 NA A/Korea/KBNP-0028/2000 H9N2
EPI ISL 127899 NA A/chicken/Korea/01310 CE20/2001 H9N2
EPI ISL 96739 NA A/duck/Malaysia/2001 H9N2
EPI ISL 10096 NA A/duck/Kingmen/E322/04 H6N2
EPI ISL 76294 NA A/duck/Fujian/5426/2005 H6N2
EPI ISL 76334 NA A/wild duck/Shantou/7307/2004 H6N2
EPI ISL 76345 NA A/wild duck/Shantou/3433/2003 H6N2
EPI ISL 76348 NA A/wild duck/Shantou/2853/2003 H6N2
Cluster representatives EPI ISL 139424 EPI ISL 139107 EPI ISL 131530 EPI ISL 194994 EPI ISL 70612
Cluster representatives EPI ISL 15206 EPI ISL 133660
Cluster representatives EPI ISL 8361 EPI ISL 5858 EPI ISL 162475 EPI ISL 81015
Asian strains
EPI ISL 69393 NA A/duck/Korea/GJ54/2004 H5N2
EPI ISL 30665 NA A/duck/Tsukuba/168/2005 H5N2
EPI ISL 143942 NA A/duck/Hunan/S4101/2011 H5N2
EPI ISL 143945 NA A/duck/Hunan/S4124/2011 H5N2
EPI ISL 143944 NA A/duck/Hunan/S4120/2011 H5N2
NA A/Chicken/Netherlands/14003323/2014 H5N2
EPI ISL 77203 NA A/wild bird/Korea/L60-2/2008 H5N2
EPI ISL 139369 NA A/Eurasian Woodcock/Vietnam/8/2007 H5N2
EPI ISL 104489 NA A/turkey/Ontario/FAV-3/2011 H3N2
<1960
0.02

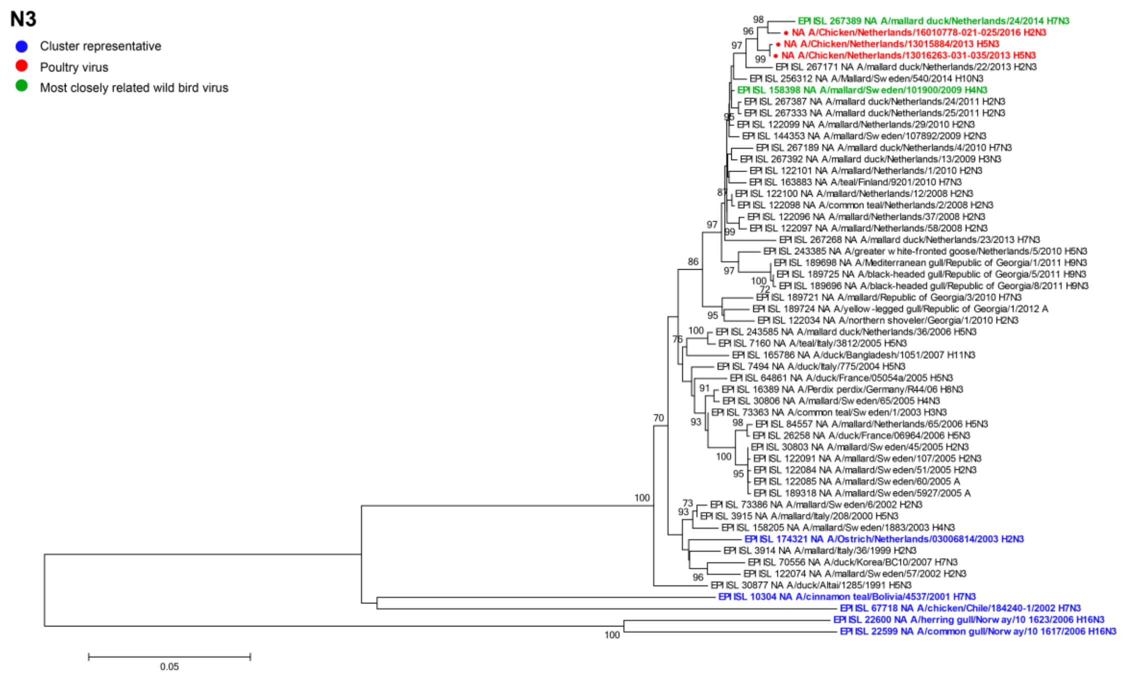
N3
Cluster representative
Poultry virus
Most closely related wild bird virus
EPI ISL 267389 NA A/mallard duck/Netherlands/24/2014 H7N3
NA A/Chicken/Netherlands/16010778-021-025/2016 H2N3
NA A/Chicken/Netherlands/13015884/2013 H5N3
NA A/Chicken/Netherlands/13016263-031-035/2013 H5N3
EPI ISL 267171 NA A/mallard duck/Netherlands/22/2013 H2N3
EPI ISL 256312 NA A/Mallard/Sw eden/540/2014 H10N3
EPI ISL 158398 NA A/mallard/Sw eden/101900/2009 H4N3
EPI ISL 267387 NA A/mallard duck/Netherlands/24/2011 H2N3
EPI ISL 267333 NA A/mallard duck/Netherlands/25/2011 H2N3
EPI ISL 122099 NA A/mallard/Netherlands/29/2010 H2N3
EPI ISL 144353 NA A/mallard/Sw eden/107892/2009 H2N3
EPI ISL 267189 NA A/mallard duck/Netherlands/4/2010 H7N3
EPI ISL 267392 NA A/mallard duck/Netherlands/13/2009 H3N3
EPI ISL 122101 NA A/mallard/Netherlands/1/2010 H2N3
EPI ISL 163883 NA A/teal/Finland/9201/2010 H7N3
EPI ISL 122100 NA A/mallard/Netherlands/12/2008 H2N3
EPI ISL 122098 NA A/common teal/Netherlands/2/2008 H2N3
EPI ISL 122096 NA A/mallard/Netherlands/37/2008 H2N3
EPI ISL 122097 NA A/mallard/Netherlands/58/2008 H2N3
EPI ISL 267268 NA A/mallard duck/Netherlands/23/2013 H7N3
EPI ISL 243385 NA A/greater w hite-fronted goose/Netherlands/5/2010 H5N3
EPI ISL 189698 NA A/Mediterranean gull/Republic of Georgia/1/2011 H9N3
EPI ISL 189725 NA A/black-headed gull/Republic of Georgia/5/2011 H9N3
EPI ISL 189696 NA A/black-headed gull/Republic of Georgia/8/2011 H9N3
EPI ISL 189721 NA A/mallard/Republic of Georgia/3/2010 H7N3
EPI ISL 189724 NA A/yellow -legged gull/Republic of Georgia/1/2012 A
EPI ISL 122034 NA A/northern shoveler/Georgia/1/2010 H2N3
EPI ISL 243585 NA A/mallard duck/Netherlands/36/2006 H5N3
EPI ISL 7160 NA A/teal/Italy/3812/2005 H5N3
EPI ISL 165786 NA A/duck/Bangladesh/1051/2007 H11N3
EPI ISL 7494 NA A/duck/Italy/775/2004 H5N3
EPI ISL 64861 NA A/duck/France/05054a/2005 H5N3
EPI ISL 16389 NA A/Perdix perdix/Germany/R44/06 H8N3
EPI ISL 30806 NA A/mallard/Sw eden/65/2005 H4N3
EPI ISL 73363 NA A/common teal/Sw eden/1/2003 H3N3
EPI ISL 84557 NA A/mallard/Netherlands/65/2006 H5N3
EPI ISL 26258 NA A/duck/France/06964/2006 H5N3
EPI ISL 30803 NA A/mallard/Sw eden/45/2005 H2N3
EPI ISL 122091 NA A/mallard/Sw eden/107/2005 H2N3
EPI ISL 122084 NA A/mallard/Sw eden/51/2005 H2N3
EPI ISL 122085 NA A/mallard/Sw eden/60/2005 A
EPI ISL 189318 NA A/mallard/Sw eden/5927/2005 A
EPI ISL 73386 NA A/mallard/Sw eden/6/2002 H2N3
EPI ISL 3915 NA A/mallard/Italy/208/2000 H5N3
EPI ISL 158205 NA A/mallard/Sw eden/1883/2003 H4N3
EPI ISL 174321 NA A/Ostrich/Netherlands/03006814/2003 H2N3
EPI ISL 3914 NA A/mallard/Italy/36/1999 H2N3
EPI ISL 70556 NA A/duck/Korea/BC10/2007 H7N3
EPI ISL 122074 NA A/mallard/Sw eden/57/2002 H2N3
EPI ISL 30877 NA A/duck/Altai/1285/1991 H5N3
EPI ISL 10304 NA A/cinnamon teal/Bolivia/4537/2001 H7N3
EPI ISL 67718 NA A/chicken/Chile/184240-1/2002 H7N3
EPI ISL 22600 NA A/herring gull/Norw ay/10 1623/2006 H16N3
EPI ISL 22599 NA A/common gull/Norw ay/10 1617/2006 H16N3
0.05

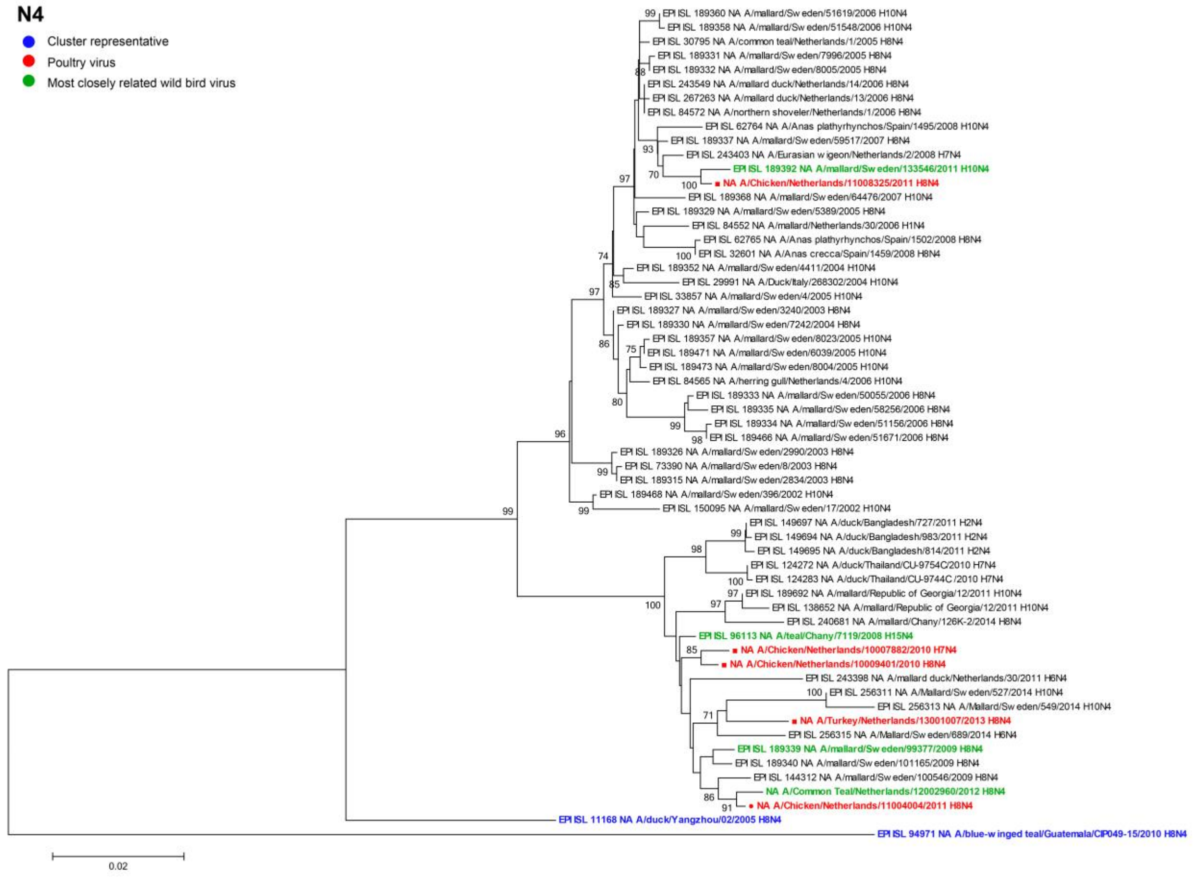
N4
Cluster representative
Poultry virus
Most closely related wild bird virus
EPI ISL 189360 NA A/mallard/Sw eden/51619/2006 H10N4
EPI ISL 189358 NA A/mallard/Sw eden/51548/2006 H10N4
EPI ISL 30795 NA A/common teal/Netherlands/1/2005 H8N4
EPI ISL 189331 NA A/mallard/Sw eden/7996/2005 H8N4
EPI ISL 189332 NA A/mallard/Sw eden/8005/2005 H8N4
EPI ISL 243549 NA A/mallard duck/Netherlands/14/2006 H8N4
EPI ISL 267263 NA A/mallard duck/Netherlands/13/2006 H8N4
EPI ISL 84572 NA A/northern shoveler/Netherlands/1/2006 H8N4
EPI ISL 62764 NA A/Anas plathyrhynchos/Spain/1495/2008 H10N4
EPI ISL 189337 NA A/mallard/Sw eden/59517/2007 H8N4
EPI ISL 243403 NA A/Eurasian w igeon/Netherlands/2/2008 H7N4
EPI ISL 189392 NA A/mallard/Sw eden/133546/2011 H10N4
NA A/Chicken/Netherlands/11008325/2011 H8N4
EPI ISL 189368 NA A/mallard/Sw eden/64476/2007 H10N4
EPI ISL 189329 NA A/mallard/Sw eden/5389/2005 H8N4
EPI ISL 84552 NA A/mallard/Netherlands/30/2006 H1N4
EPI ISL 62765 NA A/Anas plathyrhynchos/Spain/1502/2008 H8N4
EPI ISL 32601 NA A/Anas crecca/Spain/1459/2008 H8N4
EPI ISL 189352 NA A/mallard/Sw eden/4411/2004 H10N4
EPI ISL 29991 NA A/Duck/Italy/268302/2004 H10N4
EPI ISL 33857 NA A/mallard/Sw eden/4/2005 H10N4
EPI ISL 189327 NA A/mallard/Sw eden/3240/2003 H8N4
EPI ISL 189330 NA A/mallard/Sw eden/7242/2004 H8N4
EPI ISL 189357 NA A/mallard/Sw eden/8023/2005 H10N4
EPI ISL 189471 NA A/mallard/Sw eden/6039/2005 H10N4
EPI ISL 189473 NA A/mallard/Sw eden/8004/2005 H10N4
EPI ISL 84565 NA A/herring gull/Netherlands/4/2006 H10N4
EPI ISL 189333 NA A/mallard/Sw eden/50055/2006 H8N4
EPI ISL 189335 NA A/mallard/Sw eden/58256/2006 H8N4
EPI ISL 189334 NA A/mallard/Sw eden/51156/2006 H8N4
EPI ISL 189466 NA A/mallard/Sw eden/51671/2006 H8N4
EPI ISL 189326 NA A/mallard/Sw eden/2990/2003 H8N4
EPI ISL 73390 NA A/mallard/Sw eden/8/2003 H8N4
EPI ISL 189315 NA A/mallard/Sw eden/2834/2003 H8N4
EPI ISL 189468 NA A/mallard/Sw eden/396/2002 H10N4
EPI ISL 150095 NA A/mallard/Sw eden/17/2002 H10N4
EPI ISL 149697 NA A/duck/Bangladesh/727/2011 H2N4
EPI ISL 149694 NA A/duck/Bangladesh/983/2011 H2N4
EPI ISL 149695 NA A/duck/Bangladesh/814/2011 H2N4
EPI ISL 124272 NA A/duck/Thailand/CU-9754C/2010 H7N4
EPI ISL 124283 NA A/duck/Thailand/CU-9744C /2010 H7N4
EPI ISL 189692 NA A/mallard/Republic of Georgia/12/2011 H10N4
EPI ISL 138652 NA A/mallard/Republic of Georgia/12/2011 H10N4
EPI ISL 240681 NA A/mallard/Chany/126K-2/2014 H8N4
EPI ISL 96113 NA A/teal/Chany/7119/2008 H15N4
NA A/Chicken/Netherlands/10007882/2010 H7N4
NA A/Chicken/Netherlands/10009401/2010 H8N4
EPI ISL 243398 NA A/mallard duck/Netherlands/30/2011 H6N4
EPI ISL 256311 NA A/Mallard/Sw eden/527/2014 H10N4
EPI ISL 256313 NA A/Mallard/Sw eden/549/2014 H10N4
NA A/Turkey/Netherlands/13001007/2013 H8N4
EPI ISL 256315 NA A/Mallard/Sw eden/689/2014 H6N4
EPI ISL 189339 NA A/mallard/Sw eden/99377/2009 H8N4
EPI ISL 189340 NA A/mallard/Sw eden/101165/2009 H8N4
EPI ISL 144312 NA A/mallard/Sw eden/100546/2009 H8N4
NA A/Common Teal/Netherlands/12002960/2012 H8N4
NA A/Chicken/Netherlands/11004004/2011 H8N4
EPI ISL 11168 NA A/duck/Yangzhou/02/2005 H8N4
EPI ISL 94971 NA A/blue-w inged teal/Guatemala/CIP049-15/2010 H8N4
0.02

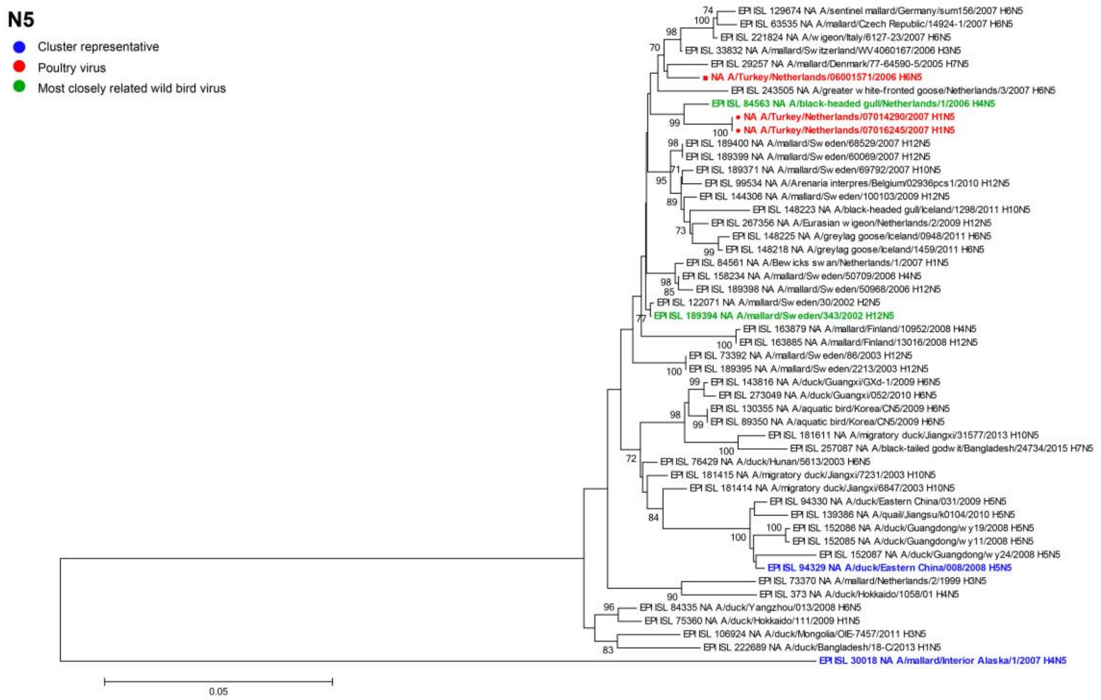
N5
Cluster representative
Poultry virus
Most closely related wild bird virus
EPI ISL 129674 NA A/sentinel mallard/Germany/sum156/2007 H6N5
EPI ISL 63535 NA A/mallard/Czech Republic/14924-1/2007 H6N5
EPI ISL 221824 NA A/wigeon/Italy/6127-23/2007 H6N5
EPI ISL 33832 NA A/mallard/Switzerland/WV4060167/2006 H3N5
EPI ISL 29257 NA A/mallard/Denmark/77-64590-5/2005 H7N5
NA A/Turkey/Netherlands/06001571/2006 H6N5
EPI ISL 243505 NA A/greater white-fronted goose/Netherlands/3/2007 H6N5
EPI ISL 84563 NA A/black-headed gull/Netherlands/1/2006 H4N5
NA A/Turkey/Netherlands/07014290/2007 H1N5
NA A/Turkey/Netherlands/07016245/2007 H1N5
EPI ISL 189400 NA A/mallard/Sweden/68529/2007 H12N5
EPI ISL 189399 NA A/mallard/Sweden/60069/2007 H12N5
EPI ISL 189371 NA A/mallard/Sweden/69792/2007 H10N5
EPI ISL 99534 NA A/Arenaria interpres/Belgium/02936pcs1/2010 H12N5
EPI ISL 144306 NA A/mallard/Sweden/100103/2009 H12N5
EPI ISL 148223 NA A/black-headed gull/Iceland/1298/2011 H10N5
EPI ISL 267356 NA A/Eurasian wigeon/Netherlands/2/2009 H12N5
EPI ISL 148225 NA A/greylag goose/Iceland/0948/2011 H6N5
EPI ISL 148218 NA A/greylag goose/Iceland/1459/2011 H6N5
EPI ISL 84561 NA A/Bewicks swan/Netherlands/1/2007 H1N5
EPI ISL 158234 NA A/mallard/Sweden/50709/2006 H4N5
EPI ISL 189398 NA A/mallard/Sweden/50968/2006 H12N5
EPI ISL 122071 NA A/mallard/Sweden/30/2002 H2N5
EPI ISL 189394 NA A/mallard/Sweden/343/2002 H12N5
EPI ISL 163879 NA A/mallard/Finland/10952/2008 H4N5
EPI ISL 163885 NA A/mallard/Finland/13016/2008 H12N5
EPI ISL 73392 NA A/mallard/Sweden/86/2003 H12N5
EPI ISL 189395 NA A/mallard/Sweden/2213/2003 H12N5
EPI ISL 143816 NA A/duck/Guangxi/GXd-1/2009 H6N5
EPI ISL 273049 NA A/duck/Guangxi/052/2010 H6N5
EPI ISL 130355 NA A/aquatic bird/Korea/CN5/2009 H6N5
EPI ISL 89350 NA A/aquatic bird/Korea/CN5/2009 H6N5
EPI ISL 181611 NA A/migratory duck/Jiangxi/31577/2013 H10N5
EPI ISL 257087 NA A/black-tailed godwit/Bangladesh/24734/2015 H7N5
EPI ISL 76429 NA A/duck/Hunan/5613/2003 H6N5
EPI ISL 181415 NA A/migratory duck/Jiangxi/7231/2003 H10N5
EPI ISL 181414 NA A/migratory duck/Jiangxi/6847/2003 H10N5
EPI ISL 94330 NA A/duck/Eastern China/031/2009 H5N5
EPI ISL 139386 NA A/quail/Jiangsu/k0104/2010 H5N5
EPI ISL 152086 NA A/duck/Guangdong/wy19/2008 H5N5
EPI ISL 152085 NA A/duck/Guangdong/wy11/2008 H5N5
EPI ISL 152087 NA A/duck/Guangdong/wy24/2008 H5N5
EPI ISL 94329 NA A/duck/Eastern China/008/2008 H5N5
EPI ISL 73370 NA A/mallard/Netherlands/2/1999 H3N5
EPI ISL 373 NA A/duck/Hokkaido/1058/01 H4N5
EPI ISL 84335 NA A/duck/Yangzhou/013/2008 H6N5
EPI ISL 75360 NA A/duck/Hokkaido/111/2009 H1N5
EPI ISL 106924 NA A/duck/Mongolia/OIE-7457/2011 H3N5
EPI ISL 222689 NA A/duck/Bangladesh/18-C/2013 H1N5
EPI ISL 30018 NA A/mallard/Interior Alaska/1/2007 H4N5
0.05

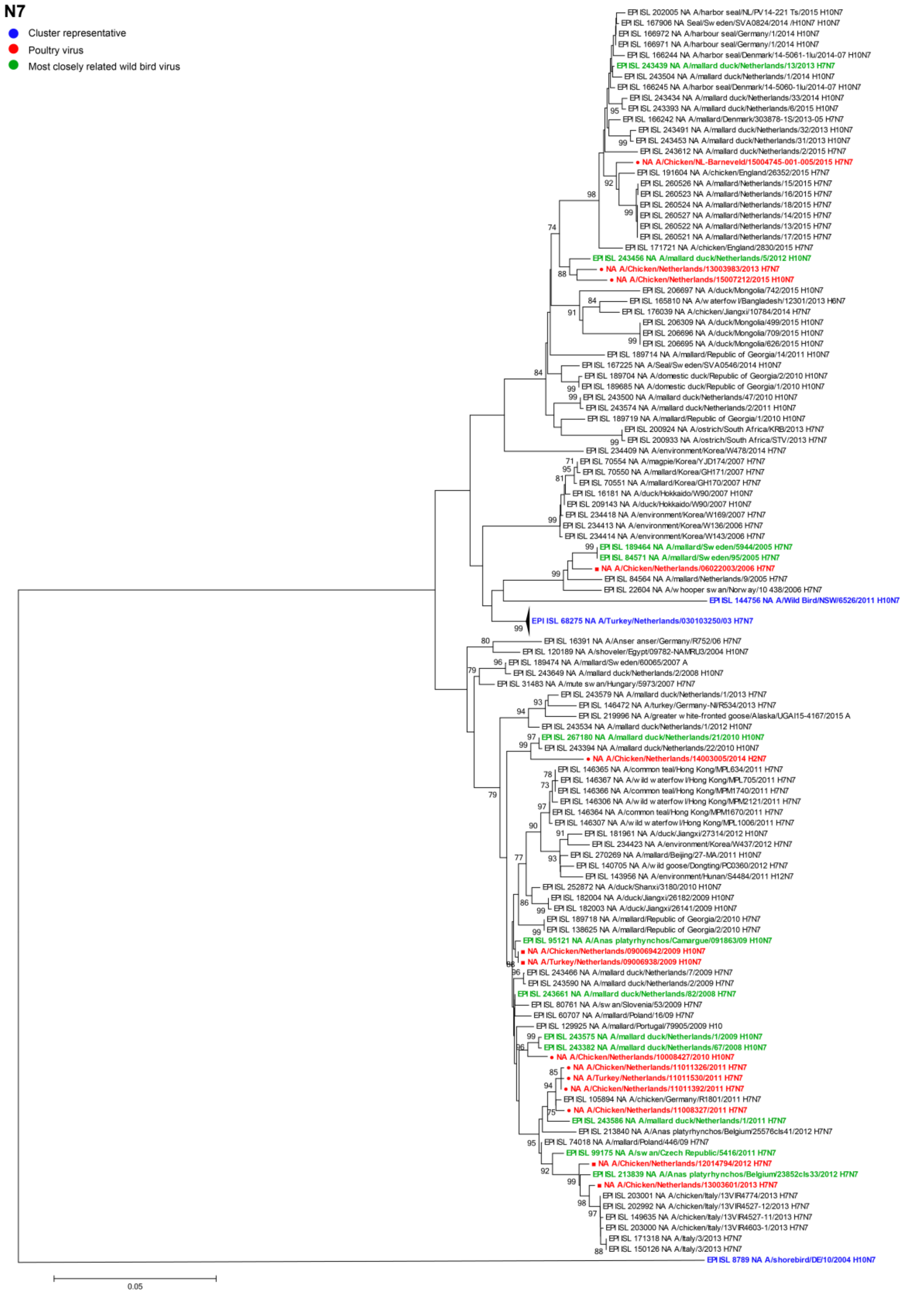

N7
Cluster representative
Poultry virus
Most closely related wild bird virus
EPI ISL 202005 NA A/harbor seal/NL/PV14-221 Ts/2015 H10N7
EPI ISL 167906 NA Seal/Sweden/SVA0824/2014 /H10N7 H10N7
EPI ISL 166972 NA A/harbour seal/Germany/1/2014 H10N7
EPI ISL 166971 NA A/harbour seal/Germany/1/2014 H10N7
EPI ISL 166244 NA A/harbor seal/Denmark/14-5061-1lu/2014-07 H10N7
EPI ISL 243439 NA A/mallard duck/Netherlands/13/2013 H7N7
EPI ISL 243504 NA A/mallard duck/Netherlands/1/2014 H10N7
EPI ISL 166245 NA A/harbor seal/Denmark/14-5060-1lu/2014-07 H10N7
EPI ISL 243434 NA A/mallard duck/Netherlands/33/2014 H10N7
EPI ISL 243393 NA A/mallard duck/Netherlands/6/2015 H10N7
EPI ISL 166242 NA A/mallard/Denmark/303878-1S/2013-05 H7N7
EPI ISL 243491 NA A/mallard duck/Netherlands/32/2013 H10N7
EPI ISL 243453 NA A/mallard duck/Netherlands/31/2013 H10N7
EPI ISL 243612 NA A/mallard duck/Netherlands/2/2015 H7N7
NA A/Chicken/NL-Barneveld/15004745-001-005/2015 H7N7
EPI ISL 191604 NA A/chicken/England/26352/2015 H7N7
EPI ISL 260526 NA A/mallard/Netherlands/15/2015 H7N7
EPI ISL 260523 NA A/mallard/Netherlands/16/2015 H7N7
EPI ISL 260524 NA A/mallard/Netherlands/18/2015 H7N7
EPI ISL 260527 NA A/mallard/Netherlands/14/2015 H7N7
EPI ISL 260522 NA A/mallard/Netherlands/13/2015 H7N7
EPI ISL 260521 NA A/mallard/Netherlands/17/2015 H7N7
EPI ISL 171721 NA A/chicken/England/2830/2015 H7N7
EPI ISL 243456 NA A/mallard duck/Netherlands/5/2012 H10N7
NA A/Chicken/Netherlands/13003983/2013 H7N7
NA A/Chicken/Netherlands/15007212/2015 H10N7
EPI ISL 206697 NA A/duck/Mongolia/742/2015 H10N7
EPI ISL 165810 NA A/waterfowl/Bangladesh/12301/2013 H6N7
EPI ISL 176039 NA A/chicken/Jiangxi/10784/2014 H10N7
EPI ISL 206309 NA A/duck/Mongolia/499/2015 H10N7
EPI ISL 206696 NA A/duck/Mongolia/709/2015 H10N7
EPI ISL 206695 NA A/duck/Mongolia/626/2015 H10N7
EPI ISL 189714 NA A/mallard/Republic of Georgia/14/2011 H10N7
EPI ISL 167225 NA A/Seal/Sweden/SVA0546/2014 H10N7
EPI ISL 189704 NA A/domestic duck/Republic of Georgia/2/2010 H10N7
EPI ISL 189685 NA A/domestic duck/Republic of Georgia/1/2010 H10N7
EPI ISL 243500 NA A/mallard duck/Netherlands/47/2010 H10N7
EPI ISL 243574 NA A/mallard duck/Netherlands/2/2011 H10N7
EPI ISL 189719 NA A/mallard/Republic of Georgia/1/2010 H10N7
EPI ISL 200924 NA A/ostrich/South Africa/KRB/2013 H7N7
EPI ISL 200933 NA A/ostrich/South Africa/STV/2013 H7N7
EPI ISL 234409 NA A/environment/Korea/W478/2014 H7N7
EPI ISL 70554 NA A/magpie/Korea/YJD174/2007 H7N7
EPI ISL 70550 NA A/mallard/Korea/GH171/2007 H7N7
EPI ISL 70551 NA A/mallard/Korea/GH170/2007 H7N7
EPI ISL 16181 NA A/duck/Hokkaido/W90/2007 H10N7
EPI ISL 209143 NA A/duck/Hokkaido/W90/2007 H10N7
EPI ISL 234418 NA A/environment/Korea/W169/2007 H7N7
EPI ISL 234413 NA A/environment/Korea/W136/2006 H7N7
EPI ISL 234414 NA A/environment/Korea/W143/2006 H7N7
EPI ISL 189464 NA A/mallard/Sweden/5944/2005 H7N7
EPI ISL 84571 NA A/mallard/Sweden/95/2005 H7N7
NA A/Chicken/Netherlands/06022003/2006 H7N7
EPI ISL 84564 NA A/mallard/Netherlands/9/2005 H7N7
EPI ISL 22604 NA A/whooper swan/Norway/10 438/2006 H7N7
EPI ISL 144756 NA A/Wild Bird/NSW/6526/2011 H10N7
EPI ISL 68275 NA A/Turkey/Netherlands/030103250/03 H7N7
EPI ISL 16391 NA A/Anser anser/Germany/R752/06 H7N7
EPI ISL 120189 NA A/shoveler/Egypt/09782-NAMRU3/2004 H10N7
EPI ISL 189474 NA A/mallard/Sweden/60065/2007 A
EPI ISL 243649 NA A/mallard duck/Netherlands/2/2008 H10N7
EPI ISL 31483 NA A/mute swan/Hungary/5973/2007 H7N7
EPI ISL 243579 NA A/mallard duck/Netherlands/1/2013 H7N7
EPI ISL 146472 NA A/turkey/Germany-NI/R534/2013 H7N7
EPI ISL 219996 NA A/greater white-fronted goose/Alaska/UGAI15-4167/2015 A
EPI ISL 243534 NA A/mallard duck/Netherlands/1/2012 H10N7
EPI ISL 267180 NA A/mallard duck/Netherlands/21/2010 H10N7
EPI ISL 243394 NA A/mallard duck/Netherlands/22/2010 H10N7
NA A/Chicken/Netherlands/14003005/2014 H2N7
EPI ISL 146365 NA A/common teal/Hong Kong/MPL634/2011 H7N7
EPI ISL 146367 NA A/wild waterfowl/Hong Kong/MPL705/2011 H7N7
EPI ISL 146366 NA A/common teal/Hong Kong/MPM1740/2011 H7N7
EPI ISL 146306 NA A/wild waterfowl/Hong Kong/MPM2121/2011 H7N7
EPI ISL 146364 NA A/common teal/Hong Kong/MPM1670/2011 H7N7
EPI ISL 146307 NA A/wild waterfowl/Hong Kong/MPL1006/2011 H7N7
EPI ISL 181961 NA A/duck/Jiangxi/27314/2012 H10N7
EPI ISL 234423 NA A/environment/Korea/W437/2012 H7N7
EPI ISL 270269 NA A/mallard/Beijing/27-MA/2011 H10N7
EPI ISL 140705 NA A/wild goose/Dongting/PC0360/2012 H7N7
EPI ISL 143956 NA A/environment/Hunan/S4484/2011 H12N7
EPI ISL 252872 NA A/duck/Shanxi/3180/2010 H10N7
EPI ISL 182004 NA A/duck/Jiangxi/26182/2009 H10N7
EPI ISL 182003 NA A/duck/Jiangxi/26141/2009 H10N7
EPI ISL 189718 NA A/mallard/Republic of Georgia/2/2010 H7N7
EPI ISL 138625 NA A/mallard/Republic of Georgia/2/2010 H7N7
EPI ISL 95121 NA A/Anas platyrhynchos/Camargue/091863/09 H10N7
NA A/Chicken/Netherlands/09006942/2009 H10N7
NA A/Turkey/Netherlands/09006938/2009 H10N7
EPI ISL 243466 NA A/mallard duck/Netherlands/7/2009 H7N7
EPI ISL 243590 NA A/mallard duck/Netherlands/2/2009 H7N7
EPI ISL 243661 NA A/mallard duck/Netherlands/82/2008 H7N7
EPI ISL 80761 NA A/swan/Slovenia/53/2009 H7N7
EPI ISL 60707 NA A/mallard/Poland/16/09 H7N7
EPI ISL 129925 NA A/mallard/Portugal/79905/2009 H10
EPI ISL 243575 NA A/mallard duck/Netherlands/1/2009 H10N7
EPI ISL 243382 NA A/mallard duck/Netherlands/67/2008 H10N7
NA A/Chicken/Netherlands/10008427/2010 H10N7
NA A/Chicken/Netherlands/11011326/2011 H7N7
NA A/Turkey/Netherlands/11011530/2011 H7N7
NA A/Chicken/Netherlands/11011392/2011 H7N7
EPI ISL 105894 NA A/chicken/Germany/R1801/2011 H7N7
NA A/Chicken/Netherlands/11008327/2011 H7N7
EPI ISL 243586 NA A/mallard duck/Netherlands/1/2011 H7N7
EPI ISL 213840 NA A/Anas platyrhynchos/Belgium/25576cls41/2012 H7N7
EPI ISL 74018 NA A/mallard/Poland/446/09 H7N7
EPI ISL 99175 NA A/swan/Czech Republic/5416/2011 H7N7
NA A/Chicken/Netherlands/12014794/2012 H7N7
EPI ISL 213839 NA A/Anas platyrhynchos/Belgium/23852cls33/2012 H7N7
NA A/Chicken/Netherlands/13003601/2013 H7N7
EPI ISL 203001 NA A/chicken/Italy/13VIR4774/2013 H7N7
EPI ISL 202992 NA A/chicken/Italy/13VIR4527-12/2013 H7N7
EPI ISL 149635 NA A/chicken/Italy/13VIR4527-11/2013 H7N7
EPI ISL 203000 NA A/chicken/Italy/13VIR4603-1/2013 H7N7
EPI ISL 171318 NA A/Italy/3/2013 H7N7
EPI ISL 150126 NA A/Italy/3/2013 H7N7
EPI ISL 8789 NA A/shorebird/DE/10/2004 H10N7
0.05

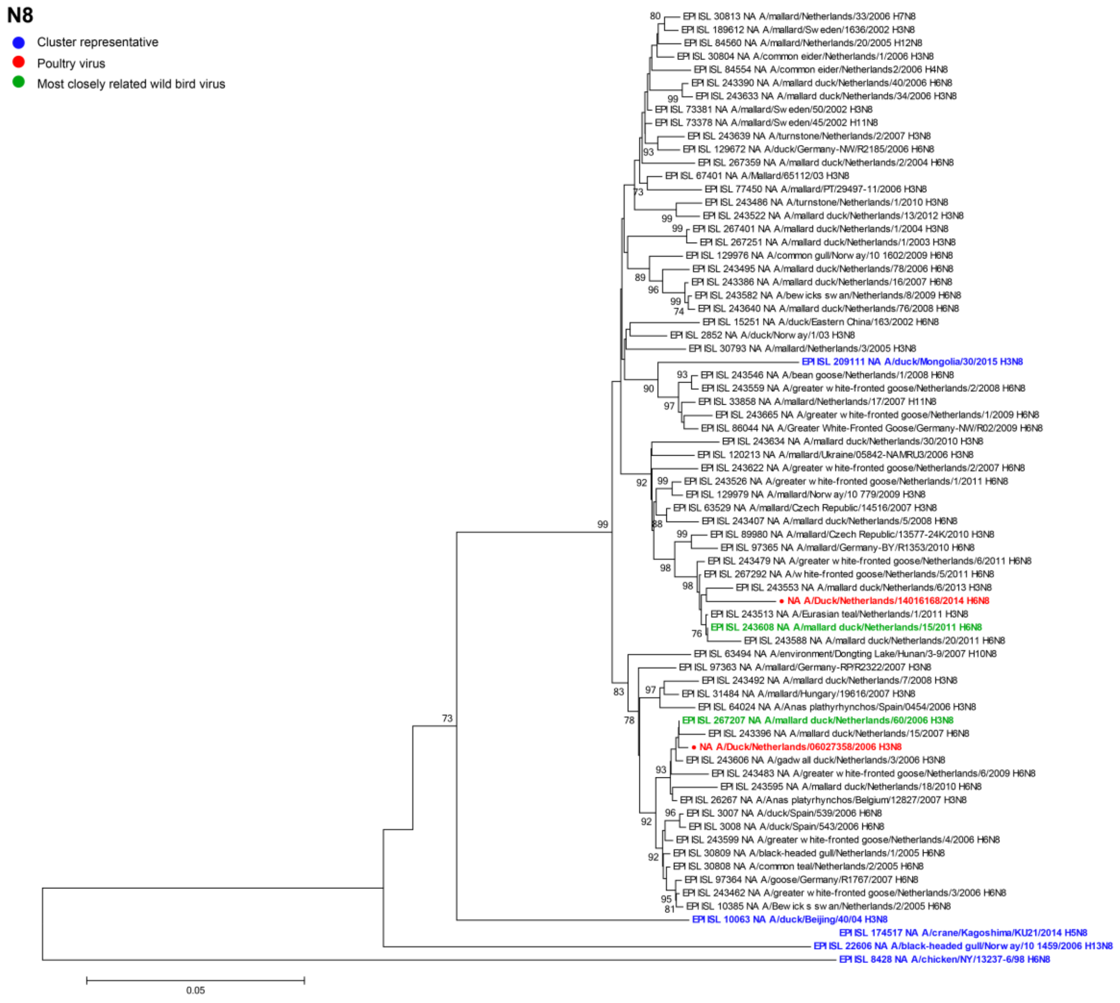

N8
Cluster representative
Poultry virus
Most closely related wild bird virus
EPI ISL 30813 NA A/mallard/Netherlands/33/2006 H7N8
EPI ISL 189612 NA A/mallard/Sw eden/1636/2002 H3N8
EPI ISL 84560 NA A/mallard/Netherlands/20/2005 H12N8
EPI ISL 30804 NA A/common eider/Netherlands/1/2006 H3N8
EPI ISL 84554 NA A/common eider/Netherlands2/2006 H4N8
EPI ISL 243390 NA A/mallard duck/Netherlands/40/2006 H6N8
EPI ISL 243633 NA A/mallard duck/Netherlands/34/2006 H3N8
EPI ISL 73381 NA A/mallard/Sw eden/50/2002 H3N8
EPI ISL 73378 NA A/mallard/Sw eden/45/2002 H11N8
EPI ISL 243639 NA A/turnstone/Netherlands/2/2007 H3N8
EPI ISL 129672 NA A/duck/Germany-NW/R2185/2006 H6N8
EPI ISL 267359 NA A/mallard duck/Netherlands/2/2004 H6N8
EPI ISL 67401 NA A/Mallard/65112/03 H3N8
EPI ISL 77450 NA A/mallard/PT/29497-11/2006 H3N8
EPI ISL 243486 NA A/turnstone/Netherlands/1/2010 H3N8
EPI ISL 243522 NA A/mallard duck/Netherlands/13/2012 H3N8
EPI ISL 267401 NA A/mallard duck/Netherlands/1/2004 H3N8
EPI ISL 267251 NA A/mallard duck/Netherlands/1/2003 H3N8
EPI ISL 129976 NA A/common gull/Norw ay/10 1602/2009 H6N8
EPI ISL 243495 NA A/mallard duck/Netherlands/78/2006 H6N8
EPI ISL 243386 NA A/mallard duck/Netherlands/16/2007 H6N8
EPI ISL 243582 NA A/bew icks sw an/Netherlands/8/2009 H6N8
EPI ISL 243640 NA A/mallard duck/Netherlands/76/2008 H6N8
EPI ISL 15251 NA A/duck/Eastern China/163/2002 H6N8
EPI ISL 2852 NA A/duck/Norw ay/1/03 H3N8
EPI ISL 30793 NA A/mallard/Netherlands/3/2005 H3N8
EPI ISL 209111 NA A/duck/Mongolia/30/2015 H3N8
EPI ISL 243546 NA A/bean goose/Netherlands/1/2008 H6N8
EPI ISL 243559 NA A/greater w hite-fronted goose/Netherlands/2/2008 H6N8
EPI ISL 33858 NA A/mallard/Netherlands/17/2007 H11N8
EPI ISL 243665 NA A/greater w hite-fronted goose/Netherlands/1/2009 H6N8
EPI ISL 86044 NA A/Greater White-Fronted Goose/Germany-NW/R02/2009 H6N8
EPI ISL 243634 NA A/mallard duck/Netherlands/30/2010 H3N8
EPI ISL 120213 NA A/mallard/Ukraine/05842-NAMRU3/2006 H3N8
EPI ISL 243622 NA A/greater w hite-fronted goose/Netherlands/2/2007 H6N8
EPI ISL 243526 NA A/greater w hite-fronted goose/Netherlands/1/2011 H6N8
EPI ISL 129979 NA A/mallard/Norw ay/10 779/2009 H3N8
EPI ISL 63529 NA A/mallard/Czech Republic/14516/2007 H3N8
EPI ISL 243407 NA A/mallard duck/Netherlands/5/2008 H6N8
EPI ISL 89980 NA A/mallard/Czech Republic/13577-24K/2010 H3N8
EPI ISL 97365 NA A/mallard/Germany-BY/R1353/2010 H6N8
EPI ISL 243479 NA A/greater w hite-fronted goose/Netherlands/6/2011 H6N8
EPI ISL 267292 NA A/w hite-fronted goose/Netherlands/5/2011 H6N8
EPI ISL 243553 NA A/mallard duck/Netherlands/6/2013 H3N8
NA A/Duck/Netherlands/14016168/2014 H6N8
EPI ISL 243513 NA A/Eurasian teal/Netherlands/1/2011 H3N8
EPI ISL 243608 NA A/mallard duck/Netherlands/15/2011 H6N8
EPI ISL 243588 NA A/mallard duck/Netherlands/20/2011 H6N8
EPI ISL 63494 NA A/environment/Dongting Lake/Hunan/3-9/2007 H10N8
EPI ISL 97363 NA A/mallard/Germany-RP/R2322/2007 H3N8
EPI ISL 243492 NA A/mallard duck/Netherlands/7/2008 H3N8
EPI ISL 31484 NA A/mallard/Hungary/19616/2007 H3N8
EPI ISL 64024 NA A/Anas plathyrhynchos/Spain/0454/2006 H3N8
EPI ISL 267207 NA A/mallard duck/Netherlands/60/2006 H3N8
EPI ISL 243396 NA A/mallard duck/Netherlands/15/2007 H6N8
NA A/Duck/Netherlands/06027358/2006 H3N8
EPI ISL 243606 NA A/gadw all duck/Netherlands/3/2006 H3N8
EPI ISL 243483 NA A/greater w hite-fronted goose/Netherlands/6/2009 H6N8
EPI ISL 243595 NA A/mallard duck/Netherlands/18/2010 H6N8
EPI ISL 26267 NA A/Anas platyrhynchos/Belgium/12827/2007 H3N8
EPI ISL 3007 NA A/duck/Spain/539/2006 H6N8
EPI ISL 3008 NA A/duck/Spain/543/2006 H6N8
EPI ISL 243599 NA A/greater w hite-fronted goose/Netherlands/4/2006 H6N8
EPI ISL 30809 NA A/black-headed gull/Netherlands/1/2005 H6N8
EPI ISL 30808 NA A/common teal/Netherlands/2/2005 H6N8
EPI ISL 97364 NA A/goose/Germany/R1767/2007 H6N8
EPI ISL 243462 NA A/greater w hite-fronted goose/Netherlands/3/2006 H6N8
EPI ISL 10385 NA A/Bew ick s sw an/Netherlands/2/2005 H6N8
EPI ISL 10063 NA A/duck/Beijing/40/04 H3N8
EPI ISL 174517 NA A/crane/Kagoshima/KU21/2014 H5N8
EPI ISL 22606 NA A/black-headed gull/Norw ay/10 1459/2006 H13N8
EPI ISL 8428 NA A/chicken/NY/13237-6/98 H6N8
0.05

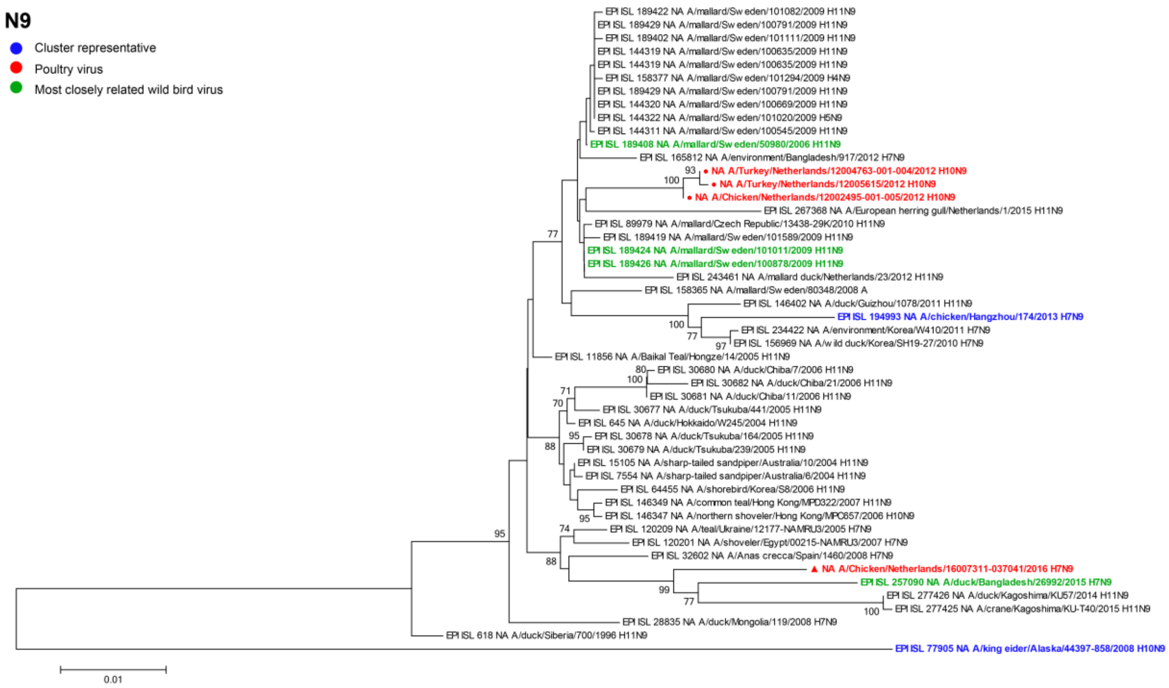
N9
Cluster representative
Poultry virus
Most closely related wild bird virus
EPI ISL 189422 NA A/mallard/Sweden/101082/2009 H11N9
EPI ISL 189429 NA A/mallard/Sweden/100791/2009 H11N9
EPI ISL 189402 NA A/mallard/Sweden/101111/2009 H11N9
EPI ISL 144319 NA A/mallard/Sweden/100635/2009 H11N9
EPI ISL 144319 NA A/mallard/Sweden/100635/2009 H11N9
EPI ISL 158377 NA A/mallard/Sweden/101294/2009 H4N9
EPI ISL 189429 NA A/mallard/Sweden/100791/2009 H11N9
EPI ISL 144320 NA A/mallard/Sweden/100669/2009 H11N9
EPI ISL 144322 NA A/mallard/Sweden/101020/2009 H5N9
EPI ISL 144311 NA A/mallard/Sweden/100545/2009 H11N9
EPI ISL 189408 NA A/mallard/Sweden/50980/2006 H11N9
EPI ISL 165812 NA A/environment/Bangladesh/917/2012 H7N9
93
NA A/Turkey/Netherlands/12004763-001-004/2012 H10N9
100
NA A/Turkey/Netherlands/12005615/2012 H10N9
NA A/Chicken/Netherlands/12002495-001-005/2012 H10N9
EPI ISL 267368 NA A/European herring gull/Netherlands/1/2015 H11N9
EPI ISL 89979 NA A/mallard/Czech Republic/13438-29K/2010 H11N9
77
EPI ISL 189419 NA A/mallard/Sweden/101589/2009 H11N9
EPI ISL 189424 NA A/mallard/Sweden/101011/2009 H11N9
EPI ISL 189426 NA A/mallard/Sweden/100878/2009 H11N9
EPI ISL 243461 NA A/mallard duck/Netherlands/23/2012 H11N9
EPI ISL 158365 NA A/mallard/Sweden/80348/2008 A
EPI ISL 146402 NA A/duck/Guizhou/1078/2011 H11N9
EPI ISL 194993 NA A/chicken/Hangzhou/174/2013 H7N9
100
EPI ISL 234422 NA A/environment/Korea/W410/2011 H7N9
77
97
EPI ISL 156969 NA A/wild duck/Korea/SH19-27/2010 H7N9
EPI ISL 11856 NA A/Baikal Teal/Hongze/14/2005 H11N9
80
EPI ISL 30680 NA A/duck/Chiba/7/2006 H11N9
100
EPI ISL 30682 NA A/duck/Chiba/21/2006 H11N9
71
EPI ISL 30681 NA A/duck/Chiba/11/2006 H11N9
70
EPI ISL 30677 NA A/duck/Tsukuba/441/2005 H11N9
EPI ISL 645 NA A/duck/Hokkaido/W245/2004 H11N9
95
EPI ISL 30678 NA A/duck/Tsukuba/164/2005 H11N9
88
EPI ISL 30679 NA A/duck/Tsukuba/239/2005 H11N9
EPI ISL 15105 NA A/sharp-tailed sandpiper/Australia/10/2004 H11N9
EPI ISL 7554 NA A/sharp-tailed sandpiper/Australia/6/2004 H11N9
EPI ISL 64455 NA A/shorebird/Korea/S8/2006 H11N9
EPI ISL 146349 NA A/common teal/Hong Kong/MPD322/2007 H11N9
95
EPI ISL 146347 NA A/northern shoveler/Hong Kong/MPC657/2006 H10N9
95
74
EPI ISL 120209 NA A/teal/Ukraine/12177-NAMRU3/2005 H7N9
EPI ISL 120201 NA A/shoveler/Egypt/00215-NAMRU3/2007 H7N9
EPI ISL 32602 NA A/Anas crecca/Spain/1460/2008 H7N9
88
NA A/Chicken/Netherlands/16007311-037041/2016 H7N9
EPI ISL 257090 NA A/duck/Bangladesh/26992/2015 H7N9
99
EPI ISL 277426 NA A/duck/Kagoshima/KU57/2014 H11N9
77
100
EPI ISL 277425 NA A/crane/Kagoshima/KU-T40/2015 H11N9
EPI ISL 28835 NA A/duck/Mongolia/119/2008 H7N9
EPI ISL 618 NA A/duck/Siberia/700/1996 H11N9
EPI ISL 77905 NA A/king eider/Alaska/44397-858/2008 H10N9
0.01
